# Two-Component Elements Mediate Interactions between Cytokinin and Salicylic Acid in Plant Immunity

**DOI:** 10.1371/journal.pgen.1002448

**Published:** 2012-01-26

**Authors:** Cristiana T. Argueso, Fernando J. Ferreira, Petra Epple, Jennifer P. C. To, Claire E. Hutchison, G. Eric Schaller, Jeffery L. Dangl, Joseph J. Kieber

**Affiliations:** 1 Biology Department, University of North Carolina, Chapel Hill, North Carolina, United States of America; 2 Howard Hughes Medical Institute, Chapel Hill, North Carolina, United States of America; 3 Department of Biological Sciences, Dartmouth College, Hanover, New Hampshire, United States of America; Virginia Tech, United States of America

## Abstract

Recent studies have revealed an important role for hormones in plant immunity. We are now beginning to understand the contribution of crosstalk among different hormone signaling networks to the outcome of plant–pathogen interactions. Cytokinins are plant hormones that regulate development and responses to the environment. Cytokinin signaling involves a phosphorelay circuitry similar to two-component systems used by bacteria and fungi to perceive and react to various environmental stimuli. In this study, we asked whether cytokinin and components of cytokinin signaling contribute to plant immunity. We demonstrate that cytokinin levels in Arabidopsis are important in determining the amplitude of immune responses, ultimately influencing the outcome of plant–pathogen interactions. We show that high concentrations of cytokinin lead to increased defense responses to a virulent oomycete pathogen, through a process that is dependent on salicylic acid (SA) accumulation and activation of defense gene expression. Surprisingly, treatment with lower concentrations of cytokinin results in increased susceptibility. These functions for cytokinin in plant immunity require a host phosphorelay system and are mediated in part by type-A response regulators, which act as negative regulators of basal and pathogen-induced SA–dependent gene expression. Our results support a model in which cytokinin up-regulates plant immunity via an elevation of SA–dependent defense responses and in which SA in turn feedback-inhibits cytokinin signaling. The crosstalk between cytokinin and SA signaling networks may help plants fine-tune defense responses against pathogens.

## Author Summary

Plant hormones play an important role in many aspects of a plant's life cycle, from the regulation of development to responses to constantly changing environmental conditions. In the past decade, the importance of hormones in plant immunity against a variety of pathogens has been uncovered. In this manuscript, we demonstrate that in the model plant species *Arabidopsis thaliana* components of the signaling system of the plant hormone cytokinin also mediate plant immunity. We demonstrate that this involves the type-A class of Arabidopsis response regulators in a process that occurs downstream of the plant defense hormone salicylic acid and involves a host two-component phosphorelay. Moreover, we show that the levels of cytokinin are important in determining the amplitude of plant immunity, ultimately influencing the outcome of plant–pathogen interactions. Finally, our results indicate that salicylic acid negatively regulates cytokinin signaling, which may serve to fine-tune the effects of cytokinin in plant immunity. Given the high energy costs of defense responses and the role of cytokinins in carbon partitioning and energy allocation, we hypothesize that the mechanisms uncovered here may help regulate the levels of energy that can be allocated into defense responses, an important aspect in the biology of plants.

## Introduction

The first layer of active plant immunity begins with the recognition of microbial molecules, followed by activation of an effective defense response [1]. Non-adapted pathogens are halted by this defense response, whereas adapted pathogens are able to overcome these defense responses via deployment of virulence factors, eventually leading to manipulation of the host biology and culminating in pathogen growth and reproduction. The plant hormones salicylic acid (SA), jasmonic acid and ethylene have long been implicated in defense responses [2] and recent studies have also uncovered a role in plant defense for several other hormones [3], [4], but the extent of crosstalk among the hormonal networks in plant defense is only now beginning to be understood.

**Figure 1:**
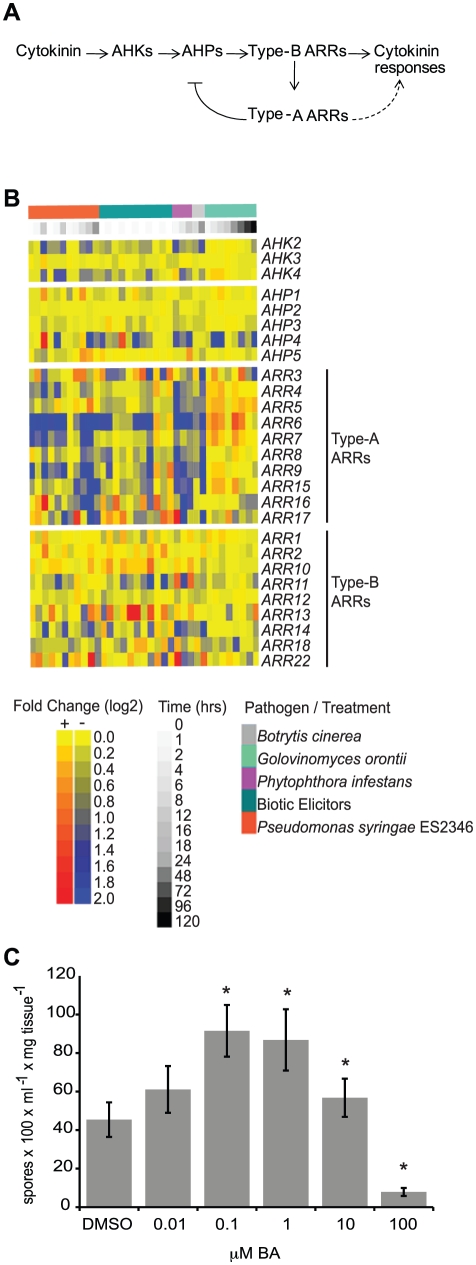
Cytokinin and two-component elements play a role in plant immunity. (A) Model of the cytokinin signaling pathway in Arabidopsis. Arrows indicate positive interactions, bar indicates a negative interaction. (B) Heat map of gene expression of two-component elements in Arabidopsis following pathogen or elicitor treatment. Microarray data was obtained from AtGenExpress (http://www.uni-tuebingen.de/plantphys/AFGN/atgenex.htm) and analyzed using the e-northern tool of the Bio-Array Resource for Arabidopsis Functional Genomics (http://bar.utoronto.ca/). (C) Susceptibility of cytokinin-treated Arabidopsis to *Hpa* Noco2. Two-week-old wild-type plants were sprayed with the indicated concentrations of BA or DMSO control 48 hours prior to inoculation with *Hpa* Noco2. Spore production was measured as described in Methods. Error bars represent SE (n = 6). Asterisks indicate statistically significant differences from wild-type plants (p-value<0.05, two-tailed student's t-test). The experiment was repeated at least three times independently. Data from one representative experiment are shown.

Cytokinins are a group of *N* ^6^-substituted adenine derivatives that regulate many plant developmental processes and responses to the environment [5]. Cytokinin perception and signaling is carried out by two-component element proteins [6], analogous to two-component signaling systems present in bacteria and fungi (Figure 1A). In Arabidopsis, binding of cytokinin to sensor histidine kinases (AHKs) receptors initiates a phosphotransfer cascade that culminates in the phosphorylation of response regulator proteins (ARRs), which are responsible for the regulation of cytokinin outputs. ARRs fall into two main groups [6]: type-A ARRs contain short C-terminal extensions and act as negative regulators of cytokinin responses [7]–[10]; type-B ARRs contain extended C-termini that include a DNA binding domain and directly mediate the transcription of cytokinin-responsive genes and positively regulate cytokinin signaling [7], [11]–[13].

Several lines of evidence support a role for cytokinins in plant-pathogen interactions. For example, application of cytokinin results in decreased replication of White Clover Mosaic Potexvirus and in the induction of defense gene expression in bean plants [14]. Treatment of Arabidopsis plants or plant cell cultures with cytokinin also up-regulates stress- and defense-related genes [15], [16] and promotes resistance to the bacterial pathogen *Pseudomonas syringae* pv. *tomato* DC3000 (*Pst* DC3000) in a process involving a type-B ARR and SA signaling [17]. Conversely, increases in cytokinin content are associated with the formation of ‘green-islands’, photosynthetically active leaf tissue supporting a region of pathogen growth and surrounded by senescent tissue [18]. Increased cytokinin content is associated with increased pathogen growth in several plant species [19], [20]. Finally, many fungal and bacterial pathogens can produce cytokinins [21], presumably used to manipulate host cell physiology to the pathogen's benefit. These examples suggest that levels of pathogen- or host-derived cytokinins can alter host responses to pathogens and influence the outcome of plant-pathogen interactions.

Here, we report that exogenous cytokinin alters immune responses of Arabidopsis to a pathogenic isolate of a biotrophic oomycete pathogen. We show that while high concentrations of cytokinin lead to decreased susceptibility through a process that requires SA accumulation and activation of SA-dependent defense gene expression, treatment with lower concentrations of cytokinin results in increased pathogen growth. We also demonstrate that SA negatively regulates cytokinin signaling, which may act to fine-tune this process. These functions for cytokinin in plant defense require an intact host cytokinin phosphorelay system, and are mediated in part by type-A ARRs, which act as negative regulators of defense responses.

## Results

### Pathogen infection and elicitor treatment alter the expression of cytokinin signaling components in Arabidopsis

We examined the expression of Arabidopsis genes encoding elements involved in cytokinin signal transduction (Figure 1A) in response to pathogen treatment. Publicly available microarray data deposited at AtGenExpress (http://www.uni-tuebingen.de/plantphys/AFGN/atgenex.htm) were analyzed using the e-northern tool of the Bio-Array Resource for Arabidopsis Functional Genomics (http://bar.utoronto.ca/) [22] (Figure 1B). Among the genes encoding two-component elements, the expression levels of type-A *ARR* genes were most affected by pathogen treatment. This is similar to what is observed after treatment of plants with exogenous cytokinin [16], [23]. While the expression of genes encoding two-component elements was clearly altered by pathogen treatment, there was no direct correlation between the pattern of gene expression and pathogen lifestyle with respect to biotrophic (*G. orontii*), hemibiotrophic (*P. syringae* ES2346, *P. infestans*) or necrotrophic (*B. cinerea*) pathogens, or elicitors derived from biotrophic pathogens. These results are consistent with a role for two-component elements in the response to a variety of plant pathogens.

### Cytokinin treatment alters susceptibility of Arabidopsis plants to *Hyaloperonospora arabidopsidis* isolate Noco2

Due to contrasting reports regarding the role of cytokinins during plant immune system responses [18], we examined the effect of a range of concentrations of exogenous cytokinin on the responses of wild-type Arabidopsis plants (accession Col-0) to the virulent oomycete *Hyaloperonospora arabidopsidis* isolate Noco2 (*Hpa* Noco2). *Hpa* Noco2 is a well-adapted obligate biotrophic pathogen of Arabidopsis that is able to overcome defense responses of wild-type plants and establish an intimate relationship with its host. Two-week-old plants were treated with increasing concentrations of the cytokinin benzyl adenine (BA) or a vehicle control (DMSO) 48 hours prior to pathogen treatment (Figure 1C). We observed distinctive effects of cytokinin on the susceptibility of wild-type plants to *Hpa* Noco2 at the different concentrations tested. Treatment with low concentrations of exogenous cytokinin led to enhanced susceptibility to *Hpa* Noco2, indicating that cytokinin-dependent processes contribute to the susceptibility to this pathogen (Figure 1C). In contrast, treatment with higher levels of cytokinin (>10 µM) led to decreased susceptibility to *Hpa* Noco2, indicating a threshold above which the action of cytokinin has a negative impact on the susceptibility of Arabidopsis to *Hpa* Noco2 (Figure 1C).

### Treatment with high concentrations of cytokinin primes SA–dependent defense responses in Arabidopsis

**Figure 2:**
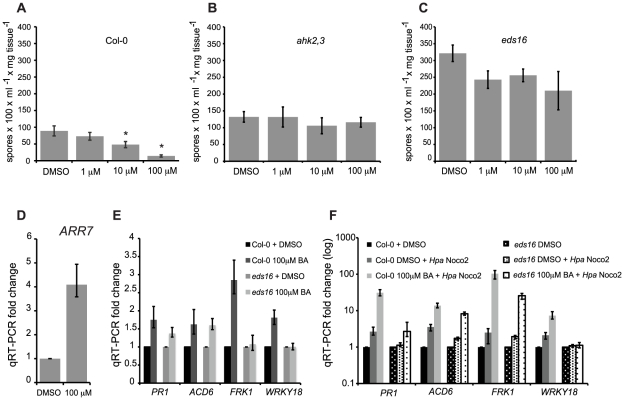
High concentrations of cytokinin prime defense responses via SA accumulation. Susceptibility of cytokinin-treated wild-type (A), *ahk2,3* (B) or *eds16* (C) plants to *Hpa* Noco2. Two-week-old plants were sprayed with the indicated concentrations of BA or DMSO control 48 hours prior to inoculation with *Hpa* Noco2. Spore production was measured as described in Methods. Error bars represent SE (n≥4). Asterisks indicate statistically significant differences from wild-type plants (p-value<0.05, two-tailed student's t-test). The experiment was conducted in parallel for all genotypes above and repeated at least three times independently. Data from one representative experiment are shown. (D) *ARR7* expression in response to cytokinin treatment. RNA was extracted from two-week-old wild-type plants from (A) that had been sprayed with the indicated concentration of BA or DMSO control, 48 hours after treatment. Levels of *ARR7* were determined by qRT-PCR relative to DMSO samples. Error bars represent SE from three technical replicates and correspond to upper and lower limits of 95% confidence intervals. At least three independent biological replicates of the experiment were conducted with similar results. Data from one representative independent biological replicate are shown. (E) Expression of defense genes after cytokinin treatment. Two-week-old wild-type and *eds16* plants were treated with the indicated concentration of BA or DMSO. RNA was extracted from tissue 48 hours after treatment. Transcripts levels were determined by qRT-PCR relative to samples treated with DMSO. Error bars represent SE from three technical replicates and correspond to upper and lower limits of 95% confidence intervals. At least three independent biological replicates of the experiment were conducted with similar results. Data from one representative independent biological replicate are shown. (F) Defense gene expression is enhanced by pre-treatment with cytokinin. Two-week-old wild-type and *eds16* plants were pre-treated with the indicated concentration of BA or DMSO control 48 hours prior to inoculation with water or *Hpa* Noco2. RNA was extracted from tissue harvested at 3 dpi. Transcripts levels were determined by qRT-PCR relative to the respective DMSO-treated genotypes. Error bars represent SE from three technical replicates and correspond to upper and lower limits of 95% confidence intervals. At least three independent biological replicates of the experiment were conducted with similar results. Data from one representative independent biological replicate are shown.

We further investigated the effect of cytokinin on plant immunity. Pre-treatment of wild-type plants with high concentrations of cytokinin led to decreased susceptibility to *Hpa* Noco2 (Figure 2A). The added cytokinin induced a cytokinin response as shown by the up-regulation of the cytokinin-inducible gene *ARR7* (Figure 2D). The effect of high levels of cytokinin on the growth of *Hpa* Noco2 was not due to off-target effects of BA or direct effects on *Hpa* Noco2 growth as it was abrogated by disruption of the *AHK2* and *AHK3* cytokinin receptors (Figure 2B). Comparable levels of cytokinin have been shown to elicit biologically relevant levels of cytokinin signaling in other assays for cytokinin responses [24], [25]. Two non-mutually exclusive hypotheses that could account for this effect of cytokinin on *Hpa* Noco2 susceptibility are: 1) changes in host metabolism that could result in poor pathogen growth; or 2) increased activation of defense responses. Because SA plays a significant role in plant immunity [26], [27], we tested if this response to high concentrations of cytokinin was a result of activation of SA-mediated responses by examining the *eds16* mutant in which the *ISOCHORISMATE SYNTHASE 1* (*ICS1*) gene required for SA biosynthesis [28], [29] is mutated. *eds16* plants displayed a substantially reduced response to high concentrations (100 µM) of cytokinin (Figure 2C) as compared to wild-type plants. This indicates that the effect of high concentrations of cytokinin is largely dependent on SA biosynthesis, which is consistent with a role for cytokinin upstream of SA during activation of defense responses by *Hpa* Noco2. Interestingly, there is a slight decrease in pathogen growth in the *eds16* plants at lower cytokinin concentrations (1 µM) that is not observed in wild-type plants, suggesting that *eds16* plants are hypersensitive to cytokinin (Figure 2C; see also below).

To further understand the relationship of high concentrations of cytokinin and plant immunity, we analyzed the expression of SA-responsive genes in response to cytokinin treatment and inoculation with *Hpa* Noco2 (Figure 2E, 2F). While much defense transcriptional reprogramming generally occurs early after pathogen recognition [30], [31], we chose to look at gene expression changes three days post inoculation (dpi) when an estimated 40% of plant mesophyll cells are in contact with *Hpa* hyphae and/or haustoria [32]. The SA-responsive genes tested were marginally up-regulated by cytokinin treatment alone in wild-type plants (Figure 2E). This increase in expression in response to cytokinin was partially dependent on SA biosynthesis as it was generally diminished in *eds16* plants. As expected, the defense genes examined were markedly up-regulated by inoculation with *Hpa* Noco2 in the wild-type, but to a reduced extent in *eds16* plants (Figure 2F). While cytokinin treatment alone only led to a slight induction in defense gene expression, pre-treatment with cytokinin followed by *Hpa* Noco2 inoculation led to a further enhancement of the expression of the defense genes tested (Figure 2F). These results suggest that cytokinin acts by priming defense-gene expression in Arabidopsis. This potentiation of defense gene expression by cytokinin was partially dependent on SA as revealed by gene expression analysis of similarly treated *eds16* plants (Figure 2E, 2F).

### SA negatively regulates cytokinin signaling

**Figure 3:**
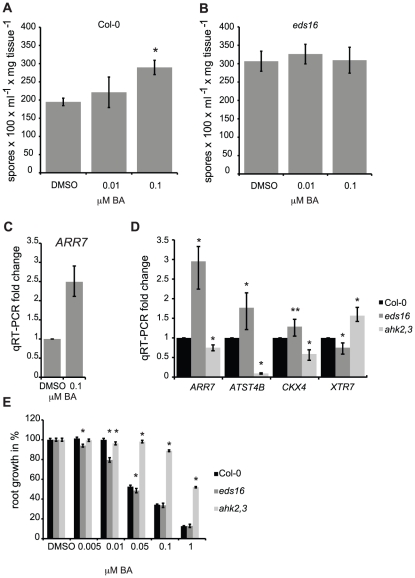
SA negatively regulates cytokinin signaling. Susceptibility of cytokinin-treated wild-type (A) and *eds16* (B) plants to *Hpa* Noco2. Two-week-old plants were sprayed with the indicated concentrations of BA or DMSO control 48 hours prior to inoculation with *Hpa* Noco2. Spore production was measured as described in Methods. Error bars represent SE (n≥4). Asterisks indicate statistically significant differences from wild-type plants (p-value<0.05, two-tailed student's t-test). The experiment was conducted in parallel for all genotypes above and repeated at least three times independently. Data from one representative experiment are shown. (C) *ARR7* expression in response to cytokinin treatment. RNA was extracted from two-week-old wild-type plants from (A) that had been sprayed with the indicated concentration of BA or DMSO control, 48 hours after treatment. Levels of *ARR7* were determined by qRT-PCR relative to DMSO samples. Error bars represent SE from three technical replicates and correspond to upper and lower limits of 95% confidence intervals. At least three independent biological replicates of the experiment were conducted with similar results. Data from one representative independent biological replicate are shown. (D) Basal expression of cytokinin-regulated genes in wild-type, *eds16* and *ahk2,3* plants. RNA was extracted from tissue harvested from untreated two-week-old seedlings. Levels of transcripts were determined by qRT-PCR relative to wild-type samples. Error bars represent SE from three technical replicates and correspond to upper and lower limits of 95% confidence intervals. At least three independent biological replicates of the experiment were conducted with similar results. Data from one representative independent biological replicate are shown. Statistically significant differences from wild-type plants (one-tailed student's t-test) are represented by asterisks (* = p-value<0.05, ** = p-value<0.075). (E) Primary root elongation assay for cytokinin sensitivity. Wild-type, *eds16* or *ahk2,3* seedlings were grown vertically on plates supplemented with the specified concentrations of BA or DMSO control under constant light conditions at 23°C. Primary root elongation between days 4 and 9 was measured as described in Methods. Results shown were pooled from an experimental set of three independent samples of 10 to 15 individual seedlings each. Asterisks indicate statistically significant differences from the wild type at the given concentrations of BA (two-tailed student's t-test, P<0.05). Error bars represent SE (n≥22). This experiment was repeated twice with consistent results.

Treatment of wild-type plants with low concentrations of cytokinin (100 nM), which are sufficient to induce expression of the cytokinin-regulated gene *ARR7* (Figure 3C), results in increased susceptibility to *Hpa* Noco2 (Figure 1C, Figure 3A). We examined if the effect of low concentrations of cytokinin on susceptibility to *Hpa* Noco2 was dependent on endogenous SA, as was observed for higher levels of cytokinin. *eds16* plants did not show an increase in pathogen growth after cytokinin treatment (Figure 3B), suggesting that basal levels of SA may be required for the promotion of susceptibility by low levels of cytokinin. Alternatively, the hyper-susceptible phenotype of *eds16* plants [28] may preclude quantification of marginal increases in the growth of pathogens.

We examined the effect of SA on cytokinin responsiveness by examining the expression of cytokinin-regulated genes [16] in untreated *eds16* plants. The basal level of expression of genes positively regulated by cytokinin (*ARR7*, *ATST4B*, and to a lesser extent *CKX4*; [16]) were significantly elevated in *eds16* plants relative to the wild-type. Conversely, the expression of *XTR7 (XYLOGLUCAN ENDOTRANSGLUCOSYLASE 7)*, which is negatively regulated by cytokinin, was further down-regulated in *eds16* relative to the wild-type (Figure 3D). We also analyzed the response of *eds16* plants to cytokinin using a primary root elongation assay [10], [33]. Wild-type seedlings showed inhibition of root elongation by BA concentrations above 50 nM, while *ahk2,4* control plants were largely resistant to BA. *eds16* plants displayed a significant and reproducible hypersensitivity to cytokinin at lower concentrations of cytokinin (Figure 3E). Together, these results suggest that SA negatively regulates cytokinin responsiveness, consistent with the hypersensitivity of *eds16* mutants to cytokinin with regard to pathogen growth (Figure 2C).

### Mutations in two-component elements alter susceptibility to *Hpa* Noco2

**Figure 4:**
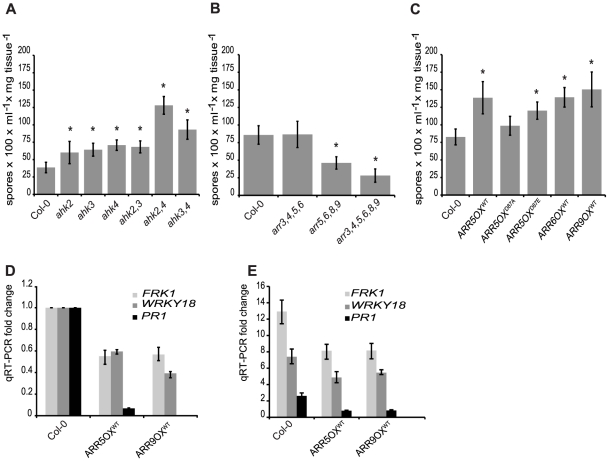
A two-component phosphorelay, negatively regulated by type-A ARRs, is required for defense responses. (A) Susceptibility of *ahk* receptor single and double mutants to *Hpa* Noco2. Two-week-old plants were inoculated with *Hpa* Noco2 and spore production measured as described in Methods. Error bars represent SE (n≥4). Asterisks indicate statistically significant differences from wild-type plants (p-value<0.05, two-tailed student's t-test). The experiment was repeated at least three times independently. Data from one representative experiment are shown. (B) Susceptibility of type-A *arr* multiple mutants to *Hpa* Noco2. Two-week-old plants were inoculated with *Hpa* Noco2 and spore production measured as described in Methods. Error bars represent SE (n≥4). Asterisks indicate statistically significant differences from wild-type plants (p-value<0.05, two-tailed student's t-test). The experiment was repeated at least three times independently. Data from one representative experiment are shown. (C) Susceptibility of transgenic lines overexpressing type-A ARRs to *Hpa* Noco2. Two-week-old wild-type plants or transgenic lines overexpressing wild-type (*ARR5*, *ARR6*, *ARR9*), phospho-mimic (*ARR5^D87E^*) and phospho-deficient (*ARR5^D87A^*) forms of type-A ARRs were inoculated with *Hpa* Noco2 and spore production measured as described in Methods. Error bars represent SE (n≥4). Asterisks indicate statistically significant differences from wild-type plants (p-value<0.05, two-tailed student's t-test). The experiment was repeated at least three times independently. Data from one representative experiment are shown. (D) Basal defense gene expression in transgenic lines overexpressing type-A *ARRs*. Two-week-old wild-type plants or transgenic lines overexpressing wild-type type-A *ARRs* (*ARR5* and *ARR9*) were inoculated with water. RNA was extracted from tissue harvested three days later. Levels of the indicated transcripts were determined by qRT-PCR relative to wild-type plants. Error bars represent SE from three technical replicates and correspond to upper and lower limits of 95% confidence intervals. At least three independent biological replicates of the experiment were conducted with similar results. Data from one representative independent biological replicate are shown. (E) Defense gene expression in response to *Hpa* Noco2 in transgenic lines overexpressing type-A *ARRs*. Two-week-old wild-type plants or transgenic lines overexpressing wild-type type-A *ARRs* (*ARR5* and *ARR9*) were inoculated with water or *Hpa* Noco2. RNA was extracted from tissue harvested at 3 dpi. Levels of the indicated transcripts were determined by qRT-PCR relative to water-treated samples. Error bars represent SE from three technical replicates and correspond to upper and lower limits of 95% confidence intervals. At least three independent biological replicates of the experiment were conducted with similar results. Data from one representative independent biological replicate are shown.

To further address the mechanism of cytokinin action in plant immunity, we examined the requirement for a host phosphorelay mechanism in the susceptibility of Arabidopsis to *Hpa* Noco2. Consistent with a role for cytokinin in plant immunity, disruption of the cytokinin receptors (*AHK2*, *AHK3* or *AHK4*) resulted in enhanced susceptibility to *Hpa* Noco2 (Figure 4A). The *ahk3,4* and *ahk2,4* double mutants, but not the *ahk2,3* double mutant, displayed an additive increase in susceptibility, indicating that the cytokinin receptors play partially redundant roles in defense responses to this pathogen, similar to their overlapping roles in other cytokinin-regulated physiological processes [34]–[36]. The triple receptor mutant was not used in this study due to its small, stunted phenotype, which precludes us from drawing any meaningful conclusions from pathogen assays in this background.

Unlike the other two-component elements, type-A ARRs are negative regulators of cytokinin signaling [7]–[10]. There are ten genes encoding type-A ARRs in Arabidopsis. Due to partial redundancy in this gene family, increased sensitivity to cytokinin is apparent only in quadruple and higher order multiple mutants. The type-A *arr* multiple mutants *arr5,6,8,9* and *arr3,4,5,6,8,9* showed decreased susceptibility to *Hpa* Noco2 (Figure 4B) as compared to wild-type plants. Similar to their roles in cytokinin signaling, the respective single mutations had no measurable effect on susceptibility to *Hpa* Noco2 (data not shown). Interestingly, the *arr3,4,5,6* mutant, which has an equivalent hypersensitivity to cytokinin as the *arr5,6,8,9* mutant in several response assays [10], did not exhibit any difference in susceptibility to *Hpa* Noco2 compared to wild-type plants, suggesting combinatorial specificity in this response (Figure 4B). Together, these results indicate that cytokinin signaling components play partially overlapping roles in plant immunity; the cytokinin receptors exert a mainly positive role, while the type-A ARRs have a negative regulatory effect.

### Type-A *ARRs* negatively regulate plant immunity in a phospho-dependent manner

To further explore the role of type-A ARRs in plant immunity, we examined the effect of overexpression of type-A *ARRs* on the susceptibility to *Hpa* Noco2. Consistent with the decreased susceptibility phenotype observed in the loss-of-function type-A *arr* multiple mutants, transgenic lines overexpressing type-A *ARR* genes under the control of the constitutive CaMV *35S* promoter [33] showed enhanced susceptibility (Figure 4C). This suggests that susceptibility to *Hpa* Noco2 is correlated to the level of type-A ARRs. Phosphorylation of type-A ARRs on a conserved residue (Asp87) in the receiver domain is required for type-A ARR activation and function in cytokinin signaling [33]. Therefore, we tested whether this phosphorylation is required for the enhanced susceptibility to *Hpa* Noco2 seen in transgenic lines overexpressing type-A ARRs. Transgenic lines overexpressing phospho-mimic (ARR5^D87E^) and phospho-deficient (ARR5^D87A^) forms of ARR5 were tested for their susceptibility to *Hpa* Noco2. These lines have been characterized and shown to express similar protein levels, and the ARR5^D87E^ and ARR5^D87A^ proteins have been shown to retain their ability to interact with two-component elements in a yeast two-hybrid assay, indicating proper folding [33]. Similar to lines overexpressing wild-type type-A *ARRs*, overexpression of ARR5^D87E^ also led to enhanced susceptibility to *Hpa* Noco2 (Figure 4C). Conversely, plants overexpressing ARR5^D87A^ were not statistically significantly different from wild-type plants in their susceptibility to *Hpa* Noco2 (Figure 4C). Moreover, the expression levels of the defense genes tested were reduced in both unchallenged (Figure 4D) and *Hpa*-induced (Figure 4E) *ARR* overexpressing lines in comparison to wild-type plants. These results indicate that it is the phosphorylated form of type-A ARRs that play a negative role in regulating defense responses in both unchallenged plants and in response to *Hpa* Noco2.

### Type-A ARRs negatively regulate SA–dependent basal immunity

**Figure 5:**
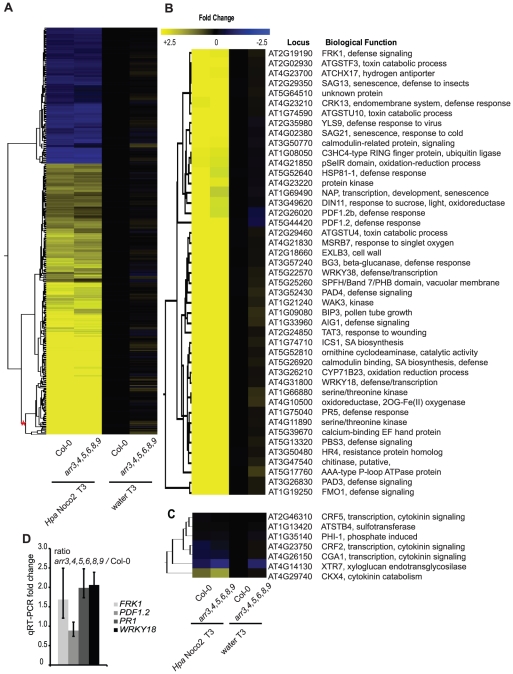
Type-A ARRs negatively regulate SA–dependent gene expression. (A) Transcriptome analysis of type-A *arr3,4,5,6,8,9* mutant plants in response to *Hpa* Noco2. Two-week-old wild-type or type-A *arr3,4,5,6,8,9* mutant plants were inoculated with either water or *Hpa* Noco2. Tissue was harvested at 3 dpi. For the analysis, wild-type water-treated samples were used as a baseline. Genes up- or down-regulated at least two-fold by *Hpa* Noco2 in wild-type plants were selected. Hierarchical clustering (K-means) of *Hpa* Noco2-regulated genes in wild-type plants is shown. See also Table S1. (B) Subset of *Hpa* Noco2-regulated genes with altered expression in the *arr3,4,5,6,8,9* mutants. *Hpa* Noco2-regulated genes from the most highly regulated cluster from (A) (red asterisk) that are differentially regulated in unchallenged *arr3,4,5,6,8,9* mutant plants. (C) Representative cytokinin-regulated genes that are also *Hpa* Noco2-regulated. (D) qRT-PCR of select genes from (A). Two-week-old wild-type or *arr3,4,5,6,8,9* plants were inoculated with water. RNA was extracted from tissue harvested three days later. Levels of the indicated transcripts were determined by qRT-PCR relative to wild-type plants. Error bars represent SE from three technical replicates and correspond to upper and lower limits of 95% confidence intervals. Data from one biological replicate are shown.

The potential role of type-A ARRs in basal defense gene expression and *Hpa* Noco2-triggered responses led us to investigate transcriptional reprogramming in response to *Hpa* Noco2 in the type-A *arr3,4,5,6,8,9* multiple mutant. Wild-type and *arr3,4,5,6,8,9* mutant plants were treated with either water or inoculated with *Hpa* Noco2 and tissue harvested three days after treatment. RNA from replicate samples from independent experiments was prepared and gene expression analyzed using ATH1 Affymetrix microarrays. Samples were normalized to the water-treated wild-type samples. The expression levels of 1583 genes were significantly altered in wild-type plants in response to inoculation with *Hpa* Noco2 (Figure 5A). Transcriptome changes were similar in wild-type and *arr3,4,5,6,8,9* mutant plants in response to *Hpa* Noco2, both in amplitude and in the set of genes regulated (Table S1). However, 292 of these regulated genes were expressed at levels 20–50% higher in water-treated *arr3,4,5,6,8,9* mutant plants as compared to water-treated wild-type plants; hence they are under negative control by type-A ARRs. Representatives selected from the most markedly de-repressed cluster (Figure 5A, red asterisk) include several genes involved in SA-mediated defense signaling (e.g. *FRK1*, *PAD4*, *FMO1* and *WRKY18*), SA biosynthesis (*ICS1*), and SA-mediated defense markers (e.g. *PR5*). Conversely, a subset of genes known to be down-regulated by SA, such as *PDF1.2*, displayed reduced basal expression in *arr3,4,5,6,8,9* plants (Figure 5B). We confirmed these results for a subset of genes in an independent experiment using qRT-PCR (Figure 5D). These results suggest that type-A *arr3,4,5,6,8,9* mutant plants are primed for defense responses, exhibiting a slight elevation of SA-dependent defense gene expression even in the absence of applied biotic stress.

Previously described cytokinin-responsive genes [16] were also differentially regulated by *Hpa* Noco2 in wild-type plants (Figure 5C). The overlap between the suites of cytokinin- and *Hpa* Noco2-regulated genes supports a function for cytokinin in plant immunity and suggests a role for cytokinin-regulated processes in the pathogenicity of *Hpa* Noco2. The altered expression of both suites of genes in *arr3,4,5,6,8,9* mutants indicates that these processes converge at the level of type-A ARR function.

### Type-A ARRs suppress defense gene expression downstream of SA

**Figure 6:**
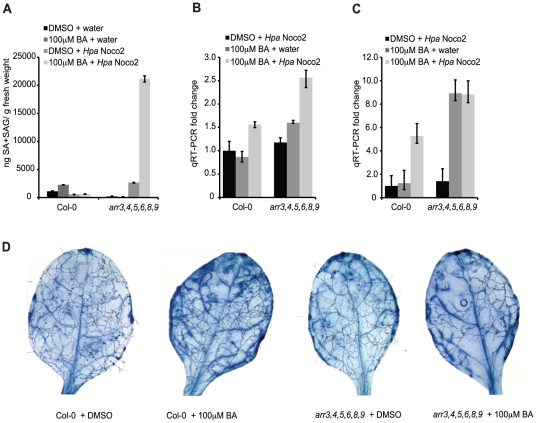
Type-A ARRs act in plant immunity downstream of SA. (A) Total SA production in response to *Hpa* Noco2 after cytokinin treatment. Two-week-old wild-type and *arr3,4,5,6,8,9* plants were pre-treated with the indicated concentration of BA or DMSO control 48 hours prior to inoculation with water or *Hpa* Noco2. Tissue was harvested at 3 dpi and total SA (SA+SAG) measured as described in Methods. Error bars represent SE (n≥4). The experiment was repeated at least three times independently. Data from one representative experiment are shown. (B) *ICS1* expression in response to *Hpa* Noco2 after cytokinin treatment. Two-week-old wild-type and *arr3,4,5,6,8,9* plants were treated as in (A). Tissue was harvested at 3 dpi. Levels of *ICS1* were determined by qRT-PCR relative to water-treated samples pre-treated with DMSO. For simplicity, the relative change of all samples was normalized to the wild-type DMSO+*Hpa* Noco2 levels. Error bars represent SE from three technical replicates and correspond to upper and lower limits of 95% confidence intervals. The experiment was repeated at least three times independently. Data from one representative experiment are shown. (C) *PR1* expression in response to *Hpa* Noco2 after cytokinin treatment. Two-week-old wild-type and *arr3,4,5,6,8,9* plants were treated as in (A). Tissue was harvested at 3 dpi. Levels of *PR1* were determined by qRT-PCR relative to water-treated samples pre-treated with DMSO. For simplicity, the relative change for all samples was normalized to the wild-type DMSO+*Hpa* Noco2 levels. Error bars represent SE from three technical replicates and correspond to upper and lower limits of 95% confidence intervals. The experiment was repeated at least three times independently. Data from one representative experiment are shown. (D) Trypan blue staining after *Hpa* Noco2 inoculation. Two-week-old wild-type and *arr3,4,5,6,8,9* plants were treated as in (A). Plants were harvested at 4 dpi and stained with lacto-phenol trypan blue to visualize pathogen structures.

Among the genes induced by *Hpa* Noco2 and de-repressed in *arr3,4,5,6,8,9* mutant plants is *ICS1*, which encodes an enzyme involved in SA biosynthesis. We hypothesized that the altered expression of SA-dependent genes observed in *arr3,4,5,6,8,9* plants is a direct result of altered regulation of *ICS1* and SA metabolism, and subsequent activation of SA-dependent defense responses. We examined SA accumulation in the wild-type and *arr3,4,5,6,8,9*, and the contribution of *Hpa* Noco2 and high concentrations of cytokinin (100 µM BA) to this response. SA levels in wild-type plants, regardless of treatment, remained at or below levels of detection of our assay. These results are similar to published results of SA levels in Arabidopsis plants after infection with virulent isolates of *Hpa* [37] and reflect the relatively weaker defense responses elicited by virulent pathogens and the nature of the *Hpa*-Arabidopsis interaction, in which a limited number of plant cells are in contact with the pathogen at early stages of infection. The SA levels of *arr3,4,5,6,8,9* plants treated with DMSO, cytokinin or *Hpa* Noco2 were also at or below the detection limits of our SA assay (Figure 6A). In contrast *arr3,4,5,6,8,9* mutant plants pre-treated with cytokinin and subsequently challenged with *Hpa* Noco2 showed a significant and reproducible increase in SA levels, well above the detection limits of our assay (Figure 6A). These results suggest that the increased defense responses observed in *arr3,4,5,6,8,9* mutant plants are due to increased SA content.

The results of our SA assays led us to examine the expression of *ICS1* and the defense marker *PR1* in these plants. As expected, *ICS1* expression was elevated synergistically by *Hpa* Noco2 and cytokinin treatment in both genotypes. *arr3,4,5,6,8,9* plants showed even further up-regulation of *ICS1*, which could account for elevated SA levels observed in these plants (Figure 6B). Surprisingly, levels of *PR1* were equally high in the *arr3,4,5,6,8,9* mutant treated with cytokinin, or with cytokinin and *Hpa* Noco2, even though levels of SA and *ICS1* differed (Figure 6C). These results indicate that in the absence of functional type-A ARRs, cytokinin can bypass the requirement for recognition of *Hpa* Noco2 on the activation of defense responses, suggesting a role for type-A ARRs in the suppression of defense responses downstream of SA accumulation. Consistent with increased defense gene expression and SA content, *arr3,4,5,6,8,9* plants treated with cytokinin also exhibited increased resistance to *Hpa* Noco2 (Figure 6D). To better score susceptibility, plants were stained with lactophenol-trypan blue at 4 dpi. At this point during infection, wild-type plants pre-treated with DMSO showed widespread hyphal growth and sporulation, while wild-type plants pre-treated with cytokinin had not yet produced sporangiophores and displayed diminished hyphal growth. DMSO-treated *arr3,4,5,6,8,9* plants showed decreased susceptibility compared to similarly-treated wild-type plants, and this was even more apparent in *arr3,4,5,6,8,9* plants pre-treated with cytokinin, which showed substantially reduced hyphal growth (Figure 6D).

## Discussion

We examined the influence of the plant hormone cytokinin on the immune responses of Arabidopsis plants in response to the biotrophic oomycete *Hpa* Noco2. The susceptibility of wild-type plants was increased after treatment with low concentrations of the cytokinin BA (<1 µM) and decreased with higher concentrations (>10 µm). This bell-shaped response is reminiscent of other physiological responses regulated by cytokinin, such as shoot initiation *in vitro* [38], [39] and the induction of ethylene biosynthesis [40]. In particular, this finding is similar to the effect of exogenous cytokinin on the response of wheat to powdery mildew (*Erysiphe graminis*), in which a complex dose response curve of pathogen growth was obtained in response to exogenous zeatin [20]. While multiple processes such as cytokinin uptake, degradation and conjugation likely contribute to the complexity of this response, our findings highlight the importance of hormone concentrations during the responses of plants to pathogens. All molecules with cytokinin activity are recognized in Arabidopsis by the three cytokinin receptors, AHK2, AHK3 and AHK4 [34], [35] that have varying affinities for different cytokinins [41]–[43]. Different cytokinins elicit different levels of cytokinin signaling upon binding to the cytokinin receptors [44]. It is thus possible that contrasting reports on the roles of cytokinin in susceptibility to pathogens might reflect the levels of signaling elicited by different cytokinins during plant-pathogen interactions and their different effects on pathogen growth, which would be similar to the effect of different levels of cytokinin on the susceptibility of Arabidopsis to *Hpa* Noco2 observed in this study.

Treatment with lower concentrations of cytokinin resulted in a significant increase in *Hpa* Noco2 growth on wild-type plants. The mechanisms involved in this increased susceptibility may involve several physiological processes that are regulated by cytokinins, such as sink-source relationships, delay of senescence and/or nutrient acquisition [5], many of which likely affect to the ability of pathogens to grow optimally. Several plant pathogens produce cytokinins in order to manipulate plant physiology and development, thereby promoting optimal conditions for completion of their life cycle [21]. The role of lower concentrations of cytokinin for the susceptibility of Arabidopsis plants to *Hpa* Noco2 raises the question of whether *Hpa*-derived cytokinins could be contributing to the growth of this pathogen. Analysis of the *Hpa* genome does not reveal any isopentenyl transferases genes predicted to synthesize cytokinins, as found in plants and some plant pathogens [45]. Genes encoding tRNA isopentenyl transferases involved in a secondary cytokinin biosynthetic pathway are present in the *Hpa* genome, as they are in most genomes, but given the debatable role of tRNA-derived cytokinins in plant physiology [45] these are unlikely to contribute in a substantial way to the production of active cytokinins.

Treatment of Arabidopsis with high levels of cytokinin led to an enhancement of defense responses, characterized by a decrease in susceptibility to *Hpa* Noco2. This effect of cytokinin was mostly abolished in *eds16* plants, demonstrating that cytokinin acts primarily upstream of SA production in plant immune responses against *Hpa* Noco2. Treatment of plants with high concentrations of cytokinin led to a subtle increase in defense gene expression, which was further enhanced after treatment with *Hpa* Noco2. Consistent with our observations, a similar effect of comparably higher concentrations of cytokinin was observed in the induction of resistance and enhancement of defense gene expression to a pathogenic strain of *Pst* DC3000 in Arabidopsis [17], and a comparable effect of cytokinin on defense gene activation was reported in tobacco plants after wounding, also accompanied by increased SA levels [46]. This potentiation of defense gene expression by pre-treatment with cytokinin observed in our results indicates that cytokinin may act by priming the defense responses of Arabidopsis plants to *Hpa* Noco2. While the molecular mechanisms of priming are not well understood, it is hypothesized that priming may pre-activate defense signaling, but not defense responses, allowing plants to respond more rapidly to biotic and abiotic stresses [47] without the energy costs associated with pre-activation of full defense responses [48]. Given the role of cytokinins in carbon partitioning and energy allocation [49], [50], it is possible that cytokinin signaling might play a role in regulating the levels of energy that can be allocated into defense responses.

**Figure 7:**
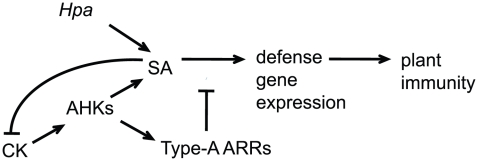
Model for cytokinin and type-A ARRs action in plant immunity. *Hpa* Noco2 is perceived by Arabidopsis plants, leading to activation of salicylic acid (SA)-dependent responses and defense gene expression. High concentrations of cytokinin (CK) potentiate SA-dependent defense gene expression leading to decreased susceptibility, in a process that is counteracted by the type-A Arabidopsis response regulators (ARRs) downstream of SA accumulation. In turn, SA inhibits cytokinin signaling, in a negative feedback mechanism that fine-tunes the process.

The effect of high cytokinin concentrations on the susceptibility to *Hpa* Noco2 required the AHK2 and AHK3 cytokinin receptors, indicating that a cytokinin phosphorelay system is required for responses to *Hpa* Noco2. Similar to other processes regulated by cytokinin, the individual receptors contribute differently to this phenotype [34]–[36]. Type-A response regulators are negative regulators of cytokinin signaling [7]–[10]. We observed that type-A *arr 3,4,5,6,8,9* multiple mutant plants exhibited decreased susceptibility to *Hpa* Noco2. While type-A ARRs exert mostly overlapping roles in cytokinin signaling, the combinatorial specificity observed in the responses of two different quadruple mutants to *Hpa* Noco2 may suggest distinct roles for individual type-A ARRs in regulation of plant immunity. Transgenic lines overexpressing type-A *ARRs* display decreased defense responses and allowed for increased pathogen growth; hence type-A ARRs are also negative regulators of plant immunity. Consistent with this conclusion, we note that type-A ARRs must function to regulate basal responses in uninfected plants, as unchallenged *arr3,4,5,6,8,9* plants display elevated basal expression of several SA-regulated genes and as we observed a converse effect on SA-dependent signaling when type-A *ARRs* are overexpressed. Overexpression of *ARR5^D87A^*, which cannot be phosphorylated, did not lead to increased susceptibility. This result indicates that it is primarily the phosphorylated state of type-A ARRs that is active in the negative regulation of SA-dependent defense responses and that a complete phosphorelay cascade, initiated at the level of cytokinin receptors and culminating in type-A ARR phosphorylation and activation, is required in this process. This type-A ARR function is promoted by cytokinin and occurs downstream of SA; in the absence of functional type-A ARRs, defense gene expression, but not SA accumulation, is elevated following cytokinin treatment (Figure 7).

While the exact mechanisms by which type-A ARRs function are still unknown, phosphorylation of their receiver domain has been shown to stabilize a subset of type-A ARR proteins [33], and likely to lead to phospho-specific interactions with target proteins, which in turn mediates cytokinin outputs [33]. A similar mechanism of response regulator action is employed in two-component systems in yeast [51]. Importantly, a type-B ARR transcription factor has also been shown to trigger enhancement of defense responses to the bacterial pathogen *Pst* DC3000. [17]. In this model, treatment of plants with comparably high concentrations of the cytokinin *trans*-zeatin leads the TGA3 b-zip transcription factor to associate with and recruit the type-B transcription factor ARR2 to specific *cis*-elements within the promoter of the *PR1* gene, thus activating defense responses [17]. It is known that the phosphorelay cascade that is initiated after cytokinin perception promotes type-B ARR phosphorylation and activation, culminating in the transcription of cytokinin-regulated genes, which include type-A *ARRs*. In the context of plant immunity, high concentrations of cytokinin may lead not only to activation ARR2 and its association with TGA3 on the *PR1* promoter, but also to the transcription of type-A *ARR* genes and their activation by phosphorylation, which might then counteract defense responses.

In addition to cytokinin up-regulating SA-dependent responses, our results suggest that SA negatively regulates cytokinin signaling. Similarly to type-A *arr* mutants, *eds16* plants showed hypersensitivity to low concentrations of cytokinin. In *Hpa* Noco2 susceptibility assays, *eds16* plants also displayed hypersensitivity to high concentrations of cytokinin as compared to wild-type plants. Taken together, these results point to a possible feedback loop of SA on cytokinin signaling that would work to fine-tune the level of defense responses to pathogens. A possible trade-off between cytokinin-regulated and SA-dependent defense responses may have broad agricultural implications. Some species of plants, such as tomato, soybeans and particularly rice, have naturally high basal levels of SA [52]. If in these crop species SA negatively influences cytokinin-regulated processes, which include nutrient allocation and yield, manipulating this hormonal crosstalk may lead to increased crop productivity.

Our results reveal a complex crosstalk between cytokinin and SA in plant immunity, in a mechanism involving two-component signaling elements and which incorporates regulation in part by type-A ARRs. Moreover, we show that cytokinin levels are important in determining the amplitude of plant immunity, ultimately influencing the outcome of plant-pathogen interactions. As the network of plant hormone interactions in plant immunity is further dissected, it is becoming clear that a for a complete appreciation of the role of plant hormones in this process, the levels of hormonal signaling will also have to be considered.

## Methods

### Plant materials and plant growth

The Col-0 accession was used as the wild-type in this study. The *ahk* T-DNA knockout mutants used in this study have been described in [53]. Type-A *arr* T-DNA knockout mutants (*arr3,4,5,6*; *arr5,6,8,9* and *arr,3,4,5,6,8,9*) and ARR-overexpressing transgenic lines have been described [10], [33]. *eds16* plants have been previously described [28]. All mutants and transgenic lines described above are in the Col-0 accession. All plants were grown on soil (Metro 360) in growth chambers (Percival Scientific) under short days (8∶16 hour light∶dark, 22°C).

### Inoculation of plants with *Hpa* Noco2

*Hpa* Noco 2 was propagated on the susceptible Col-0 accession. *Hpa* spores (5×10^4^/ml) were sprayed onto two-week-old plants using a pressurized sprayer (Preval). Inoculated plants were kept in growth chambers (Percival Scientific) (19°C, 8∶16 hour light∶dark) and covered with a transparent plastic dome to maintain high humidity. For *Hpa* assays, two-week old plants were inoculated as described above. One day after the first appearance of sporangiophores (5–6 dpi) the first pair of true leaves was collected from three individual plants, and added to a previously weighed 1.5 ml microcentrifuge tube containing 300 µl of sterile water, for a total of six leaves per sample, and weighed again to determine fresh weight. Spores were counted using a hemacytometer. Spore counts from at least four samples per genotype were determined.

### Trypan blue staining of *Hpa*-infected plants

Plants were harvested at 4 dpi and stained with a 3∶1 ethanol: lacto-phenol trypan blue solution (1∶1∶1∶1 phenol∶ lactic acid∶ water∶ glycerol and 0.05% trypan blue (Sigma-Aldrich)), at 95°C, for 5 min, and moved to room temperature for 10 min. Excess staining was removed with chloral hydrate (Sigma-Aldrich). Samples were moved to 50% glycerol for storage and mounting. Pictures were taken with an Olympus SZX9 stereomicroscope.

### Cytokinin treatment of soil-grown plants

Cytokinin (benzyl adenine, or BA) (Sigma-Aldrich) was sprayed onto two-week-old plants, using a Preval sprayer. BA solutions were prepared from a stock in DMSO, diluted into an aqueous solution to the required BA concentration plus 0.002% Silwet L-77 (Lehle Seeds). Control plants were sprayed with the corresponding amounts of DMSO plus 0.002% Silwet L-77.

### RNA extraction and qRT–PCR

Total RNA was extracted using RNeasy Plant kit (QIAGEN), according to the manufacturer's instructions. Quality and integrity of RNA were assessed by gel electrophoresis and A_260_/A_280_ and A_260_/A_230_ ratios. RNA samples of good quality were treated with DNAse-free Turbo (Ambion) and then checked for absence of genomic DNA by qRT-PCR using primers for At5g65080, (At5g65080 For 5′-TTTTTTGCCCCCTTCGAATC-3′, At5g65080 Rev 5′-ATCTTCCGCCAC-CACATTGTAC-3′). cDNA synthesis was performed using Superscript III (Invitrogen) and oligo-d(T) primers according to the manufacturer's instructions. cDNA was checked for full extension by qRT-PCR using primers for 3 amplicons 1 kB apart within At1g13320 (At1g13320a, At1g13320b, At1g13320c); primers used are as follows: At1g13320a For 5′-TAGATCGCTCGGAACTTGGAAA-3′; At1g13320a Rev 5′-GGAGTGATTTGAGTTTTGGTGAGG-3′; At1g13320b For 5′-AACTAGGACGGATCTGGTGCCT-3′; At1g13320b Rev 5′-ATAATGAGGCA-GAAGTTCGGATAGC-3′; At1g13320c For 5′-AAATTTAACGTGGCCAAAA-TGATGC-3′; At1g13320c Rev 5′-ACCAAGCGGTTGTGGAGAAC-3′. cDNAs with C_t_ ratios of At1g13320a/At1g13320b and At1g13320b/At1g13320c below 1.5 C_t_s were considered suitable for qRT-PCR. qRT-PCR reactions were performed using ExTaq SYBR Green (Takara) on a Bio-Rad Opticon2 machine using the following thermocycler program: (1) 2 min at 95°C; (2) 15 s at 95°C; (3) 15 s at 60°C; (4) 15 s at 72°C; (5) optical read, repeat 34 cycles of steps 2 through 5, followed by a final analysis of product melting temperature to confirm the PCR product. *β-TUBULIN 4* (At5g44340) was used as housekeeping gene in all reactions. Gene-specific primers are as follows: *ATST4B* (At1g13420) *For* 5′-AGCCTCGTGTGCAAA-TCAAGAGAC-3′, *Rev* 5′-ACTCCTTCCGACAAGCT-TCCTGTT-3′; *ARR7* (At1g19050) *For* 5′-ACTGTAGAGAGTGGAACTAGGGCT-3′, *Rev* 5′-AGTCCTGGCATTGAGTAATCCGTC-3′; *ICS1* (At1g74710) *For* 5′-TGCATCCAACTCCAGCTGTTTGTG-3′; *Rev* 5′-AGCTGATCTGATCCCGA-CTGCAAA-3′; *PR1* (At2g14610) *For* 5′-ACACGTGCAATGGAGTTTGTGGTC-3′; *Rev* 5′-TACACCTCACTTTGGCACATCCGA-3′; *FRK1* (At2g19190) *For* 5′-AGCTTCTCTGTTGAAGGAAGCGGT-3′; *Rev* 5′-TTGAGCTTGCAATAGC-AGGTTGGC-3′; *XTR7* (At4g14130) *For* 5′-AGCTCAATGCTTATGGCAGGAGGA-3′; *Rev* 5′-TTGCATTCTGGAGGGAAT-CCACGA-3′; *ACD6* (At4g14400) *For* 5′-GTGACGTTTG-CTGCAGGCTTTACA-3′, *Rev* 5′-AGTTGGGTTAGTGGC-CAAAGTTGC-3′; *CKX4* (At4g29740) *For* 5′-CACCCACAAGGGTGAAATGGTCTC-3′, *Rev* 5′-TGCGACTCTTGTTTGATCGGAGAG-3′; *WRKY18* (At4g31800) *For* 5′-TGGGTCAAGCACAGTGAC-TTTGGA-3′; *Rev* 5′-GCAGCAGCAAGAGC-AGCTGTAAAT-3′; *β-TUBULIN 4* (At5g44340) *For* 5′-AGAGGTTGACGAGCAA-GATGA-3′, *Rev* 5′-AACAATGAAAGTAGACGCCA-3′; *PDF1.2* (At5g44420) *For* 5′-GCTTCCATCATCACCCTTATCTTC-3′; *Rev* 5′-ACATGGGACGTAACAGATACA-CTTGTGT-3′. The relative expression of specific genes and 95% confidence intervals were determined using REST 2008; [54] (http://rest-2008.gene-quantification.info). At least three biological replicates of each experiment were obtained and qRT-PCR performed as described above.

### Primary root elongation assay for cytokinin sensitivity

Arabidopsis seeds were grown on vertical plates containing MS medium (1× MS salts, 0.05% MES buffer, and 1% sucrose, pH 5.8), with 0.6% phytagel (Sigma-Aldrich) supplemented with a dose range of BA or 0.1% (v/v) DMSO vehicle control for 10 days. Primary root lengths at days 4 and 9 were marked on the plates. The plates were scanned at 10 days, and root growth between days 4 and 9 was measured using NIH Image J version 1.43u (National Institutes of Health, Bethesda, MD).

### Microarray experiments

Two-week old plants (wild-type and *arr3,4,5,6,8,9*) grown under short days (8∶16 hour light∶dark cycle, 22°C) were sprayed with distilled water (control) or *Hpa* Noco2 as described above. Plants were kept at 18°C and 8∶16 hour light∶dark cycle. Tissue was harvested three days after treatment. Two independent biological replicates of the experiment were obtained. Total RNA was extracted using RNeasy Plant Kit (QIAGEN). 30 µg of total RNA were converted into cRNA and hybrized to ATH1 chips (Affymetrix) according to the manufacturer's instructions. Data were RMA-transformed and analyzed using Genespring software version GX 10 (Agilent). Raw values were filtered to a minimum expression of 20^th^ percentile and statistical analysis was performed with two-way ANOVA (α≤0.05) using Benjamini-Hochberg multiple testing correction. For interpretation of data, wild-type water-treated samples were used as a baseline (control) for comparison to the other samples.

### Total SA measurements

Two-week-old seedlings were pre-treated with either DMSO or cytokinin BA and subsequently inoculated with either water or *Hpa* Noco2 as in Figure 1. Tissue was harvested at 3 dpi. Total SA measurements, including free SA and SA glucoside (SAG), were performed as described [55]. Briefly, frozen samples were ground and tissue homogenized in 200 µl 0.1 M acetate buffer pH 5.6. Samples were then centrifuged for 15 min at 16,000 g at 4°C. 100 µl of supernatant was transferred to a new tube for free SA measurement, and 10 µl were incubated with 1 µl 0.5 U/µl β-glucosidase (Sigma-Aldrich) for 90 min at 37°C for total SA measurement. After incubation, 60 µl of LB, 20 µl of plant extract (treated or not with β-glucosidase), and 50 µl of *Acinetobacter* sp. ADPWH-*lux* (OD = 0.4) were added to each well of a black 96-well plate. The plate was incubated at 37°C for 60 min and luminescence was read with a Spectra Max M5 (Molecular Devices) microplate reader. For the standard curve, 1 µl of known amounts of SA stock (from 0 to 1000 µg/ml) was diluted 10-fold in *eds16* plant extract, and 5 µl of each standard were added to the wells of the plate containing 60 µl of LB, and 50 µl of *Acinetobacter* sp. ADPWH-*lux* (OD = 0.4). SA standards were read in parallel with the experimental samples. SA standard values were analyzed with linear regression for calculations of SA amounts. Results are depicted by gram of fresh weight.

## Supporting Information

Table S1 Expression levels of genes regulated by treatment with *Hyaloperonospora arabidopsidis* (*Hpa*) isolate Noco2 on wild-type (Col-0) and *arr3,4,5,6,8,9* mutant plants, 3 days after water or *Hpa* Noco2 treatment. Samples were normalized to water-treated wild-type samples. Average of technical replicates is shown.
(PDF)
