# Supplementary material for: Two-Component Elements Mediate Interactions between Cytokinin and Salicylic Acid in Plant Immunity
Source: PLoS Genet. 2012 Jan 26;8(1):e1002448. doi: 10.1371/journal.pgen.1002448 (PMC3266875; doi:10.1371/journal.pgen.1002448)
Supplement: Table S1 — Expression levels of genes regulated by treatment with Hyaloperonospora arabidopsidis (Hpa) isolate Noco2 on wild-type (Col-0) and arr3,4,5,6,8,9 mutant plants, 3 days after water or Hpa Noco2 treatment. Samples were normalized to water-treated wild-type samples. Average of technical replicates is shown. (PDF) [file pgen.1002448.s001.pdf]

Table 1 SI

Table 1 Supporting Information: Expression levels of genes regulated by treatment with *Hyaloperonospora arabidopsidis* isolate Noco2 (*Hpa*) on wild-type (Col-0) and *arr3,4,5,6,8,9* mutant plants, 3 days after water or *Hpa* Noco2 treatment. Samples were normalized to water-treated wild-type (Col-0) samples. Average of technical replicates is shown.

Probe Set ID	AGI	p-value	[Col, Noco](normalized)	[Col, water](normalized)	[arr345689, Noco](normalized)	[arr345689, water](normalized)
261585_at	AT1G01010	1.94E-03	0.85521555	0.00000000	0.80082130	-0.16989780
261025_at	AT1G01225	3.86E-03	-0.26118088	-0.00000024	-0.34199238	0.14129782
261027_at	AT1G01340	3.94E-04	2.18104740	-0.00000024	1.67296390	-0.30774070
259428_at	AT1G01560	1.61E-04	2.37648530	0.00000000	2.53437760	0.23138785
261537_at	AT1G01800	3.74E-03	0.40875626	-0.00000048	0.43995857	0.08397818
261652_at	AT1G01860	3.45E-03	-0.69661117	0.00000024	-0.42923070	-0.02835441
261624_at	AT1G02000	3.52E-03	-0.28773284	0.00000000	-0.31586170	-0.18896079
264178_at	AT1G02170	3.77E-04	1.13947820	0.00000000	0.90529010	-0.06098843
264123_at	AT1G02270	8.41E-04	0.60719633	-0.00000048	0.69164990	0.00085306
259443_at	AT1G02360	6.56E-05	2.55904480	0.00000000	2.38492870	-0.06808567
260904_at	AT1G02450	1.40E-03	2.82095480	-0.00000024	2.73717400	0.10011983
264355_at	AT1G03210	3.15E-04	0.90639780	-0.00000048	0.92998743	-0.10366297
264362_at	AT1G03290	3.84E-03	1.71733710	0.00000024	1.56455420	0.12723780
264821_at	AT1G03470	4.09E-03	-0.45647573	-0.00000024	-0.33193707	0.06080222
264837_at	AT1G03600	2.31E-03	-0.65869856	-0.00000048	-0.64146040	0.11020565
264832_at	AT1G03660	1.39E-04	0.90469766	-0.00000024	0.95159435	-0.22140527
265043_at	AT1G03900	2.10E-03	0.57276010	0.00000000	0.46913147	-0.00178003
263664_at	AT1G04250	4.03E-04	-0.39984750	-0.00000048	-0.16465855	0.56829550
261177_at	AT1G04770	1.11E-03	1.12914510	-0.00000024	0.61855700	0.20370865
261167_at	AT1G04980	4.22E-03	2.17855830	0.00000024	2.24483300	0.61192894
264570_at	AT1G05350	1.54E-03	0.87294006	0.00000000	0.87528800	0.22029924
263183_at	AT1G05570	1.53E-03	1.20271640	0.00000000	1.05133720	0.04416561
263202_at	AT1G05630	3.50E-03	1.00109360	0.00000000	0.87684274	-0.06990385
260790_at	AT1G06240	1.73E-03	-0.59802914	0.00000000	-0.48663210	-0.06850004
260789_s_at	AT1G06290 /	2.48E-03	0.84853125	0.00000000	0.44253350	-0.04366779
256050_at	AT1G07000	2.44E-03	2.01048040	0.00000000	1.77894690	-0.02440000
256042_at	AT1G07220	3.85E-03	0.49965740	0.00000024	0.62631510	-0.29318118
261078_at	AT1G07320	2.68E-03	-0.70131636	-0.00000048	-0.54464674	0.30119990
261084_at	AT1G07440	4.42E-04	-1.38886240	0.00000048	-1.36510560	0.25605917
261433_s_at	AT1G07670 /	9.99E-04	0.59288740	-0.00000048	0.52223635	0.01580334
261412_at	AT1G07890	8.08E-04	0.81300070	-0.00000048	0.97359990	0.20243263
260677_at	AT1G07910	2.87E-03	-0.23078942	0.00000000	-0.32528900	-0.20975160
260648_at	AT1G08050	1.01E-04	2.44425920	0.00000024	2.30706500	-0.30804467
261758_at	AT1G08250	2.43E-03	0.48619175	-0.00000024	0.14739728	0.12238836
261692_at	AT1G08450	9.65E-04	2.00418570	0.00000000	2.18925380	0.14712429
261695_at	AT1G08520	3.97E-03	-0.58549260	0.00000000	-0.59361270	0.01084995
264781_at	AT1G08540	1.66E-03	-0.66344595	0.00000000	-0.45069790	0.24077749
264645_at	AT1G08940	1.10E-03	1.42764120	0.00000024	1.25415400	0.04899836
264648_at	AT1G09080	2.68E-04	3.66404960	0.00000000	3.94452380	0.68302417
264657_at	AT1G09100	7.09E-04	0.92995740	0.00000000	0.88891315	0.28737593
263676_at	AT1G09340	2.15E-03	-0.49084760	0.00000000	-0.56875896	0.13103485
264708_at	AT1G09740	1.38E-03	0.99140070	0.00000000	0.87587740	0.02080464
264672_at	AT1G09750	2.75E-03	-0.74792814	-0.00000048	-0.54961777	0.11661243
264522_at	AT1G10050	8.10E-04	0.80614567	0.00000000	0.73260950	-0.13712406
264406_at	AT1G10290	1.15E-03	0.65940475	0.00000000	0.60747340	0.08022046
264434_at	AT1G10340	1.56E-03	2.36502980	0.00000024	2.31395000	-0.02264595
264458_at	AT1G10410	1.22E-03	1.06607530	-0.00000024	1.03685140	0.26519752
263237_at	AT1G10610	2.38E-03	-0.32214737	-0.00000024	-0.33020425	-0.27833652
262784_at	AT1G10760	2.02E-03	-1.07923360	0.00000048	-0.96624900	0.00316811
260465_at	AT1G10910	3.61E-03	-1.03661420	-0.00000048	-1.14904710	-0.41659070
264883_s_at	AT1G11180 //	1.23E-03	1.31301070	0.00000000	1.36095710	0.20506573
262455_at	AT1G11310	9.48E-05	0.81270410	0.00000000	0.75828170	-0.21220780
262507_at	AT1G11330	4.06E-04	1.26619050	0.00000000	1.06372360	-0.14517832
262809_at	AT1G11720	2.55E-03	-0.94149256	0.00000000	-0.93094160	0.04101658
264371_at	AT1G12090	1.48E-04	-0.61483290	0.00000048	-0.79536104	-0.04718781
260993_at	AT1G12140	3.85E-04	0.26109362	0.00000000	0.22568798	-0.15602350
261023_at	AT1G12200	4.12E-04	0.96569157	0.00000000	0.86688757	0.19918370
260966_at	AT1G12220	2.15E-03	0.33824062	0.00000024	0.28015804	-0.13855100
259534_at	AT1G12290	9.39E-05	1.73625710	-0.00000024	1.70564700	0.05043483
259512_at	AT1G12360	2.66E-03	0.81285380	0.00000000	0.67197990	-0.04601955
259531_at	AT1G12460	2.35E-03	-0.43921710	0.00000000	-0.65478900	-0.18885326
255933_at	AT1G12750	5.09E-04	-0.14999819	0.00000000	-0.22826529	-0.09628010
261196_at	AT1G12860	7.70E-04	-1.08298640	-0.00000048	-1.27289490	-0.04137945
261197_at	AT1G12900	5.86E-04	-0.37082052	0.00000000	-0.40905428	0.12289095
259385_at	AT1G13470	1.63E-03	3.39107280	0.00000000	3.38701150	0.22159982
259420_at	AT1G13900	7.98E-04	0.18923998	-0.00000048	0.26809692	0.05797625
262648_at	AT1G14030	7.93E-04	-0.72988080	-0.00000048	-0.74988030	0.25549412
262665_at	AT1G14070	8.15E-04	0.70647620	0.00000000	0.38141537	0.01809692
262612_at	AT1G14150	1.59E-03	-1.28534170	0.00000000	-1.41908450	0.05043316
262661_s_at	AT1G14230 /	1.42E-03	-0.60943220	-0.00000048	-0.79329440	0.24977780
261483_at	AT1G14270	2.06E-03	-0.36282730	0.00000048	-0.40709448	0.11260510
261488_at	AT1G14345	6.78E-04	-1.03355930	-0.00000048	-1.20146850	-0.04803133
261485_at	AT1G14360	2.70E-03	1.87320660	-0.00000048	1.71694800	0.25743198
261526_at	AT1G14370	1.37E-03	1.52531050	0.00000000	1.30695820	-0.05459833
261474_at	AT1G14540	4.95E-04	0.48597026	0.00000000	0.68178964	-0.26703262
262842_at	AT1G14720	2.95E-03	-0.90271140	-0.00000048	-0.62551310	-0.11852121
262886_at	AT1G14760	2.91E-03	-0.42787194	0.00000024	-0.23759246	-0.18410850
262847_at	AT1G14840	2.26E-03	-0.63019300	0.00000024	-0.69970465	-0.04323149
262832_s_at	AT1G14870 /	4.07E-03	2.48643640	0.00000000	2.39984460	0.25070380
262588_at	AT1G15130	7.34E-04	0.42671728	-0.00000048	0.39407873	-0.14976883
262598_at	AT1G15260	1.76E-03	-1.23437980	0.00000000	-1.46355300	-0.06957388
259489_at	AT1G15790	4.00E-05	2.03247800	0.00000000	1.87106160	-0.07712030
261788_at	AT1G15980	1.40E-03	-0.97471430	0.00000000	-1.12446170	0.02079010
261793_at	AT1G16080	1.46E-03	-0.75141525	0.00000000	-0.79370785	-0.04633379

261836_at	AT1G16090	8.35E-04	1.49299500	0.00000024	1.18765950	0.16229415
262753_at	AT1G16340	4.62E-04	-0.47847510	0.00000024	-0.46093154	0.22661924
262731_at	AT1G16420	7.68E-05	2.35423870	0.00000000	1.94711950	-0.03812552
262716_at	AT1G16470	2.26E-03	0.91914177	0.00000000	0.98971270	0.27498530
246327_at	AT1G16670	7.18E-04	1.19293790	-0.00000024	1.22050860	0.02494955
264303_s_at	AT1G16890 /.	1.97E-03	0.70074270	0.00000048	0.64893055	0.22133780
256112_at	AT1G16920	3.07E-03	-0.37240458	-0.00000048	-0.33205032	0.05280447
262481_at	AT1G17080	6.55E-04	0.69455480	0.00000048	0.62757490	-0.08975363
262519_at	AT1G17160	1.79E-04	0.17064810	-0.00000048	0.07069969	-0.13502693
262529_at	AT1G17250	1.61E-03	0.56366300	0.00000000	0.40670037	-0.29823947
261032_at	AT1G17430	5.13E-04	1.03680900	0.00000024	0.92032910	-0.22115517
260735_at	AT1G17610	3.53E-03	0.52030730	0.00000000	0.57213520	-0.43331242
256076_at	AT1G18060	3.38E-04	-0.98777580	0.00000048	-0.89577150	0.16075230
261661_at	AT1G18360	2.06E-03	-0.72037363	0.00000000	-0.70313025	0.26401280
261669_at	AT1G18490	2.29E-03	-0.46839952	-0.00000048	-0.26372170	0.03462362
255779_at	AT1G18650	3.73E-04	-1.46237830	0.00000000	-1.40530780	-0.09546614
261422_at	AT1G18730	4.33E-04	-0.63289260	0.00000000	-0.86679410	0.16316843
261428_at	AT1G18870	3.68E-03	-0.78407574	-0.00000024	-0.61133430	0.07175160
259479_at	AT1G19020	1.28E-03	2.59476100	0.00000048	2.46105050	-0.14321160
256015_at	AT1G19150	1.14E-04	-1.16162060	0.00000000	-1.19731380	0.29508066
256012_at	AT1G19250	4.16E-04	4.97618700	0.00000000	4.55764600	0.43474483
260666_at	AT1G19300	3.59E-03	1.28641560	0.00000024	1.18269110	0.14874315
260665_at	AT1G19360	6.14E-04	1.05855940	-0.00000048	0.91046620	0.07799196
260656_at	AT1G19380	1.93E-03	1.28281690	0.00000000	0.66042614	-0.51823710
261149_s_at	AT1G19550 /.	3.65E-03	-0.35571860	-0.00000048	-0.22065449	0.29462670
255782_at	AT1G19850	2.35E-03	-0.58261060	-0.00000024	-0.25922847	0.06290054
261248_at	AT1G20030	5.18E-04	-1.49843360	-0.00000048	-1.47031470	0.15540266
255941_at	AT1G20350	1.85E-03	0.57131696	0.00000000	0.72179890	-0.28532028
259518_at	AT1G20510	4.19E-03	0.90693140	0.00000000	0.73573780	-0.04284000
259564_at	AT1G20540	1.16E-04	-0.36423160	-0.00000048	-0.31727790	0.06556892
259563_s_at	AT1G20590 /	1.63E-03	-0.40645720	0.00000024	-0.25772667	0.15821695
256088_at	AT1G20810	4.05E-03	-0.68597410	0.00000000	-0.46620512	0.18563128
262796_at	AT1G20850	1.67E-04	-0.20354939	0.00000000	-0.17501593	0.08662367
261450_s_at	AT1G21110 //	6.37E-04	2.55098900	-0.00000024	2.24990490	-0.05906189
261449_at	AT1G21120	1.17E-03	2.02425100	0.00000000	1.27721550	-0.20603585
259559_at	AT1G21240	3.70E-03	3.37595990	0.00000000	3.70944880	0.50728416
259561_at	AT1G21250	1.21E-03	2.39889000	-0.00000048	2.34940770	-0.06442022
259560_at	AT1G21270	2.06E-03	1.17938610	0.00000000	1.20425130	-0.29712105
260899_at	AT1G21370	6.63E-04	1.15778590	0.00000000	1.10036330	-0.10304928
260902_at	AT1G21440	2.79E-03	-1.10171990	0.00000000	-0.48805570	0.23368597
260877_at	AT1G21500	6.56E-04	-0.95225286	0.00000000	-1.38256310	-0.24998188
260919_at	AT1G21520 /.	1.77E-05	2.16096540	0.00000000	2.53680600	0.47472978
262504_at	AT1G21750	4.18E-03	1.50474600	-0.00000048	1.78721810	0.30145216
262499_at	AT1G21770	2.96E-03	0.39440490	0.00000000	0.15906143	-0.30954456
260852_at	AT1G21900	2.14E-03	1.46460300	0.00000000	1.65353870	0.39236070
255923_at	AT1G22180	3.48E-04	1.75183490	0.00000000	1.63741210	0.12370324
255966_at	AT1G22300	3.22E-03	0.73175430	0.00000048	0.91677666	0.34751750
261934_at	AT1G22400	1.42E-04	1.95384880	-0.00000024	1.91856430	-0.04775882
261931_at	AT1G22430	2.62E-03	-0.83310103	0.00000000	-0.81343246	0.11541891
261938_at	AT1G22510	1.49E-03	0.76068780	0.00000000	0.72851800	-0.25711513
264199_at	AT1G22700	2.07E-03	-0.50491190	0.00000048	-0.18688583	0.27525330
264728_at	AT1G22850	2.73E-03	-0.55597450	0.00000048	-0.49409485	0.03611755
264901_at	AT1G23090	9.33E-04	1.28085570	0.00000048	0.79467870	0.05913019
265132_at	AT1G23830	1.50E-04	1.04070900	-0.00000024	0.69204090	-0.17925740
265189_at	AT1G23840	1.20E-05	1.07426260	0.00000000	1.16703180	-0.35559058
263032_at	AT1G23850	4.19E-03	1.30627080	0.00000000	0.84078720	-0.05746579
264866_at	AT1G24140	6.97E-04	2.44004250	0.00000024	2.40328070	-0.14439440
264867_at	AT1G24150	2.43E-03	1.83740640	0.00000024	2.05021070	0.05340838
264862_at	AT1G24330	3.79E-03	-0.69388247	0.00000000	-0.55579040	-0.24749279
265013_at	AT1G24460	1.02E-03	0.30230045	0.00000000	0.15000129	-0.07071686
247864_s_at	AT1G24807 /.	4.20E-04	1.94976280	0.00000048	1.50737620	0.07629442
255732_at	AT1G25450 /.	2.63E-03	-0.47215605	0.00000000	-0.68952847	-0.15787983
255735_at	AT1G25520	2.89E-03	0.67744875	0.00000048	0.43799830	0.12536097
255739_at	AT1G25580	1.32E-04	0.48584604	-0.00000024	0.51676180	-0.01423430
245877_at	AT1G26220	1.95E-03	-0.59362125	0.00000000	-0.58818555	0.01903868
245873_at	AT1G26260	3.65E-03	0.38272333	0.00000000	0.51321673	0.08001638
261021_at	AT1G26380	4.17E-04	2.92352680	0.00000024	2.80937430	-0.20534253
261005_at	AT1G26420	8.94E-05	2.69680240	0.00000024	2.55418660	-0.16699314
261012_at	AT1G26600	1.31E-03	-0.68044780	-0.00000024	-0.65617776	-0.31791234
261269_at	AT1G26690	3.09E-03	1.51039550	0.00000000	1.65302320	0.32648873
264984_at	AT1G27000	8.81E-04	0.55948544	0.00000000	0.53971530	-0.04856730
264989_at	AT1G27200	3.01E-03	-0.60011150	0.00000000	-0.41409135	-0.06436920
264449_at	AT1G27460	8.05E-04	-0.79283380	0.00000000	-0.84878826	-0.23909760
264442_at	AT1G27480	1.55E-03	-0.88343525	0.00000000	-0.85961150	-0.01849270
261648_at	AT1G27730	3.92E-03	2.14536100	0.00000000	1.85764980	-0.12261558
261650_at	AT1G27770	1.80E-04	1.40568400	0.00000048	1.49830150	-0.16002321
259598_at	AT1G27980	5.31E-04	0.99704120	0.00000000	1.04305600	-0.16309786
259586_at	AT1G28100	4.89E-04	-0.91970587	-0.00000024	-0.66532470	0.10961866
261443_at	AT1G28480	2.82E-03	2.56757830	-0.00000024	2.10307070	0.05356026
262749_at	AT1G28580	3.15E-03	0.43816614	-0.00000048	0.48574877	0.04655218
262748_at	AT1G28610	3.70E-03	-0.35737896	0.00000024	-0.09425879	0.23965430
262744_at	AT1G28680	7.92E-04	0.69915056	0.00000000	0.73160530	0.18185878
260842_at	AT1G29150	5.62E-05	0.63517237	0.00000000	0.51586866	0.12254763
260897_at	AT1G29330	5.06E-04	0.73896930	0.00000024	0.68958116	0.05923867
259768_at	AT1G29390	1.17E-04	-0.65433025	0.00000000	-0.72239970	-0.17895222
259789_at	AT1G29395	8.78E-04	-1.40290500	0.00000048	-1.25099130	-0.02249670
259773_at	AT1G29500	7.81E-04	-1.47028400	0.00000048	-1.96513750	-0.23004580
259775_at	AT1G29530	4.29E-03	-0.66968346	0.00000000	-0.72179320	-0.02353001
259786_at	AT1G29660	3.38E-04	-0.89009190	0.00000048	-0.53319120	0.19256640

260029_at	AT1G30000	1.36E-03	0.57278490	0.00000000	0.51655483	0.02756596
245775_at	AT1G30270	2.30E-03	1.02429650	0.00000000	1.19141150	0.19282079
261805_at	AT1G30540	1.10E-03	0.34834100	0.00000000	0.35530043	0.02287674
263222_at	AT1G30640	3.12E-04	1.22655100	0.00000000	0.81835127	-0.19743657
265161_at	AT1G30900	6.07E-04	2.47333430	0.00000000	2.46402600	0.12165570
263704_at	AT1G31130	2.39E-03	1.19298460	-0.00000048	1.26360320	0.29151154
263705_at	AT1G31190	3.72E-03	-0.40806484	-0.00000048	-0.34173012	0.07972240
256483_at	AT1G31410	7.43E-04	-0.60165260	0.00000000	-0.65212727	-0.02298689
256482_at	AT1G31420	2.05E-03	-0.81018066	-0.00000048	-0.69946957	-0.30993605
256489_at	AT1G31550	2.50E-03	1.09871010	0.00000048	0.99555970	-0.01832581
246576_at	AT1G31650	1.14E-03	-0.62299440	0.00000000	-0.49547005	0.08706808
246268_at	AT1G31800	3.06E-03	-0.88937044	-0.00000048	-0.86656570	-0.07150841
255719_at	AT1G32080	3.20E-04	-0.73564960	0.00000000	-0.79251003	0.16542244
245789_at	AT1G32090	7.75E-05	-1.10709190	-0.00000048	-1.08692500	0.14580154
245788_at	AT1G32120 /	1.54E-03	0.54775570	0.00000000	0.38080597	-0.13574934
245790_at	AT1G32200	7.19E-04	-0.75136614	0.00000000	-0.71537830	0.17471313
245793_at	AT1G32220	6.63E-04	-0.89269210	0.00000000	-1.02218910	-0.00182152
260706_at	AT1G32350	2.25E-03	1.19522500	-0.00000024	1.19090100	-0.45246744
260704_at	AT1G32470	8.86E-04	-0.85049390	0.00000048	-0.95661590	0.06563473
256468_at	AT1G32550	7.08E-04	-0.49940680	0.00000000	-0.63259890	-0.00420523
261697_at	AT1G32610	9.22E-04	-0.24346256	0.00000000	-0.42184973	-0.24586200
261242_at	AT1G32960	2.53E-03	3.28820850	0.00000024	2.64470620	0.06755424
261216_at	AT1G33030	3.40E-03	1.99412540	-0.00000024	1.41355940	-0.42831087
261593_at	AT1G33170	8.61E-04	-0.65925980	-0.00000024	-0.53098010	0.19916654
256534_at	AT1G33270	3.90E-03	0.26514673	0.00000000	0.33545303	0.10437822
256530_at	AT1G33290	2.42E-03	-0.39462447	0.00000000	-0.50290036	0.16025066
245765_at	AT1G33600	1.29E-04	0.49656677	0.00000000	0.16572046	0.13743734
260119_at	AT1G33930	2.20E-03	-0.38730693	0.00000000	-0.38743590	0.15947318
260116_at	AT1G33960	1.73E-03	3.92656560	0.00000000	4.24172260	0.64904857
262542_at	AT1G34180	3.57E-04	0.63696000	0.00000024	0.79820490	-0.43488503
262514_at	AT1G34190	5.62E-04	0.64164686	0.00000048	0.56707860	-0.02940464
262563_at	AT1G34210	3.79E-03	-0.57079650	-0.00000024	-0.69008446	0.08173513
261161_at	AT1G34420	2.55E-05	1.28149370	0.00000000	1.25754400	-0.10890031
262408_at	AT1G34750	1.87E-03	1.47012470	0.00000024	1.70152140	-0.00833178
255079_s_at	AT1G35160 /	4.00E-05	0.31416893	0.00000048	0.35349370	-0.06394005
245755_at	AT1G35210	2.12E-04	1.43143370	0.00000000	0.99692010	-0.14479947
262029_at	AT1G35680	2.13E-03	-0.33449936	0.00000000	-0.25612210	0.19744825
261339_at	AT1G35710	1.42E-03	2.85873080	0.00000024	2.93841930	0.24042296
261286_at	AT1G35780	4.29E-03	-0.29766417	0.00000048	-0.21270657	0.23952007
256461_s_at	AT1G36280 /	3.29E-03	-0.29722548	0.00000000	0.01897001	0.72219515
262721_at	AT1G43560	4.15E-03	-0.70857096	0.00000000	-0.76346684	-0.09721136
259507_at	AT1G43910	1.05E-03	3.09469220	-0.00000024	3.22566750	-0.04941416
245738_at	AT1G44130	2.89E-03	1.35745550	0.00000000	1.44274140	-0.11916900
261338_at	AT1G44920	1.94E-03	-0.59172297	0.00000000	-0.52010010	0.17137480
260943_at	AT1G45145	1.30E-03	2.36096620	0.00000048	2.17819070	0.17256021
245806_at	AT1G45474	1.31E-04	-0.81408930	0.00000000	-0.99632500	-0.06901217
260503_at	AT1G47250	3.88E-03	0.95907927	0.00000000	1.04833320	0.40050507
262434_at	AT1G47670	2.16E-03	-0.67075634	0.00000000	-0.52885675	-0.01277065
260730_at	AT1G48030	2.47E-03	-0.56376220	-0.00000048	-0.32073545	0.27311277
260728_at	AT1G48210	4.23E-03	1.47393850	0.00000000	1.53799100	0.23299909
262245_at	AT1G48240	5.36E-04	-0.79487850	0.00000024	-0.76201034	0.01610375
262237_at	AT1G48320	8.18E-05	1.54918100	0.00000048	1.56217000	-0.16842294
261309_at	AT1G48598 /	2.23E-04	-0.93002367	0.00000000	-0.82595350	0.22468328
256145_at	AT1G48750	4.36E-04	-0.64675546	0.00000000	-0.51101875	-0.07791138
256165_at	AT1G48780	4.17E-03	-0.66864610	0.00000000	-0.54925513	-0.33399320
260754_at	AT1G49000	1.08E-03	1.46638750	0.00000000	1.16459180	0.09082770
260772_at	AT1G49050	5.31E-04	1.39660550	0.00000048	1.49473670	-0.28905392
262397_at	AT1G49380	1.83E-03	-0.67176200	0.00000000	-0.70698640	0.16200876
262393_at	AT1G49490	4.32E-04	-0.60058856	0.00000000	-0.53345656	-0.14821911
261607_at	AT1G49660	7.35E-04	-0.38685298	0.00000048	-0.26902390	0.27271270
261638_at	AT1G49975	2.93E-03	-1.01912950	0.00000000	-1.20223740	0.11227274
261866_at	AT1G50420	1.46E-03	0.79882310	0.00000024	0.60997580	0.02392769
246630_at	AT1G50730	4.24E-03	-1.14285800	0.00000000	-0.98125460	-0.23792171
246631_at	AT1G50740	4.80E-04	1.32536510	0.00000000	1.21347710	-0.15276718
245749_at	AT1G51090	3.33E-03	-1.77287270	0.00000048	-1.52333380	-0.10576725
245745_at	AT1G51110	1.33E-03	-0.93052100	0.00000000	-1.07699680	-0.07347632
260511_at	AT1G51570	5.78E-04	-0.62065220	0.00000048	-0.66320515	-0.06734228
256177_at	AT1G51620	6.50E-04	1.03277870	0.00000000	0.84470130	0.15076923
256183_at	AT1G51660	2.56E-03	1.72209400	-0.00000024	1.70665550	0.13652754
256179_at	AT1G51710	1.19E-03	0.59298944	0.00000000	0.83297350	0.21291828
256178_s_at	AT1G51760 /	8.42E-04	2.33982000	0.00000000	1.83271700	0.01950693
256170_at	AT1G51790	2.38E-03	1.27967830	0.00000024	0.99908304	-0.21743608
256169_at	AT1G51800	3.67E-04	1.81362630	0.00000000	1.64083770	-0.09047985
246366_at	AT1G51850	6.40E-04	1.47358630	-0.00000024	0.99245240	0.02201629
246373_at	AT1G51860	1.99E-03	0.78920960	-0.00000024	0.94295120	-0.00957370
246368_at	AT1G51890	2.23E-03	2.80433030	0.00000000	2.50484320	-0.08948827
246370_at	AT1G51920	1.90E-03	1.35976170	0.00000000	1.19710060	-0.44890690
259841_at	AT1G52200	4.10E-03	1.96426530	0.00000000	1.39427660	-0.00843287
262155_at	AT1G52420	5.93E-05	0.67621470	0.00000048	0.76690817	0.08870840
262161_at	AT1G52600	6.31E-04	0.97546720	0.00000048	1.03853700	0.14683533
261317_at	AT1G53030	6.20E-04	1.20619230	0.00000000	0.83107040	-0.09405541
260644_at	AT1G53290	1.93E-03	-0.28653050	0.00000048	-0.52071047	-0.18053246
260989_at	AT1G53450	4.13E-03	-0.86172867	-0.00000048	-0.59471416	-0.15913868
260982_at	AT1G53520	4.18E-04	-0.82540080	0.00000048	-1.06626610	-0.03249836
263156_at	AT1G54030	7.51E-05	0.73811910	0.00000000	0.71615744	0.17582178
263174_at	AT1G54040	3.49E-03	-0.73627660	0.00000000	-0.66492414	0.15176582
263162_at	AT1G54150	4.23E-03	0.70514345	0.00000048	0.37690640	-0.00299692
262963_at	AT1G54220	2.74E-03	-0.39883566	0.00000024	-0.19735622	0.09157562
262960_at	AT1G54320	1.14E-03	1.09233950	-0.00000048	0.92988350	0.04360771

262954_at	AT1G54500	2.26E-03	-0.77517460	-0.00000048	-0.81995250	-0.05232716
264186_at	AT1G54570	2.52E-03	-0.47063303	0.00000024	-0.57498455	0.10883904
259658_at	AT1G55370	1.36E-04	-1.31744530	-0.00000048	-1.56734160	-0.06578922
260601_at	AT1G55910	7.44E-04	1.34291550	-0.00000048	1.37172370	-0.14393711
259604_at	AT1G56450	9.26E-04	0.84706210	0.00000000	0.86990070	0.26299047
259603_at	AT1G56500	6.01E-05	-0.77570630	0.00000048	-0.91940403	-0.14044619
246401_at	AT1G57560	5.07E-04	2.28806780	0.00000024	1.81969070	-0.19653988
246405_at	AT1G57630	2.67E-04	3.72304200	0.00000024	3.58600190	0.15952086
246406_at	AT1G57650	1.22E-03	1.06230620	0.00000024	0.93350863	-0.11985493
256018_at	AT1G58300	3.22E-03	1.01745630	0.00000000	1.02940750	0.12602878
262910_at	AT1G59710	3.36E-03	1.00033810	-0.00000024	1.17633680	-0.10792828
262907_at	AT1G59720	2.96E-04	-0.87938550	0.00000000	-0.79344010	-0.11438966
262897_at	AT1G59840	1.05E-03	-0.91628313	-0.00000024	-0.90918064	0.21367216
262899_at	AT1G59870	4.24E-03	0.68768406	0.00000048	0.56237745	-0.07300949
264219_at	AT1G60420	1.64E-03	1.36233570	0.00000024	1.21456380	0.31816435
264244_at	AT1G60440	3.47E-03	0.46741915	-0.00000048	0.18211603	-0.14741135
264920_at	AT1G60550	9.90E-04	-0.55497410	0.00000000	-0.85124160	0.12092686
264963_at	AT1G60600	1.57E-03	-0.77423190	0.00000048	-0.62161300	0.03673267
264911_at	AT1G60690	6.88E-04	-0.80781436	0.00000000	-0.66712500	0.11108422
264929_at	AT1G60730	1.24E-03	1.04340700	0.00000000	1.30985620	0.20668983
264923_s_at	AT1G60740 /	3.22E-03	1.50947950	-0.00000024	1.19964340	0.08361149
264932_at	AT1G61240	1.41E-03	-0.42515993	-0.00000024	-0.44862770	-0.03677392
264879_at	AT1G61260	3.11E-03	0.30923963	0.00000000	0.60226630	-0.25696683
264757_at	AT1G61360	8.45E-05	1.78759860	0.00000000	1.41456750	-0.03998590
264756_at	AT1G61370	1.76E-03	1.27254840	0.00000000	0.85578084	-0.15835500
264766_at	AT1G61420	7.69E-05	1.19053790	-0.00000024	1.16523200	-0.11879301
265008_at	AT1G61560	3.02E-04	1.57004190	0.00000024	1.15250280	-0.13106155
264428_at	AT1G61790	2.41E-04	0.57428600	0.00000048	0.58984330	0.03677034
264400_at	AT1G61800	3.66E-03	2.95156240	0.00000000	3.19314200	0.23065805
264307_at	AT1G61900	1.38E-03	-0.73464870	0.00000048	-0.89186860	-0.19120121
264744_at	AT1G62050	3.80E-03	-0.48229670	0.00000000	-0.41835856	-0.04959631
264737_at	AT1G62210	2.86E-03	-0.38981628	0.00000000	-0.14718151	-0.18146992
264746_at	AT1G62300	1.82E-04	1.47227810	-0.00000024	1.39421610	0.08762407
260109_at	AT1G63260	1.23E-03	-0.74206970	0.00000048	-1.00990180	-0.28373766
261545_at	AT1G63530	2.36E-04	0.15662408	0.00000024	0.16509962	-0.20457196
260243_at	AT1G63720	3.87E-03	1.27470870	0.00000000	1.29778670	0.02034760
260314_at	AT1G63830	8.14E-04	0.24340630	0.00000000	0.28262330	-0.06837893
260327_at	AT1G63840	1.73E-04	2.23022560	-0.00000048	1.78256510	-0.15232325
262344_at	AT1G64060	8.69E-04	0.36155438	0.00000000	0.59410360	0.20766354
259764_at	AT1G64280	1.12E-03	0.82640123	0.00000000	0.68731120	-0.10218239
259738_at	AT1G64355	7.88E-04	-1.33098220	0.00000048	-1.23469540	-0.04231501
261952_at	AT1G64430	3.34E-03	-0.48066378	0.00000048	-0.56314680	-0.13966560
262003_at	AT1G64460	2.82E-03	0.53197980	0.00000000	0.35805345	0.05156779
261973_at	AT1G64610	4.25E-03	1.57871440	0.00000000	1.44885350	0.22607899
262878_at	AT1G64770	9.26E-04	-1.30558970	0.00000000	-1.33009200	-0.17925310
264161_at	AT1G65420	2.59E-03	-0.49148655	0.00000000	-0.52301025	-0.09779501
262932_at	AT1G65820	2.86E-05	1.14283510	0.00000000	1.05207540	0.05227470
261917_at	AT1G65920	4.83E-04	-0.67630460	-0.00000024	-0.56294820	-0.18248415
256526_at	AT1G66090	9.58E-04	1.63388200	0.00000000	1.62078240	-0.34875345
256522_at	AT1G66160	2.56E-04	1.97713730	0.00000000	1.53918600	0.05765128
256376_s_at	AT1G66690 /	2.91E-03	1.96472260	0.00000000	1.50029800	-0.19895756
256366_at	AT1G66880	6.20E-04	2.97934060	0.00000000	3.10261200	0.67021490
245760_s_at	AT1G66910 /	2.45E-03	0.91048527	-0.00000024	0.97288750	0.22507739
255912_at	AT1G66960	2.54E-03	0.64823700	-0.00000024	0.85921670	-0.10190725
255851_at	AT1G67040	2.53E-03	-0.67328095	-0.00000024	-0.60131025	0.19471312
264998_at	AT1G67330	3.80E-04	0.92804720	0.00000000	0.67626380	-0.00524020
264224_at	AT1G67440	3.84E-03	-0.36643076	0.00000000	-0.36375737	0.21171618
264232_at	AT1G67470	3.51E-03	0.46791792	0.00000000	0.56557417	-0.05610132
264223_s_at	AT1G67520 /	4.11E-04	2.02816410	0.00000000	1.74394060	-0.00852966
245190_at	AT1G67690	4.22E-03	-0.87230350	0.00000000	-0.79155517	-0.40806770
245198_at	AT1G67700	3.48E-05	-0.79184770	0.00000000	-0.78212786	0.07138968
245195_at	AT1G67740	2.65E-03	-0.35300112	0.00000000	-0.40545988	0.10726929
245197_at	AT1G67800	6.85E-06	1.36755080	0.00000000	1.61282830	-0.06419778
245193_at	AT1G67810	2.17E-04	2.10628560	0.00000024	1.90996310	-0.12061501
245200_at	AT1G67850	1.40E-03	1.39787630	0.00000000	1.47631450	0.36724472
259992_at	AT1G67970	1.82E-03	0.83448650	0.00000000	0.86479710	-0.19221592
259999_at	AT1G68080	3.28E-04	-0.51029444	0.00000000	-0.35448456	0.16419220
260442_at	AT1G68220	2.67E-03	-0.57357025	-0.00000048	-0.39148092	0.18776321
260444_at	AT1G68300	1.01E-03	1.31816480	0.00000000	1.20743750	0.12798548
260260_at	AT1G68540	6.36E-04	-0.80994510	0.00000024	-0.78126050	0.23601079
262275_at	AT1G68710	1.79E-03	1.06807780	0.00000000	1.28894520	0.26386046
257516_at	AT1G69040	1.57E-03	-0.64146520	0.00000000	-0.24522400	0.56729270
260345_at	AT1G69270	1.33E-03	1.32703450	0.00000024	1.29048160	0.01872158
256300_at	AT1G69490	4.03E-04	2.96433260	0.00000000	1.98309420	-0.40563320
256304_at	AT1G69523	3.67E-04	-1.00637700	0.00000024	-1.37189580	0.06616783
260371_at	AT1G69690	2.92E-03	-0.70556164	0.00000048	-0.45041418	0.06341791
260368_at	AT1G69700	4.03E-03	-0.97639180	-0.00000048	-0.96294045	0.01423502
264703_at	AT1G69960	9.99E-04	0.63795185	0.00000000	0.42534304	-0.00811863
264717_at	AT1G70140	2.15E-04	0.71430945	0.00000024	0.64157796	-0.30225540
264716_at	AT1G70170	3.63E-03	0.38195038	0.00000024	0.49754667	-0.13745880
264313_at	AT1G70410	3.35E-03	-0.53493834	0.00000000	-0.62355950	-0.13430500
260305_at	AT1G70490	2.51E-03	0.48636818	0.00000000	0.53255844	0.18400002
260363_at	AT1G70550	2.11E-03	-0.46690083	0.00000000	-0.53308250	0.17876077
260179_at	AT1G70690	7.83E-04	1.84368130	0.00000024	1.95874190	0.08192015
259749_at	AT1G71100	1.56E-03	1.54181220	0.00000024	1.44596240	0.48512530
259952_at	AT1G71400	1.11E-03	0.48758388	0.00000024	0.34791207	-0.26654744
261518_at	AT1G71695	1.64E-03	-0.68632700	-0.00000048	-0.64659166	-0.04463291
261520_at	AT1G71820	1.05E-03	0.24695969	0.00000000	0.27097130	-0.06222773
260171_at	AT1G71910	6.88E-04	0.32910180	0.00000000	0.23578000	-0.14247632

260174_at	AT1G71940	2.36E-03	0.44978880	0.00000000	0.42080380	-0.00944138
259801_at	AT1G72230	4.57E-04	-0.50323940	0.00000000	-0.34844208	0.04853225
259850_at	AT1G72240	1.60E-03	0.99848200	0.00000000	0.92181040	-0.12825203
259852_at	AT1G72280	4.22E-04	2.51826480	0.00000000	2.20802300	0.08392334
260454_at	AT1G72310	2.25E-03	-0.54650450	0.00000000	-0.37528872	0.05147314
260399_at	AT1G72520	8.46E-04	2.68947740	0.00000024	2.11613270	-0.19066286
259921_at	AT1G72540	1.09E-03	1.06962440	0.00000000	1.28283670	-0.18649721
259912_at	AT1G72670	1.49E-03	-0.45210670	0.00000000	-0.11045885	0.02471542
259910_at	AT1G72700	8.60E-04	0.76042270	0.00000000	1.06934500	0.15908432
262381_at	AT1G72900	2.86E-03	2.57534600	0.00000000	2.16924330	0.02141857
262382_at	AT1G72920	9.94E-05	1.92275710	0.00000024	1.43702770	-0.11766148
262383_at	AT1G72940	3.64E-03	1.27255580	0.00000000	1.21585700	0.02856994
262377_at	AT1G73110	1.14E-03	-0.72030690	0.00000000	-0.71688750	0.13675451
260046_at	AT1G73805	7.42E-04	2.14162540	0.00000000	2.23539780	-0.22799087
260388_at	AT1G74070	1.33E-03	-1.02870420	0.00000000	-0.91448020	0.21095371
260394_at	AT1G74080	3.50E-03	0.55817030	0.00000024	0.64622283	-0.00954485
260387_at	AT1G74100	3.39E-03	1.45833020	0.00000000	1.31511020	0.29172993
260249_s_at	AT1G74280 /	1.18E-03	0.90910720	-0.00000024	0.68143535	-0.00880075
260248_at	AT1G74310	2.62E-03	0.50405170	0.00000024	0.56727030	0.08191133
260239_at	AT1G74360	5.10E-04	2.13909820	0.00000000	2.12361720	-0.05412984
260211_at	AT1G74440	2.31E-03	0.91456030	0.00000000	0.78639317	-0.49239420
260225_at	AT1G74590	3.60E-03	2.55197760	0.00000000	2.70040560	0.27036572
262177_at	AT1G74710	2.50E-04	2.83771420	0.00000000	3.00807100	0.48639630
262175_at	AT1G74880	3.53E-03	-0.97000310	-0.00000048	-1.24330850	0.17401266
259925_at	AT1G75040	1.91E-03	3.09392070	0.00000000	3.39781480	0.29143190
256451_s_at	AT1G75170 /	3.70E-04	1.56737950	-0.00000024	1.56409220	-0.01867962
262980_at	AT1G75680	8.44E-04	-0.60664750	0.00000048	-0.63134670	-0.12658548
262970_at	AT1G75690	2.47E-04	-0.85268500	0.00000048	-1.06807570	0.01527309
262728_at	AT1G75820	1.96E-03	-0.82946205	0.00000048	-0.91203547	-0.34371138
262682_at	AT1G75900	1.92E-03	-1.05277060	0.00000000	-0.70768640	0.17688799
262698_at	AT1G75960	2.24E-05	-1.46682330	-0.00000024	-1.72141620	-0.43515730
262671_at	AT1G76040	9.95E-05	2.19973200	0.00000000	2.25931260	0.04736352
261754_at	AT1G76130	3.34E-04	0.59775780	0.00000000	0.43762040	0.17893219
259884_at	AT1G76390	2.79E-03	0.40342950	0.00000000	0.43551922	-0.17613220
264951_at	AT1G76970	8.94E-04	1.32475230	-0.00000024	1.34652570	-0.09886551
264954_at	AT1G77060	1.12E-03	-1.05426410	0.00000000	-1.27142600	-0.14069033
264959_at	AT1G77090	2.18E-04	-0.55028486	0.00000000	-0.57625914	0.27937222
264485_at	AT1G77220	3.07E-04	0.03869033	0.00000000	0.11717057	-0.07665086
246413_at	AT1G77310	4.10E-03	-0.37783290	-0.00000024	-0.39315748	-0.14205956
246384_at	AT1G77370	3.21E-03	0.83160450	0.00000048	0.74117374	-0.16454506
259708_at	AT1G77420	1.25E-03	0.57362270	0.00000048	0.40659142	-0.16520715
259707_at	AT1G77490	2.21E-03	-0.79000616	0.00000000	-1.00410510	-0.12753487
259734_at	AT1G77500	2.19E-03	0.69317530	-0.00000024	0.78780770	0.05613446
262137_at	AT1G77920	2.41E-03	0.52878857	-0.00000048	0.62753680	0.18967438
260081_at	AT1G78170	1.25E-03	-1.15240430	0.00000000	-1.02883340	0.07789135
260082_at	AT1G78180	9.02E-04	-0.97476390	0.00000000	-0.93447113	-0.09442997
260746_at	AT1G78380	1.87E-03	0.57693150	0.00000000	0.77066610	0.36776210
260804_at	AT1G78410	2.76E-03	3.75307420	0.00000000	3.49554970	0.21728158
263133_at	AT1G78450	2.32E-03	-0.65394950	0.00000000	-0.86899710	0.10668421
263134_at	AT1G78570	2.00E-03	1.30152650	0.00000000	1.36807160	0.35949040
264250_at	AT1G78680	3.08E-03	-0.70068360	0.00000048	-0.37610197	0.00330591
264291_at	AT1G78800	3.58E-03	0.23292279	0.00000000	0.24320960	-0.26585388
264279_s_at	AT1G78820 /	3.10E-03	0.75984480	0.00000048	0.29373120	-0.32515526
264096_at	AT1G78995	7.54E-04	-1.12295960	-0.00000048	-0.91722630	0.09558821
264120_at	AT1G79340	1.67E-03	0.54304314	0.00000000	0.56453943	0.04860973
262942_at	AT1G79450	9.78E-05	1.54991840	0.00000000	1.68962550	0.15393877
262945_at	AT1G79510	3.32E-03	-0.79286290	0.00000000	-0.68454840	0.06518364
261394_at	AT1G79680	2.95E-03	1.65140630	0.00000024	1.33277940	-0.23239875
261346_at	AT1G79720	1.90E-03	-0.87547064	0.00000048	-0.83858250	-0.30050707
261351_at	AT1G79790	3.49E-03	-0.47985554	0.00000000	-0.74422790	-0.18524313
262059_at	AT1G80030	1.97E-03	-0.56521080	0.00000048	-0.55140830	0.25734997
262050_at	AT1G80130	3.85E-03	1.51773120	0.00000000	1.58313540	0.16793180
261892_at	AT1G80840	1.11E-03	2.76900100	0.00000000	2.44118600	-0.11929774
265737_at	AT2G01180	1.95E-03	0.35514832	0.00000048	0.18954706	-0.50607440
266342_at	AT2G01540	2.75E-04	1.21303370	0.00000000	1.10177560	-0.08065867
266329_at	AT2G01590	3.75E-03	-1.01878400	0.00000048	-1.13128570	-0.05645800
265868_at	AT2G01650	1.07E-04	1.29983380	-0.00000048	1.24098630	0.13452768
265871_at	AT2G01680	2.33E-04	0.62321710	0.00000024	0.56160640	0.05643129
265869_at	AT2G01760	1.10E-03	-1.02056410	0.00000000	-0.84482620	-0.45520140
263597_at	AT2G01870	1.40E-03	-1.36503600	0.00000000	-1.56725500	-0.28603410
266120_at	AT2G02070	3.01E-04	-0.68216990	0.00000000	-0.62292480	0.07126236
266181_at	AT2G02390	9.82E-05	1.41550020	0.00000048	1.22162870	0.05192757
267481_at	AT2G02780	2.67E-06	-0.85758376	0.00000000	-1.01413150	-0.11254668
266746_s_at	AT2G02930 /	4.14E-03	2.50702860	0.00000000	2.52219580	0.33208704
266709_at	AT2G03120	3.06E-04	1.28583430	0.00000000	1.32716560	0.42057276
265719_at	AT2G03500	1.45E-03	-0.24334383	0.00000000	-0.32437992	-0.15962076
265717_at	AT2G03510	3.26E-03	1.02939800	-0.00000048	1.29807000	0.20071602
264041_at	AT2G03710	2.43E-03	-0.74611760	0.00000000	-0.71657515	0.01884770
264037_at	AT2G03750	1.74E-03	-0.74660444	0.00000048	-0.62719440	0.43664837
263322_at	AT2G04270	4.12E-03	-0.63623047	0.00000000	-0.38081170	0.49887180
263807_at	AT2G04400	1.12E-03	2.21139530	0.00000000	1.98761990	0.00871325
263854_at	AT2G04430	3.27E-04	2.16769270	0.00000000	2.44339560	-0.08274341
263852_at	AT2G04450	2.41E-03	2.84825700	-0.00000024	3.20522550	0.23471928
263054_at	AT2G04620	1.23E-03	0.54284050	0.00000024	0.46887255	-0.12421846
263631_at	AT2G04900	2.17E-03	0.70424510	-0.00000048	0.39650726	-0.30271340
263048_s_at	AT2G05310 /	9.69E-04	-0.88003683	0.00000000	-1.00273180	-0.23616982
263105_at	AT2G05320	8.19E-04	1.07952670	0.00000000	0.92462610	0.20791411
266037_at	AT2G05940	1.26E-03	1.75553800	-0.00000048	1.83786770	0.26719760
265373_at	AT2G06510	4.28E-03	0.36290264	0.00000000	0.11840677	-0.21533728

265375_at	AT2G06530	7.17E-04	0.64227150	0.00000000	0.68885565	0.03365278
263053_at	AT2G13440	1.94E-03	-0.60784390	-0.00000024	-0.62863540	-0.06037903
263726_at	AT2G13610	3.47E-03	-0.91592026	-0.00000048	-1.13632060	-0.38624907
263722_at	AT2G13650	2.34E-03	0.68482830	0.00000048	0.40744400	0.04041910
264107_s_at	AT2G13790 /	4.31E-04	2.22058200	0.00000000	2.38446040	0.36447716
265658_at	AT2G13810	4.77E-04	2.39028120	-0.00000024	2.39300970	-0.07655168
267106_s_at	AT2G14720 /	1.94E-03	0.52101610	0.00000048	0.68979070	0.01229334
265894_at	AT2G15050	1.87E-03	-0.52368260	-0.00000048	-0.56484413	0.07293892
263297_at	AT2G15310	3.84E-03	0.65062260	0.00000000	0.62882495	0.18666506
263565_at	AT2G15390	1.14E-04	1.80244540	0.00000000	1.54717640	-0.22025204
265494_at	AT2G15680	7.07E-04	-0.54489850	0.00000024	-0.44423700	-0.15987635
265480_at	AT2G15970	2.71E-04	-0.65441180	0.00000000	-0.53538230	0.02488422
263606_at	AT2G16280	1.29E-03	-0.97702410	0.00000000	-1.03869010	-0.21478844
263241_at	AT2G16500	3.73E-03	1.04249000	0.00000000	0.56491137	0.07700348
265356_at	AT2G16595	3.57E-03	0.70807720	0.00000000	0.69365050	-0.04974723
266536_at	AT2G16900	3.19E-03	1.56817960	0.00000024	1.59495780	0.37087130
263574_at	AT2G16990	3.13E-03	-1.28088690	0.00000000	-1.16355990	0.03945541
263550_at	AT2G17033	1.91E-03	-0.55996180	0.00000000	-0.45194864	-0.11328650
263584_at	AT2G17040	2.05E-03	1.98429300	0.00000000	2.15926030	-0.12737179
263419_at	AT2G17220	4.10E-06	0.92980003	0.00000048	1.07041500	-0.11991453
264909_at	AT2G17300	2.49E-03	-0.78751490	-0.00000024	-0.80808780	0.27059865
264904_s_at	AT2G17420 /	2.90E-03	0.83341646	0.00000000	0.76483200	0.20510006
264589_at	AT2G17650	3.82E-03	-0.75858974	0.00000024	-0.78292800	0.04679346
264592_at	AT2G17720	1.22E-03	1.62509160	-0.00000048	1.48846820	0.08923245
264619_at	AT2G17760	3.78E-03	0.99768543	0.00000000	0.90596580	0.01714039
264622_at	AT2G17790	3.25E-04	1.17355870	-0.00000024	1.16954900	0.41554832
266070_at	AT2G18660	1.87E-03	3.66701560	0.00000000	3.87967250	0.31455708
266071_at	AT2G18670 /	9.59E-06	1.40675400	0.00000000	1.25397900	-0.35727692
266017_at	AT2G18690	1.79E-03	2.38329600	0.00000000	2.35693600	0.01873732
266018_at	AT2G18710	2.61E-03	-0.71208000	0.00000048	-0.78129100	0.02352905
267490_at	AT2G19130	6.93E-05	0.75045300	0.00000000	0.66509485	-0.33270310
267436_at	AT2G19190	3.10E-03	2.56040380	0.00000000	2.43253000	0.44759417
266685_at	AT2G19710	1.60E-03	0.75683640	0.00000000	0.55880547	-0.38346362
266682_at	AT2G19780	7.09E-04	-0.46185230	0.00000000	-0.40514588	0.15416980
265597_at	AT2G20142	4.08E-04	2.10571380	0.00000000	2.17637090	0.38654995
265283_at	AT2G20370	3.26E-03	0.82262990	0.00000000	1.05042790	0.29634285
265415_at	AT2G20890	2.76E-04	-0.46741772	-0.00000048	-0.49737787	0.13102722
265385_at	AT2G20900	8.93E-04	0.32739258	0.00000000	0.28903675	-0.25454140
264014_at	AT2G21210	1.50E-03	-1.55826900	0.00000048	-1.86419300	-0.09607506
263761_at	AT2G21330	7.08E-04	-0.65956545	0.00000000	-0.56756306	0.28030014
263749_at	AT2G21520	7.05E-04	0.65039350	0.00000000	0.52958345	-0.06250286
263765_at	AT2G21540	1.21E-03	-0.60618780	0.00000000	-0.62094283	-0.07208586
263517_at	AT2G21620	1.86E-03	0.84250784	0.00000048	0.78705690	0.19451046
257432_at	AT2G21850	2.64E-03	0.57147360	0.00000000	0.54791236	-0.16287279
263893_at	AT2G21900	2.22E-03	0.83651160	0.00000000	0.44944048	-0.47190213
263880_at	AT2G21960	2.11E-04	-0.59954740	-0.00000048	-0.67816450	0.03783894
263869_at	AT2G22000	2.52E-03	-0.17532897	-0.00000024	-0.24771547	-0.06849337
263429_at	AT2G22250	1.62E-03	0.36104584	0.00000000	0.27766370	0.19129562
264044_at	AT2G22480	1.08E-03	0.78944063	-0.00000048	0.69583225	-0.05864000
264000_at	AT2G22500	3.26E-03	1.92303560	0.00000000	1.93790960	0.19559431
266825_at	AT2G22890	2.93E-03	-0.46838188	0.00000024	-0.63395930	0.03928495
267288_at	AT2G23680	2.14E-03	1.90658570	0.00000000	1.86229040	0.02071619
267298_at	AT2G23760	2.35E-03	-0.70110800	0.00000000	-0.91075230	-0.31861448
266001_at	AT2G24150	3.94E-03	-0.47286034	0.00000000	-0.33150816	0.07568693
266000_at	AT2G24180	1.08E-04	1.20649810	-0.00000048	1.09506270	-0.09357452
263788_at	AT2G24580	3.67E-03	-0.42639350	0.00000024	-0.48488760	0.40241623
263800_at	AT2G24600	1.29E-03	1.33660130	0.00000024	1.01626780	-0.50013730
263539_at	AT2G24850	7.35E-04	4.28734500	0.00000048	3.87068460	0.33055067
264382_at	AT2G25110	2.34E-03	1.32664970	0.00000000	1.47829390	0.27491808
264380_at	AT2G25190	2.02E-03	0.35629750	0.00000000	0.41540194	-0.14886642
265617_at	AT2G25520	5.35E-04	0.79153967	0.00000000	0.94505215	0.08190298
257365_x_at	AT2G26020	1.67E-03	2.82654290	0.00000000	2.10409700	-0.89009380
266845_at	AT2G26110	1.95E-03	-0.36684465	0.00000024	-0.34771228	-0.13418150
267380_at	AT2G26170	2.27E-03	1.11097150	0.00000000	1.03611330	0.07257652
267381_at	AT2G26190	2.67E-04	0.88864040	-0.00000048	0.84370613	-0.10253191
267374_at	AT2G26230	1.18E-03	0.60957910	-0.00000048	0.83876660	0.19461107
267400_at	AT2G26240	1.48E-03	0.56347610	0.00000000	0.42183400	-0.16890192
267377_at	AT2G26250	5.58E-04	-0.62299395	0.00000048	-0.74158480	-0.03257465
245042_at	AT2G26540	2.84E-03	-0.55187580	-0.00000048	-0.59593487	-0.01285267
245027_at	AT2G26550	1.06E-03	-0.95745254	0.00000000	-0.65177800	0.17985344
267610_at	AT2G26650	3.09E-04	0.51963090	-0.00000024	0.22740674	-0.06369567
265628_at	AT2G27290	3.43E-03	-0.73850536	-0.00000048	-0.96162940	-0.37732935
265620_at	AT2G27310	5.69E-04	1.02007560	0.00000000	0.57681750	0.05644870
265665_at	AT2G27420	1.82E-03	-0.16483092	0.00000000	-0.18266726	0.16134524
266261_at	AT2G27580	1.13E-03	0.81936836	0.00000000	0.56183050	0.12360025
266247_at	AT2G27660	4.06E-03	1.21769170	0.00000024	1.30430530	-0.05816531
264063_at	AT2G27900	3.18E-03	0.36847878	-0.00000024	0.44296694	-0.10296440
266158_at	AT2G28070	1.11E-03	-0.24516940	0.00000024	-0.19732523	0.12313914
265276_at	AT2G28400	3.05E-04	1.70371940	0.00000000	1.34999300	0.10247827
264078_at	AT2G28470	9.55E-04	-0.79894066	-0.00000048	-0.65721130	-0.04713011
266782_at	AT2G29120	1.19E-03	1.57772590	0.00000000	1.47436240	-0.01675177
266277_at	AT2G29310	8.26E-04	-1.24756000	0.00000000	-1.18435480	0.06439257
266292_at	AT2G29350	3.40E-03	2.52078150	-0.00000024	2.57328130	0.27783870
266289_at	AT2G29390	1.34E-03	-0.29044724	0.00000048	-0.24389744	-0.00348902
266273_at	AT2G29410	2.87E-03	0.67879750	0.00000000	0.71023273	0.00624132
266267_at	AT2G29460	9.78E-04	3.72704460	-0.00000024	3.29705670	0.34596348
266835_at	AT2G29990	1.89E-03	1.77923250	0.00000000	1.77321340	0.13075209
267301_at	AT2G30110	1.95E-03	0.56605196	0.00000048	0.51884794	0.20281649
267300_at	AT2G30140	1.36E-03	1.87731790	0.00000048	1.82287500	0.28511715

267247_at	AT2G30170	3.93E-03	-0.41604853	-0.00000048	-0.62809370	0.15337849
255869_at	AT2G30270	6.83E-04	0.27849580	0.00000048	0.23802567	0.04727507
267471_at	AT2G30390	2.38E-03	-0.69453764	0.00000000	-0.61034440	0.05024576
267496_at	AT2G30550	3.96E-03	2.01494400	0.00000000	1.59902570	-0.14289045
267202_s_at	AT2G31020 /	3.72E-03	0.92103386	-0.00000024	0.59122870	-0.07843256
267152_at	AT2G31040	7.71E-04	-0.85526180	0.00000048	-0.85392190	-0.00562191
266481_at	AT2G31070	2.81E-04	-0.71027994	0.00000000	-0.83135750	0.02045584
263249_at	AT2G31360	5.26E-04	-0.54254913	0.00000048	-0.36743880	0.23469400
263426_at	AT2G31570	7.55E-04	0.56111430	-0.00000048	0.72122480	-0.06005192
263478_at	AT2G31880	1.27E-03	1.98693470	0.00000000	2.17169800	0.00010204
263471_at	AT2G31890	4.01E-03	-0.68109536	-0.00000048	-0.66779780	-0.03947496
263472_at	AT2G31955	3.05E-03	0.85052824	-0.00000048	0.91549635	0.06201887
265728_at	AT2G31990	7.73E-05	0.78085136	0.00000000	1.18853000	-0.35079312
265725_at	AT2G32030	1.59E-03	1.03261780	0.00000024	0.83807470	-0.23024893
265723_at	AT2G32140	3.40E-04	1.63016150	0.00000000	2.07383420	-0.01488948
265679_at	AT2G32240	8.26E-04	1.16997200	0.00000000	0.89951990	-0.09189606
267061_at	AT2G32480	9.90E-04	-0.45936870	-0.00000048	-0.67792270	0.09706783
267549_at	AT2G32640	2.03E-03	-0.90832260	0.00000000	-0.70648193	0.21919203
267546_at	AT2G32680	1.37E-03	2.86740450	0.00000024	3.09125140	0.34773684
267599_at	AT2G32850	2.28E-04	0.24207830	0.00000000	0.23612976	-0.07476711
245166_at	AT2G33170	2.11E-04	0.46443510	0.00000000	0.51482487	-0.13419700
255798_at	AT2G33255	3.20E-04	-0.49452686	0.00000000	-0.42471933	0.15489817
255816_at	AT2G33470	1.61E-03	0.57567310	0.00000000	0.58428526	0.16230583
255845_at	AT2G33600	3.70E-03	0.52705810	0.00000024	0.54772234	0.22466755
255797_at	AT2G33630	3.52E-03	0.84710790	0.00000000	0.80339860	0.07842016
267452_at	AT2G33860	2.53E-04	-0.72588970	-0.00000048	-0.50740814	0.03647995
267023_at	AT2G34250	2.61E-03	0.90756800	0.00000048	0.81218624	0.01441765
266995_at	AT2G34500	1.08E-04	1.37035180	0.00000000	1.18657160	0.02567172
266956_at	AT2G34510	5.48E-04	-0.73491144	0.00000000	-0.61993694	0.26743317
266905_at	AT2G34560	1.43E-03	-0.92259550	0.00000000	-0.82498740	-0.05844212
267310_at	AT2G34680	4.10E-04	-0.94694900	-0.00000048	-0.80907774	-0.11981916
267430_at	AT2G34860	3.38E-04	-0.51840020	-0.00000048	-0.49885654	0.15804672
267412_at	AT2G34940	1.94E-04	0.61213875	0.00000000	0.86618160	-0.41228200
267413_at	AT2G34960	1.10E-03	0.82646203	-0.00000024	0.61402990	0.01336980
266621_at	AT2G35450	1.55E-03	-0.77418900	0.00000000	-0.50694010	-0.17730951
266638_at	AT2G35490	1.83E-04	-0.32551765	0.00000048	-0.33761024	0.04948282
265845_at	AT2G35610	5.89E-04	0.76764490	0.00000000	0.57705880	-0.05732441
263947_at	AT2G35820	3.46E-03	-0.48921490	0.00000000	-0.39859462	-0.27309490
263948_at	AT2G35980	3.52E-03	2.97289040	0.00000024	2.63842300	-0.45438123
263287_at	AT2G36145	1.95E-03	-1.20578240	0.00000000	-1.35642480	0.31665993
263906_at	AT2G36250	2.40E-05	-0.78241110	0.00000048	-0.49887990	0.09246063
263928_at	AT2G36330	4.62E-04	0.73299074	0.00000024	0.60971930	-0.19160843
263846_at	AT2G36990	2.85E-03	-0.78788850	0.00000000	-0.80633545	-0.08702898
265446_at	AT2G37110	6.11E-04	1.00090740	0.00000048	1.15097620	0.10573387
265967_at	AT2G37450	1.02E-03	-0.95263770	0.00000000	-0.90328145	0.23992872
267170_at	AT2G37585	2.71E-03	-0.84020543	0.00000000	-0.86814570	-0.24860382
267165_at	AT2G37710	1.36E-03	1.76874160	0.00000048	1.71941470	-0.00141382
267181_at	AT2G37760	1.53E-03	1.16016600	0.00000000	1.08669300	-0.02079535
266087_at	AT2G37790	3.33E-03	-0.64813540	0.00000000	-0.30740047	0.16058111
267142_at	AT2G38290	7.38E-04	1.55936340	-0.00000048	1.41546920	-0.11327124
267027_at	AT2G38330	2.67E-04	-0.59937096	-0.00000048	-0.62754583	-0.15023184
267036_at	AT2G38465	2.50E-03	-0.96205020	-0.00000024	-1.36498480	-0.33947540
267028_at	AT2G38470	1.83E-03	2.08914000	-0.00000048	1.83498810	-0.30437756
266398_at	AT2G38680	1.38E-03	-0.42029548	-0.00000024	-0.29084563	-0.00023770
266419_at	AT2G38760	3.86E-03	-0.53829430	0.00000000	-0.79050374	0.04792786
266167_at	AT2G38860	8.12E-04	2.22982220	-0.00000048	2.19375560	-0.16194630
266170_at	AT2G39050	1.52E-03	0.58439540	0.00000024	0.58057880	-0.01500964
266968_at	AT2G39360	3.82E-03	0.64408064	0.00000000	0.18424750	-0.28257704
266979_at	AT2G39470	1.69E-05	-1.02217820	-0.00000048	-1.12021160	-0.00233698
266985_at	AT2G39550	3.30E-03	0.60696700	-0.00000024	0.33543515	-0.11995912
267623_at	AT2G39650	3.12E-03	0.86082840	0.00000000	0.79922460	0.11422539
245088_at	AT2G39850	1.95E-03	-0.95686746	-0.00000024	-0.92051480	-0.20178413
267359_at	AT2G40020	1.19E-03	-0.31950712	0.00000000	-0.53842163	-0.01928663
263379_at	AT2G40140	2.45E-03	1.84061190	0.00000048	1.84808350	0.17998362
263830_at	AT2G40260	1.09E-03	-0.35441900	0.00000000	-0.47050428	-0.20900631
263804_at	AT2G40270	3.93E-03	1.22604470	0.00000048	1.28619770	-0.02644062
255827_at	AT2G40600	1.84E-03	0.93684864	0.00000000	0.81404495	-0.23787642
255878_at	AT2G40620	4.13E-03	-0.52810025	0.00000000	-0.75991750	-0.28899193
267069_at	AT2G41010	4.41E-04	1.37343690	0.00000000	1.01489190	-0.01078129
267073_at	AT2G41160	1.61E-03	0.56587744	-0.00000024	0.50362780	-0.12934089
266424_at	AT2G41330	1.64E-03	-0.48609972	0.00000000	-0.48846793	-0.08362389
266371_at	AT2G41410	9.76E-04	2.05779650	-0.00000048	2.20511250	-0.19145012
267103_at	AT2G41490	4.32E-04	0.94959070	0.00000000	0.81709766	0.22135448
260496_at	AT2G41700	1.58E-03	0.69936466	0.00000000	0.42832088	-0.20740986
267537_at	AT2G41880	1.59E-03	0.63339186	0.00000000	0.37175940	-0.33485390
267580_at	AT2G41990	1.33E-03	-1.21546270	0.00000048	-1.30713460	-0.27783012
267635_at	AT2G42220	9.55E-04	-0.77082350	0.00000048	-0.84472750	-0.01292896
265876_at	AT2G42290	1.79E-03	-0.38671374	-0.00000024	-0.24802756	-0.05234218
265883_at	AT2G42310	2.53E-04	0.15280294	0.00000048	0.22211266	-0.09612989
265855_at	AT2G42390	1.43E-03	0.49096918	0.00000048	0.33239650	-0.21917725
265269_at	AT2G42950	1.78E-05	0.48689985	-0.00000024	0.59643410	-0.11533856
265260_at	AT2G43000	1.26E-03	2.81101900	0.00000000	2.47356700	0.17568088
266439_s_at	AT2G43200	1.97E-03	-0.86230090	0.00000000	-0.78678560	0.01079130
260545_at	AT2G43350	1.85E-03	0.49707603	0.00000000	0.51317690	-0.10193157
260542_at	AT2G43560	3.63E-03	-0.48742150	0.00000048	-0.62964580	0.25775576
260556_at	AT2G43620	2.50E-03	1.46714260	0.00000000	1.41803740	0.06741786
267214_at	AT2G43970	8.45E-04	0.89769983	0.00000000	0.87038470	-0.20212650
267189_at	AT2G44180	4.78E-04	0.77245855	0.00000048	0.81764126	-0.21456242
267344_at	AT2G44230	1.48E-03	-0.82936500	0.00000024	-0.79090880	0.14679360

267342_at	AT2G44520	8.26E-04	0.70175457	-0.00000024	0.55550670	0.14832210
266821_at	AT2G44840	2.86E-03	0.78511095	0.00000000	0.79251194	-0.00334501
267500_s_at	AT2G44890 /.	2.14E-03	1.47220660	0.00000000	1.29265500	0.25036025
266104_at	AT2G45150	2.00E-03	-0.46580482	-0.00000024	-0.21295261	0.10313416
251395_at	AT2G45470	1.71E-03	-0.90480330	0.00000000	-0.94490385	0.06648922
266925_at	AT2G45740	1.46E-03	-0.72758675	-0.00000048	-0.59024334	0.09229183
266922_s_at	AT2G45950 /.	3.09E-03	-0.38950825	0.00000000	-0.56667470	-0.03771210
250604_at	AT2G45960 /.	7.61E-05	1.17395350	0.00000000	1.12293390	-0.31533860
266898_at	AT2G45990	7.06E-04	-0.36326647	0.00000000	-0.18402481	0.16324997
266596_at	AT2G46150	7.48E-04	1.14599230	0.00000024	1.29801800	0.29565500
266555_at	AT2G46270	1.11E-04	0.94333170	0.00000000	0.80680320	-0.09318113
263783_at	AT2G46400	9.33E-04	2.77642680	0.00000000	2.84412670	0.16090060
265460_at	AT2G46600	4.18E-03	0.85437536	0.00000000	0.93780470	-0.56927395
265450_at	AT2G46620	5.67E-04	1.25551080	-0.00000024	1.34383200	-0.11620379
266767_at	AT2G46910	9.25E-04	-0.86157750	0.00000048	-1.11419370	-0.08551502
266749_at	AT2G47060	5.41E-04	0.21813393	0.00000000	0.22675419	-0.09189367
266761_at	AT2G47130	3.82E-04	2.18633940	-0.00000024	2.13219240	-0.08557677
260581_at	AT2G47190	7.71E-04	0.63740873	0.00000000	0.46984982	-0.15435600
260530_at	AT2G47320	1.30E-03	0.30514240	0.00000048	0.44077778	0.03775072
260579_at	AT2G47380	7.40E-04	0.84885790	0.00000048	0.73994540	0.21543694
245175_at	AT2G47470	2.16E-03	1.37170270	-0.00000048	1.58629850	0.45496560
245174_at	AT2G47500	4.14E-03	-0.38407730	0.00000024	-0.29725910	0.10856724
251234_s_at	AT2G47650 /.	4.21E-03	0.83809376	-0.00000048	1.08889530	0.28228950
266461_at	AT2G47730	1.49E-06	1.11112210	0.00000000	1.03855470	0.01369524
266464_at	AT2G47800	3.32E-03	2.36115400	0.00000024	2.52204080	0.48239970
266505_at	AT2G47830	1.18E-03	0.50566506	0.00000024	0.29470180	-0.10274410
266460_at	AT2G47930	2.08E-03	-1.19340990	0.00000000	-1.23881530	-0.29158830
265773_at	AT2G48070	1.49E-03	-0.67945194	0.00000048	-0.76267670	-0.12929726
259271_at	AT3G01170	9.04E-05	0.65084696	-0.00000024	0.57341003	-0.11802936
258949_at	AT3G01370	1.88E-04	-0.53101730	0.00000048	-0.65690994	0.00735569
259193_at	AT3G01480	1.07E-03	-0.31350850	-0.00000048	-0.52921534	0.25316525
259191_at	AT3G01720	4.09E-04	1.34873770	-0.00000024	1.40322160	0.16801786
258997_at	AT3G01810	1.29E-03	-0.63137674	-0.00000048	-0.50032760	0.01487160
258947_at	AT3G01830	2.09E-03	3.89635470	0.00000000	3.43186100	-0.03429556
258860_at	AT3G02050	3.83E-03	-0.48546076	0.00000000	-0.36798334	0.10739040
256328_at	AT3G02360	7.79E-04	0.87983704	-0.00000048	0.91402340	0.23296547
258493_at	AT3G02555	1.46E-03	-0.24139047	-0.00000024	-0.13991427	0.18948483
258481_at	AT3G02600	1.93E-03	-0.39318180	0.00000000	-0.34245777	0.04214954
258495_at	AT3G02690	3.85E-03	-0.67740200	0.00000048	-0.73795795	0.04808283
258607_at	AT3G02730	1.43E-03	-0.55339956	0.00000000	-0.47608995	0.01971149
258614_at	AT3G02770	6.13E-04	1.52958200	0.00000048	1.40966370	0.03777504
257536_at	AT3G02800	1.55E-03	0.93641330	0.00000000	0.45092654	-0.37505865
258606_at	AT3G02840	7.94E-04	2.13793560	0.00000000	1.83330250	-0.17339015
259043_at	AT3G03440	4.17E-03	0.59781240	0.00000000	0.68348430	0.04640102
259198_at	AT3G03610	2.20E-03	0.89196587	0.00000000	0.83434390	-0.02810121
258588_s_at	AT3G04120	1.85E-03	0.90292835	-0.00000048	0.96241236	0.38902378
258593_at	AT3G04480	3.47E-03	0.78940580	-0.00000048	0.90760420	0.17485094
258599_at	AT3G04520	2.58E-04	-0.26349258	0.00000000	-0.24160337	0.11399126
258792_at	AT3G04640	3.52E-03	1.37275890	0.00000048	1.04708100	0.00963187
258791_at	AT3G04720	3.95E-03	2.04577350	0.00000000	1.87877800	0.02528572
258804_at	AT3G04760	1.71E-03	-0.47760534	-0.00000048	-0.45138168	0.03220987
259093_at	AT3G04860	2.04E-03	-0.62831880	0.00000000	-0.52609250	-0.11083102
259092_at	AT3G04870	6.37E-04	-0.38932467	-0.00000048	-0.26333237	0.12394428
259308_at	AT3G05180	6.62E-04	-0.91124034	0.00000000	-0.80318740	0.12789440
259297_at	AT3G05360	3.81E-03	0.86858700	-0.00000024	0.49587893	-0.08713985
259105_at	AT3G05500	3.44E-03	1.12603660	0.00000000	1.34410330	-0.00321770
258735_at	AT3G05880	2.73E-03	-0.71080494	0.00000000	-0.70756720	-0.15270376
258750_at	AT3G05910	2.89E-03	-1.01861240	0.00000000	-0.86196613	-0.22356892
258563_at	AT3G05970	5.34E-04	1.16012950	0.00000000	1.01840020	0.05878401
256402_at	AT3G06130	5.76E-05	-0.65816855	-0.00000024	-0.64037920	-0.12504530
256418_at	AT3G06160	2.83E-03	-0.63174963	-0.00000024	-0.48976254	0.15059710
258536_at	AT3G06540	2.90E-03	0.58546734	-0.00000048	0.43197870	0.15702677
258522_at	AT3G06660	3.65E-03	-0.34620930	0.00000048	-0.40719604	-0.06985450
258535_at	AT3G06750	5.12E-04	-0.87000847	0.00000048	-0.77601050	-0.00120211
258524_at	AT3G06810	7.20E-05	0.51446486	0.00000000	0.45676756	0.17707992
258555_at	AT3G06860	1.96E-03	0.78052664	0.00000000	0.83052635	0.25039530
258823_at	AT3G07200	1.31E-03	-0.43881488	0.00000000	-0.17064285	0.08392906
259019_at	AT3G07370	3.26E-03	0.65361450	0.00000024	0.50237940	-0.03372955
259065_at	AT3G07520	2.69E-04	0.97571087	0.00000024	0.83899020	-0.22403050
259021_at	AT3G07540	4.14E-03	-0.57895540	0.00000000	-0.70071316	-0.12871647
259251_at	AT3G07600	2.97E-03	1.04351620	0.00000000	0.71533130	-0.41679263
259256_at	AT3G07680	1.72E-03	0.80675983	0.00000000	0.86668680	0.11393786
259255_at	AT3G07690	5.29E-04	0.49893498	-0.00000024	0.42423130	-0.22249556
259228_at	AT3G07720	2.28E-04	1.03969050	-0.00000048	1.06608300	0.02874613
258643_at	AT3G08010	1.68E-03	-0.52178670	-0.00000048	-0.70857525	-0.04096985
258676_at	AT3G08600	2.57E-04	-0.79892540	0.00000000	-0.58730936	0.14229488
258678_at	AT3G08690	3.04E-04	0.61875250	-0.00000048	0.67084074	0.07371140
258682_at	AT3G08720	3.83E-03	1.48986200	0.00000000	1.25870470	0.07923293
258982_at	AT3G08870	2.80E-04	1.72082710	0.00000024	1.38752700	-0.54229975
258989_at	AT3G08920	3.05E-04	-1.10400150	0.00000000	-1.09416100	-0.14158583
258984_at	AT3G08970	3.32E-03	1.58775160	0.00000000	1.45938940	-0.15024805
259213_at	AT3G09010	2.79E-03	1.80010700	0.00000000	1.58673430	-0.09558153
259037_at	AT3G09350	1.98E-03	0.97483300	-0.00000024	0.97900724	0.26898885
259033_at	AT3G09410	9.57E-04	2.23642830	0.00000000	1.82937100	0.04099107
257535_at	AT3G09490	2.34E-04	1.76608800	0.00000000	1.54170160	-0.13051677
258708_at	AT3G09580	4.50E-04	-0.78712130	0.00000000	-1.01090100	-0.15460396
258696_at	AT3G09650	2.56E-03	-0.45028472	0.00000000	-0.47865010	-0.02855396
258650_at	AT3G09830	1.27E-03	1.27585840	0.00000000	1.34744450	0.13725185
258941_at	AT3G09940	3.80E-03	2.91797880	0.00000024	2.16729450	-0.00707555

258931_at	AT3G10010	3.25E-03	0.48601103	0.00000000	0.50047493	-0.00852418
258927_at	AT3G10160	4.00E-04	-0.63763070	0.00000048	-0.62089490	0.11767292
259150_at	AT3G10320	1.69E-04	0.95410633	-0.00000024	0.57374120	-0.08256960
259148_at	AT3G10350	2.29E-03	-0.20099354	0.00000000	-0.17702866	0.12254429
259156_at	AT3G10380	9.26E-04	0.58597660	0.00000000	0.57761240	0.10460997
258923_at	AT3G10450	3.18E-05	-0.36147547	-0.00000024	-0.10544968	0.04294682
258915_at	AT3G10640	7.24E-05	1.38174150	0.00000000	1.19161270	0.07023025
258759_at	AT3G10800	1.14E-03	0.80887460	-0.00000048	0.58245850	0.12356424
256441_at	AT3G10940	2.49E-04	-1.57902220	0.00000000	-1.08018800	0.08169174
256431_s_at	AT3G11010 /.	6.41E-04	2.58760930	0.00000000	2.39524080	0.09861851
256446_at	AT3G11110	2.86E-03	-0.61222935	-0.00000024	-0.76004650	-0.18170714
256256_at	AT3G11230	9.08E-04	0.60268974	0.00000048	0.65205574	0.11584473
256255_at	AT3G11280	2.95E-04	1.29087920	0.00000000	1.04624700	-0.02645445
256252_at	AT3G11340	8.98E-05	4.60054300	0.00000000	4.04159450	0.24977064
258786_at	AT3G11820	2.38E-03	1.62170410	-0.00000048	1.48153540	0.04113531
258787_at	AT3G11840	7.70E-05	1.88739590	0.00000000	1.58552790	-0.31836700
258731_at	AT3G11880	1.47E-03	0.51291750	0.00000000	0.63460255	0.14787102
256275_at	AT3G12110	5.75E-05	-0.91219260	0.00000024	-1.01461270	0.03548288
256263_at	AT3G12290	6.62E-04	0.47672224	0.00000000	0.43531704	0.17387724
256245_at	AT3G12580	9.25E-04	2.09307480	0.00000000	1.68318200	0.07338572
257700_at	AT3G12740	2.54E-03	1.43690540	0.00000000	1.25317430	-0.06483126
257690_at	AT3G12830	5.87E-04	1.52980280	-0.00000024	1.12537960	-0.22063780
257547_at	AT3G13000	3.16E-03	-1.38650770	0.00000000	-1.21855000	-0.24284363
257861_at	AT3G13050	2.92E-03	0.55315590	0.00000000	0.43535614	-0.24648333
257184_at	AT3G13090	2.09E-03	0.71082616	0.00000000	0.64695430	0.04970527
257185_at	AT3G13100	1.66E-05	2.64061360	-0.00000024	2.60138230	-0.02547216
257190_at	AT3G13120	2.68E-04	-0.61935140	0.00000000	-0.47171068	0.15956402
257657_at	AT3G13235	6.75E-04	0.94480800	0.00000000	1.00800990	0.09524775
257662_at	AT3G13320	1.97E-03	0.30436802	0.00000000	0.17361593	-0.18781757
257708_at	AT3G13330	2.21E-03	1.36110450	-0.00000048	1.61758570	0.28492832
256981_at	AT3G13380	4.22E-04	1.82216690	-0.00000024	1.95310350	0.55922604
256786_at	AT3G13740	2.85E-03	-0.58597900	0.00000000	-0.58646250	0.16552925
256787_at	AT3G13790	1.40E-03	1.37499480	0.00000048	1.43096450	0.11525822
257606_at	AT3G13870	7.23E-04	0.57032824	0.00000000	0.57360170	-0.04962921
258201_at	AT3G13910	1.30E-03	1.81203250	0.00000024	1.44347000	-0.26669955
258203_at	AT3G13950	2.23E-03	2.24434690	0.00000024	2.22730880	-0.10513282
258362_at	AT3G14280	1.22E-03	0.90449050	0.00000000	0.79402566	-0.08092952
258355_at	AT3G14330	5.56E-04	-1.04558250	0.00000024	-0.78311850	0.17841148
257279_at	AT3G14430	2.86E-04	0.66975546	0.00000000	0.51468610	-0.05358553
257277_at	AT3G14470	2.13E-04	1.61313720	-0.00000024	1.46353100	-0.30092740
258063_at	AT3G14620	7.68E-04	1.04779050	0.00000048	0.91554403	-0.30211258
258113_at	AT3G14650	1.70E-03	-0.93287134	0.00000000	-0.62634850	0.39282942
256547_at	AT3G14840	3.08E-03	0.94381620	0.00000000	0.98479940	0.07840443
256856_at	AT3G15110	9.26E-04	-0.58680344	0.00000000	-0.80191660	0.07063437
256854_at	AT3G15180	2.34E-03	0.48392320	0.00000024	0.45089960	-0.06830716
258382_at	AT3G15355	3.21E-03	0.03455925	0.00000000	0.43377113	-0.09485984
257294_at	AT3G15570	1.56E-03	-1.10849550	0.00000000	-0.92231610	0.08621740
258269_at	AT3G15690	4.01E-03	-0.61336994	0.00000048	-0.61089610	0.02455664
258250_at	AT3G15850	5.43E-04	-1.22515110	0.00000000	-1.25883480	-0.03340149
258331_at	AT3G15980	3.60E-03	0.82496880	-0.00000048	0.88010980	0.07570839
258285_at	AT3G16140	2.64E-03	-0.30969095	0.00000000	-0.46873140	-0.04337072
259330_at	AT3G16270	2.89E-03	0.70584820	0.00000000	0.52557420	0.10348177
259379_at	AT3G16350	2.17E-03	-0.50699090	0.00000024	-0.75859520	-0.03715253
257206_at	AT3G16530	1.07E-03	2.56411460	0.00000000	2.31516220	-0.07346439
257895_at	AT3G16950	1.42E-04	-0.98492956	0.00000000	-0.68607400	0.11535072
257888_at	AT3G16990	7.64E-04	0.98947480	0.00000000	0.85458040	-0.02391243
258439_at	AT3G17240	2.05E-03	1.22670840	0.00000000	1.40908430	0.34077263
257295_at	AT3G17420	3.04E-03	1.50158140	0.00000000	1.65627410	0.64358664
258409_at	AT3G17640	3.82E-03	-1.38524100	0.00000000	-1.34110520	-0.23131537
258377_at	AT3G17690	9.66E-04	0.72814370	0.00000000	0.65268920	-0.02854466
258351_at	AT3G17700	2.23E-03	1.08307030	0.00000000	0.63389490	-0.33542442
258156_at	AT3G18050	4.10E-03	-0.71764610	-0.00000048	-0.62874890	0.04534817
257734_at	AT3G18370	2.16E-03	1.05951260	0.00000000	0.98189783	-0.10550475
257751_at	AT3G18690	3.09E-04	0.46940947	0.00000000	0.05306959	-0.25358963
257750_at	AT3G18800	3.88E-03	-0.94714713	0.00000000	-0.97303960	-0.28702188
256657_at	AT3G18860	9.58E-05	0.22168207	0.00000000	0.31660604	-0.07297564
256653_at	AT3G18870	3.79E-04	-0.43816948	0.00000000	-0.50407980	0.14421773
256892_at	AT3G19000	1.93E-03	-0.97554970	-0.00000048	-0.88263680	0.21450186
256922_at	AT3G19010	1.32E-03	1.93466090	0.00000048	1.69087840	0.08327150
257038_at	AT3G19260	5.02E-04	1.25568490	0.00000000	1.21234320	-0.00613117
258025_at	AT3G19480	4.60E-04	-0.89655230	0.00000000	-0.98691535	0.14540386
256627_at	AT3G19970	4.27E-03	0.89776590	-0.00000024	0.77343440	-0.03329063
256624_at	AT3G19990	9.36E-04	0.23259139	0.00000000	0.27924514	-0.22422099
257113_at	AT3G20130	2.44E-03	-0.63621210	-0.00000024	-0.72393584	-0.33736850
257088_at	AT3G20510	4.11E-03	1.25822160	-0.00000048	1.38617990	-0.10124874
256698_at	AT3G20680	3.97E-03	-1.06283570	0.00000048	-1.08219050	-0.11831427
256805_at	AT3G20930	3.25E-03	-0.73937535	0.00000000	-0.87240600	-0.04100919
256969_at	AT3G21080	1.16E-03	1.85607480	0.00000000	1.97264050	0.21017694
258037_at	AT3G21230	4.01E-03	2.21468830	0.00000000	1.69570260	0.25839972
257540_at	AT3G21520	3.23E-04	1.88500810	0.00000000	1.66477870	-0.45020150
258173_at	AT3G21630	4.26E-04	0.96416664	0.00000048	0.81170607	-0.22986078
256795_at	AT3G22110	2.07E-03	0.19341469	0.00000048	0.32854557	0.14037466
256793_at	AT3G22160	1.19E-03	1.59245970	0.00000000	1.41495700	-0.07061124
256937_at	AT3G22620	3.74E-03	2.12191100	0.00000024	1.79960510	0.50486300
257759_at	AT3G23070	3.01E-03	-0.59892035	-0.00000024	-0.82295680	0.16430235
257763_s_at	AT3G23110 /.	3.75E-03	2.29614160	0.00000024	2.21079830	0.22641945
258102_at	AT3G23600	3.88E-03	0.83655980	0.00000000	0.89427090	0.18617249
257202_at	AT3G23750	1.46E-03	-0.49923038	0.00000000	-0.62799454	-0.20772648
257171_at	AT3G23760	7.23E-04	-0.62584760	-0.00000048	-0.69173837	0.28409290

256914_at	AT3G23880	4.18E-03	-1.34379240	0.00000024	-1.55000570	0.18841410
257252_at	AT3G24170	3.79E-03	0.52501200	0.00000000	0.57873870	0.09145117
257249_at	AT3G24180	1.71E-03	1.12462760	-0.00000048	1.15738820	0.15252447
257168_at	AT3G24430	9.24E-04	-0.91297720	0.00000000	-1.06499200	-0.03299761
258133_at	AT3G24500	8.08E-06	1.20950940	0.00000000	1.11339500	0.08279014
257598_at	AT3G24800	1.54E-03	-0.66134690	0.00000000	-0.71863604	-0.15496588
257591_at	AT3G24900	1.78E-03	0.80687930	0.00000024	0.92238927	-0.00908065
257909_at	AT3G25480	4.10E-03	-0.55267644	0.00000048	-0.55637810	0.14383078
256756_at	AT3G25610	5.54E-04	2.00463000	-0.00000024	1.87542920	-0.04097796
256728_at	AT3G25660	3.61E-04	-0.69029950	0.00000000	-0.51534130	0.10936356
257642_at	AT3G25710	4.02E-03	-0.86731243	0.00000000	-0.54738570	0.39140534
257644_at	AT3G25780	3.33E-03	2.39845320	0.00000024	1.83635900	0.11741781
258072_at	AT3G26090	2.50E-04	0.95563220	0.00000000	0.66359687	-0.09722590
257623_at	AT3G26210	3.23E-03	3.13242820	0.00000000	2.93255140	0.27727938
256869_at	AT3G26420 /	7.45E-04	0.95499610	0.00000000	0.91443586	-0.05658031
256883_at	AT3G26440	1.48E-04	1.87846350	-0.00000024	2.06026100	0.32081437
256877_at	AT3G26470	2.66E-03	1.08237270	0.00000024	1.26694870	-0.07219982
252170_at	AT3G26560 /	3.72E-03	3.35010050	-0.00000048	3.45226720	0.32590675
257314_at	AT3G26590	2.31E-05	1.10306980	-0.00000024	1.03307320	0.41061497
257612_at	AT3G26600	9.93E-04	1.33525370	0.00000000	1.19088700	0.06233096
258259_s_at	AT3G26820 /	1.11E-04	2.02375320	0.00000000	1.67807080	0.16526198
258277_at	AT3G26830	1.50E-03	4.80949800	0.00000000	4.49168000	0.45205498
256753_at	AT3G27160	4.62E-04	-0.70854425	0.00000000	-0.57874200	0.02259731
257174_at	AT3G27190	2.69E-03	0.38495398	0.00000024	0.44480420	0.03595853
257713_at	AT3G27380	2.69E-03	0.85928390	0.00000000	0.77592990	0.27419280
257228_at	AT3G27890	8.66E-05	0.86245920	0.00000048	0.75987150	-0.03049517
256851_at	AT3G27930	2.91E-03	0.11663222	0.00000000	0.06467629	-0.04149580
256576_at	AT3G28210	8.08E-04	1.71421340	0.00000000	1.78284880	0.09078050
256633_at	AT3G28340	3.90E-03	2.09693100	0.00000024	1.71907380	-0.02822495
257902_at	AT3G28450	3.11E-04	0.77045010	0.00000000	0.68457747	-0.07021809
256593_at	AT3G28510	4.88E-05	3.39449550	0.00000000	3.42110400	0.18713236
256596_at	AT3G28540	2.17E-03	2.02979180	0.00000000	2.19648890	0.18041658
256989_at	AT3G28580	1.19E-05	2.64418240	0.00000000	2.40914370	0.13213778
256583_at	AT3G28850	4.13E-03	0.58322550	0.00000000	0.57919740	-0.25977826
257139_at	AT3G28890	7.32E-04	0.35148406	0.00000000	0.55588200	0.06027722
258002_at	AT3G28930	3.75E-03	2.03476720	0.00000000	1.76163530	0.05965376
258000_at	AT3G28940	2.59E-03	1.18737840	0.00000000	1.15136200	0.21404552
258001_at	AT3G28950	2.71E-03	0.87331960	0.00000048	0.87491894	0.31076002
257773_at	AT3G29185	8.77E-04	-0.85378313	0.00000048	-0.93917656	0.00237465
257774_at	AT3G29250	1.06E-03	1.07730960	0.00000000	1.17175750	-0.10938549
256735_at	AT3G29400	2.49E-03	0.48805475	0.00000000	0.51781607	-0.13313246
252724_at	AT3G43540	1.31E-03	-0.64759350	0.00000048	-0.59030010	0.32570505
252707_at	AT3G43790	2.49E-03	-0.43783950	0.00000000	-0.30832790	-0.02083850
252652_at	AT3G44720	6.32E-04	1.80043840	-0.00000024	1.77271800	0.19468140
246340_s_at	AT3G44860 /	3.32E-03	2.24639940	0.00000000	1.57086850	-0.32247472
252602_at	AT3G45040	4.10E-03	0.71207240	0.00000000	0.60223820	-0.30262090
252615_at	AT3G45230	4.03E-04	-0.50039910	0.00000000	-0.52535440	-0.00604105
252572_at	AT3G45290	1.50E-04	2.15162600	0.00000000	1.93483920	0.30313350
252587_at	AT3G45620	1.03E-03	1.67256780	0.00000000	1.73048500	0.19774961
252592_at	AT3G45640 /	9.29E-05	0.95456123	0.00000000	0.96165276	-0.20506096
252529_at	AT3G46490	6.46E-04	-0.63410950	-0.00000024	-0.62904050	0.14404368
252473_s_at	AT3G46610 /	3.20E-03	-0.80055140	0.00000000	-0.80031323	0.14473772
252481_at	AT3G46630	2.07E-03	-0.71075060	0.00000048	-0.77308800	0.00926828
252482_at	AT3G46670	4.36E-04	-0.94511150	-0.00000024	-0.87388086	0.26852465
252450_s_at	AT3G47090 /	4.11E-03	1.30631180	-0.00000024	1.21889690	0.03122735
252451_at	AT3G47100	7.77E-04	-0.30306530	0.00000000	-0.31755996	-0.15052414
252459_s_at	AT3G47220 /	2.02E-03	0.32952880	0.00000000	0.19974422	0.09210396
252411_at	AT3G47430	1.79E-03	-0.96042250	-0.00000048	-1.05374740	0.12202215
252417_at	AT3G47480	3.02E-03	2.95997800	0.00000024	3.06984140	0.05297518
252421_at	AT3G47540	3.65E-04	3.38695670	-0.00000024	3.11541130	0.49610066
252431_at	AT3G47700	2.98E-04	0.40673614	0.00000024	0.18467712	-0.01741242
252403_at	AT3G48080	1.41E-03	1.89450550	-0.00000024	1.91023970	-0.20957446
252373_at	AT3G48090	5.22E-05	2.04099320	0.00000000	2.45916130	0.61194180
252353_at	AT3G48200	2.22E-03	-0.76458406	0.00000000	-0.64519310	0.29186583
252365_at	AT3G48350	4.17E-03	-0.61317350	-0.00000024	-0.71836520	-0.01597571
252366_at	AT3G48420	1.45E-03	-1.27088020	0.00000048	-1.37057160	-0.09127140
252345_at	AT3G48640	2.88E-03	2.19671080	0.00000000	2.15277960	-0.18723607
252346_at	AT3G48650	4.94E-04	2.19513340	0.00000000	2.26614200	-0.04872441
252334_at	AT3G48850	3.50E-03	1.67315580	0.00000024	1.45565510	0.08272386
252296_at	AT3G48970	2.68E-03	-0.60544970	0.00000024	-0.79744315	-0.08964324
252280_at	AT3G49260	2.01E-04	-0.94926430	0.00000000	-1.11252780	-0.05355120
252309_at	AT3G49340	1.81E-03	1.74563860	-0.00000024	1.49901510	0.28132534
252310_at	AT3G49350	1.61E-03	1.02340750	-0.00000024	0.92817070	0.03435135
252313_at	AT3G49390	4.11E-04	0.70559263	0.00000000	0.93958380	0.30431460
252261_at	AT3G49500	4.48E-04	-0.20710468	0.00000048	-0.32070112	-0.11261511
252278_at	AT3G49530	1.31E-03	1.06942560	0.00000024	1.21398930	0.00846839
252265_at	AT3G49620	9.53E-04	3.33806180	0.00000000	2.13800530	-0.27314758
252234_at	AT3G49780	9.47E-04	2.21881100	0.00000000	1.73635290	-0.15611935
252223_at	AT3G49850	3.63E-03	-0.25095700	0.00000000	-0.22057605	0.03600597
252214_at	AT3G50260	7.82E-04	1.14864350	0.00000000	1.37740370	0.11440849
252199_at	AT3G50270	1.93E-03	-0.36757946	0.00000048	-0.60354996	-0.07368565
252165_at	AT3G50550	2.06E-03	0.66929126	0.00000024	0.40715456	-0.09068322
252184_at	AT3G50660	4.26E-03	-0.83500910	0.00000000	-1.06352400	0.15694809
252181_at	AT3G50685	8.94E-05	-1.08964350	0.00000000	-1.10494800	-0.07906294
252136_at	AT3G50770	8.43E-05	2.87921290	0.00000000	2.46873710	-0.40418910
252132_at	AT3G50790	5.82E-05	-0.73186445	-0.00000048	-0.73150660	-0.05600739
252130_at	AT3G50820	8.00E-04	-0.65113830	0.00000000	-0.68196250	0.10508108
252131_at	AT3G50930	1.01E-03	2.83207500	0.00000024	2.87615010	0.08546805
252126_at	AT3G50950	8.34E-04	0.79262540	0.00000000	0.76238537	-0.28073550

252098_at	AT3G51330	9.37E-04	2.55210640	0.00000000	2.53519100	-0.10803366
252092_at	AT3G51420	2.30E-03	-0.94010260	0.00000000	-1.04969690	0.04558039
252117_at	AT3G51430	2.39E-03	1.51885180	0.00000000	1.49777700	-0.05384541
252068_at	AT3G51440	2.96E-03	1.66097830	-0.00000024	1.49987600	-0.38610458
252116_at	AT3G51510	7.56E-04	-0.85280610	-0.00000048	-0.94389250	-0.09975243
252112_at	AT3G51580	2.64E-03	0.56109570	0.00000000	0.57612085	0.03259873
246305_at	AT3G51890	1.17E-06	1.23941760	-0.00000048	1.09199620	-0.31739210
256682_at	AT3G52200	1.53E-03	0.47348928	-0.00000048	0.56645820	0.04319334
256680_at	AT3G52230	3.86E-03	-0.53350353	0.00000000	-0.46338797	0.00791407
252053_at	AT3G52400	5.59E-04	1.83628370	-0.00000024	1.70067260	-0.23308325
252060_at	AT3G52430	1.18E-03	3.59896470	-0.00000024	3.48153110	0.59488800
252047_at	AT3G52490	2.50E-03	-0.33743906	0.00000024	-0.19440746	0.30625010
252008_at	AT3G52610	2.03E-03	-0.35390520	0.00000048	-0.49081635	-0.07498884
252023_at	AT3G52920	5.33E-04	-0.44686030	0.00000000	-0.50330377	0.32703876
251969_at	AT3G53130	1.99E-03	-0.43594265	0.00000000	-0.41872072	0.29105043
251970_at	AT3G53150	7.73E-04	1.26262660	0.00000024	1.41366740	-0.22995949
251972_at	AT3G53170	1.73E-03	-0.58437680	0.00000000	-0.36844206	-0.06483316
251975_at	AT3G53230	6.58E-05	2.06491610	0.00000000	1.92692850	0.11557841
251930_at	AT3G53780	1.83E-04	0.73629236	0.00000048	0.66152050	-0.04292917
251910_at	AT3G53810	3.06E-03	1.17422820	0.00000000	0.92726517	-0.06205225
251917_at	AT3G53970	1.65E-03	0.24528980	-0.00000048	0.28389835	-0.13465977
251932_at	AT3G54010	2.92E-04	0.57644176	0.00000000	0.54511120	0.07995367
251884_at	AT3G54150	9.89E-05	2.20109130	0.00000000	2.10229400	-0.43382406
251847_at	AT3G54640	5.23E-04	2.48794080	0.00000000	2.29057500	0.10969257
251860_at	AT3G54660	2.23E-05	-0.45235348	0.00000048	-0.43461275	0.09038067
251839_at	AT3G54950	1.25E-04	1.11679790	0.00000000	1.26757740	0.57714130
251823_at	AT3G55080	8.34E-05	-0.44562100	-0.00000048	-0.62386630	-0.15437770
251832_at	AT3G55150	2.75E-03	1.09065510	0.00000000	0.68671010	-0.30462550
251790_at	AT3G55470	1.17E-04	1.44027850	-0.00000024	1.21541600	0.03543711
251759_at	AT3G55630	1.82E-03	-1.04720350	0.00000048	-1.07917790	0.23769140
251761_at	AT3G55700	2.58E-03	0.84675956	0.00000000	0.68612860	0.16257739
251762_at	AT3G55800	9.88E-05	-0.58919144	-0.00000048	-0.70407295	0.15772343
251769_at	AT3G55950	6.46E-04	1.99907400	-0.00000024	1.86559580	-0.00348115
251744_at	AT3G56010	3.05E-03	-0.30411577	0.00000000	-0.33019638	0.41675568
251736_at	AT3G56130	2.67E-04	-0.62169740	-0.00000048	-0.73073290	-0.07846117
251720_at	AT3G56160	2.03E-03	-0.84513760	0.00000000	-0.57709860	0.15040755
251739_at	AT3G56170	2.13E-03	1.22935390	0.00000000	1.32138200	0.15550923
251705_at	AT3G56400	3.01E-03	2.13761430	-0.00000048	1.87803320	-0.26823664
251684_at	AT3G56410	1.48E-03	0.66945650	0.00000000	0.23755598	-0.44208765
251701_at	AT3G56650	2.66E-03	-0.37967920	0.00000000	-0.49484062	0.10327721
246293_at	AT3G56710	3.06E-03	1.31904320	0.00000000	1.13274340	-0.14817238
251659_at	AT3G57090	1.43E-04	0.57550955	0.00000000	0.65455246	-0.11954498
251670_at	AT3G57190	2.31E-03	-0.73510550	0.00000000	-0.71765160	-0.02301693
251672_at	AT3G57230	4.24E-03	0.42432594	0.00000000	0.26274943	-0.08371544
251673_at	AT3G57240	2.03E-03	3.85970350	0.00000000	3.78656000	0.30115938
251624_at	AT3G57280	1.74E-03	0.95107126	0.00000000	0.79982760	-0.17430496
251649_at	AT3G57330	1.74E-04	1.14603280	0.00000048	0.93924570	-0.38090324
251633_at	AT3G57460	7.60E-04	1.92118050	0.00000000	1.58956190	-0.32200290
251621_at	AT3G57700	2.31E-03	1.22268920	-0.00000024	1.02079340	-0.23045278
251647_at	AT3G57770	4.17E-03	-0.74757195	0.00000000	-0.68870735	-0.14371014
251612_at	AT3G57950	1.93E-03	0.54326415	0.00000000	0.75920770	-0.05788231
251526_at	AT3G58640	3.47E-03	0.53252983	0.00000048	0.48214293	-0.16858149
251507_at	AT3G59080	2.83E-03	0.55182600	-0.00000024	0.39038014	-0.45638967
251521_at	AT3G59420	3.54E-03	-0.54491854	0.00000000	-0.43994212	0.04149461
251475_at	AT3G59660	3.51E-04	1.54866120	0.00000024	1.43471340	0.11884975
251479_at	AT3G59700	7.90E-05	1.63035870	0.00000000	1.43331150	-0.12240338
251461_at	AT3G59780	4.29E-03	-0.77822210	0.00000000	-0.75688170	-0.17845535
251449_at	AT3G59920	1.62E-04	0.47255564	0.00000000	0.46007776	-0.11695623
251456_at	AT3G60120	3.56E-03	0.85986710	-0.00000024	0.66393950	-0.06196189
251406_at	AT3G60260	2.77E-03	1.12442300	0.00000000	1.20313740	0.22433567
251414_at	AT3G60370	6.52E-04	-0.59888360	0.00000000	-0.63705444	0.05696011
251400_at	AT3G60420	3.00E-04	3.21592950	0.00000048	3.10922670	0.23666954
251370_at	AT3G60450	1.87E-04	2.12911500	0.00000000	2.08170220	0.17826033
251419_at	AT3G60470	3.14E-05	1.18696310	0.00000000	1.31585600	-0.11436462
251386_at	AT3G60800	1.72E-03	0.76243640	0.00000048	0.85720253	0.21343708
251390_at	AT3G60860	1.85E-03	0.57293270	0.00000000	0.45149470	-0.05841017
251353_at	AT3G61080	1.73E-03	-0.84203150	0.00000000	-0.96301913	0.08727741
251336_at	AT3G61190	7.20E-05	2.78399730	0.00000000	2.66871550	-0.00089169
251330_at	AT3G61550	2.08E-03	-0.97881603	0.00000000	-1.18538860	-0.22325134
251289_at	AT3G61830	1.10E-03	-0.51151466	0.00000000	-0.49272943	-0.11993957
251243_at	AT3G61870	2.39E-03	-0.63993216	0.00000000	-0.73135376	0.11439085
251218_at	AT3G62410	1.14E-03	-0.70548200	0.00000000	-0.85168743	-0.05199146
251222_at	AT3G62580	4.03E-03	0.87573195	0.00000000	0.71608160	0.20113945
251191_at	AT3G62590	1.83E-03	0.46906996	0.00000024	0.36063385	-0.44460750
251227_at	AT3G62700	9.70E-05	-0.48536300	-0.00000048	-0.38207197	0.22950983
251232_at	AT3G62780	2.64E-03	1.22556970	0.00000000	0.96634220	-0.29754280
251200_at	AT3G63010	3.32E-03	1.15614580	0.00000024	1.49423120	0.34591770
251205_at	AT3G63080	1.99E-04	0.85677340	0.00000048	0.64997290	-0.18303728
251150_at	AT3G63120	1.75E-03	-0.73425840	0.00000000	-0.59124040	-0.20178223
251157_at	AT3G63140	1.97E-04	-0.72718570	0.00000048	-0.80781840	0.15883636
251176_at	AT3G63380	9.07E-04	2.64082480	0.00000000	2.47510340	-0.16230583
251121_at	AT3G63420	1.10E-03	1.10302730	0.00000024	0.81531715	0.01424575
251179_at	AT3G63460	3.73E-04	0.56794550	0.00000048	0.59262896	0.08984327
251120_at	AT3G63490	2.43E-03	-0.53317976	0.00000000	-0.40659142	0.25385952
255709_at	AT4G00180	1.22E-03	-0.57476280	0.00000048	-0.50509050	-0.13079596
255691_at	AT4G00370	3.43E-03	-0.53390410	0.00000048	-0.36538792	0.16725492
255674_at	AT4G00430	3.56E-03	-0.72344400	-0.00000048	-0.48384714	0.27519083
255681_at	AT4G00550	1.58E-03	0.48445868	0.00000000	0.42022014	-0.20757055
255630_at	AT4G00700	1.19E-03	2.45365140	0.00000024	2.39689600	-0.12204886

255637_at	AT4G00750	2.72E-03	-0.66430500	-0.00000024	-0.93854310	-0.21454310
255660_at	AT4G00755	1.24E-03	-0.82484436	0.00000000	-0.80960060	0.25761510
255645_at	AT4G00880	2.10E-03	-0.74977636	0.00000000	-0.65767000	0.01511383
255627_at	AT4G00955	1.04E-03	0.79676770	0.00000000	0.64312506	-0.26929522
255599_at	AT4G01010	1.51E-04	1.43889140	0.00000024	1.43660640	-0.18754101
255572_at	AT4G01050	5.53E-04	-0.58099794	0.00000000	-0.45282364	0.09117937
255607_at	AT4G01130	3.44E-03	-1.31268690	0.00000000	-1.42910430	-0.31796074
255623_at	AT4G01310	1.53E-03	-0.63639164	0.00000048	-0.57397795	0.22914553
255624_at	AT4G01370	2.15E-03	1.10359950	0.00000000	0.96992730	0.12568188
255621_at	AT4G01390	3.06E-03	-0.89134410	0.00000000	-0.11866951	0.59447720
255595_at	AT4G01700	4.16E-03	2.41453650	-0.00000024	2.39454030	0.07109642
255596_at	AT4G01720	3.46E-03	1.27173800	-0.00000024	0.98345065	-0.07760239
255560_at	AT4G02030	1.78E-03	0.39549685	-0.00000048	0.25006008	-0.09904623
255506_at	AT4G02130	6.12E-04	-0.75572490	0.00000000	-0.88419724	-0.36590195
255479_at	AT4G02380	1.33E-03	2.77530530	0.00000048	2.57729960	-0.34600973
255503_at	AT4G02420	3.33E-04	1.25294730	0.00000000	1.14250330	-0.23962665
255499_at	AT4G02730	2.21E-03	0.69623900	0.00000000	0.65685964	0.14473080
255452_at	AT4G02880	4.22E-03	0.12893248	0.00000048	0.17362880	-0.33626533
255410_at	AT4G03100	1.84E-03	-0.51365470	-0.00000048	-0.36819530	0.28979874
255436_at	AT4G03150	4.19E-03	-0.91022680	0.00000000	-0.72747900	-0.02102757
255402_at	AT4G03205	2.25E-03	-0.51328635	0.00000000	-0.27554345	0.15390301
255406_at	AT4G03450	1.32E-03	2.82311580	-0.00000024	2.62782670	-0.18301797
255348_at	AT4G03820	3.61E-03	0.95350456	0.00000000	0.97039130	0.13155007
255319_at	AT4G04220	6.09E-04	2.37281500	-0.00000024	2.14598900	0.15718412
255331_at	AT4G04330	5.34E-04	-1.03008990	-0.00000048	-1.01453020	-0.15725088
255332_at	AT4G04340	2.22E-03	-0.70711565	-0.00000048	-0.69812630	0.01260710
255340_at	AT4G04490	2.01E-04	2.41392100	0.00000000	2.29272250	-0.20605826
255341_at	AT4G04500	2.19E-03	2.53257230	0.00000024	2.68232540	0.00217223
255342_at	AT4G04510	3.18E-05	0.70735526	0.00000000	1.18831560	-0.00545454
255344_s_at	AT4G04540 /	3.62E-03	1.30816080	-0.00000048	1.21240380	-0.02728128
255301_at	AT4G04800	4.15E-03	0.74469376	0.00000024	0.49257612	-0.06892228
255298_at	AT4G04840	1.61E-03	-1.21389870	0.00000000	-1.55127330	-0.08756685
255277_at	AT4G04890	6.92E-04	-0.48979664	0.00000000	-0.34573126	-0.00690413
255308_at	AT4G04910	2.80E-04	0.65307810	-0.00000048	0.74622440	0.23617363
255280_at	AT4G04960	8.53E-04	0.52583337	-0.00000024	0.62709760	0.06006837
255265_at	AT4G05190	4.16E-03	-0.18932915	0.00000000	-0.20629478	0.45051240
255243_at	AT4G05590	3.07E-03	0.69628096	0.00000000	0.82168220	-0.05921865
255193_at	AT4G07400	5.08E-04	-0.39330983	0.00000000	-0.44914390	-0.07914352
255151_at	AT4G08180	4.11E-04	0.69468427	-0.00000024	0.50027990	0.03161740
255117_s_at	AT4G08700 /	2.42E-03	-0.21625924	0.00000000	-0.11122489	-0.00675750
255110_at	AT4G08770	1.21E-04	2.55028200	0.00000000	2.77658990	-0.01068759
255111_at	AT4G08780	1.82E-03	1.17345900	0.00000024	0.86824536	-0.07658458
255116_at	AT4G08850	3.67E-04	1.39398570	0.00000000	1.42703960	-0.14109468
255078_at	AT4G09010	9.18E-04	-0.69199276	-0.00000048	-0.82859707	-0.05779171
255082_at	AT4G09160	1.59E-03	-0.59190370	0.00000024	-0.72080300	-0.35407520
255043_at	AT4G09640	1.53E-03	-0.50557400	-0.00000024	-0.34655166	-0.10726857
255810_at	AT4G10140	1.56E-03	0.46930170	0.00000048	0.46708155	-0.02065420
255019_at	AT4G10210	3.15E-03	-0.18686199	0.00000024	-0.23229504	0.04791546
254970_at	AT4G10340	1.45E-04	-0.03354073	0.00000048	-0.05422449	0.05746651
254982_at	AT4G10470	1.65E-03	-0.45637798	0.00000000	-0.52080107	0.45877218
254975_at	AT4G10500	3.11E-03	3.01455600	0.00000000	3.09691520	0.73483420
254952_at	AT4G10955 /	3.29E-04	1.27553800	0.00000000	1.04518530	-0.20789933
254948_at	AT4G11000	1.71E-03	0.60096980	-0.00000024	0.53999950	-0.38722944
254965_at	AT4G11090	1.56E-03	0.19588375	0.00000000	0.19238424	-0.04532766
254905_at	AT4G11170	1.67E-03	0.65874790	-0.00000024	0.73754334	-0.18149662
254926_at	AT4G11280	1.32E-03	2.16370530	0.00000024	2.04365830	0.56856130
254922_at	AT4G11370	1.49E-03	1.47066430	-0.00000024	1.14579150	0.08820319
254897_at	AT4G11470	3.35E-04	0.55187510	-0.00000024	0.19752765	-0.07757044
254891_at	AT4G11740	1.71E-03	0.56590080	0.00000000	0.33589650	0.00621748
254894_at	AT4G11840	2.20E-03	1.85968280	0.00000024	1.16312840	-0.05499721
254847_at	AT4G11850	2.06E-04	1.45666050	0.00000024	1.13671370	-0.06982517
254869_at	AT4G11890	1.07E-03	3.20246600	0.00000000	3.07846300	0.31679726
254863_at	AT4G11980	1.37E-03	-0.47383046	0.00000048	-0.56776667	0.15148067
254857_at	AT4G12120	1.15E-03	0.98919510	0.00000000	0.87936830	-0.13081002
254833_s_at	AT4G12280 /	3.00E-03	1.09479520	0.00000048	0.83135414	-0.06642628
254835_s_at	AT4G12310 /	1.76E-04	-0.81972504	0.00000048	-0.94370080	0.31550837
254815_at	AT4G12420	1.12E-03	-1.01212070	-0.00000048	-0.96505930	-0.20572281
254784_at	AT4G12720	3.12E-04	2.18795540	0.00000000	2.13202950	-0.02091885
254790_at	AT4G12800	3.75E-03	-0.04783344	0.00000000	-0.19249153	0.11346388
254783_at	AT4G12830	4.19E-03	-1.46728610	-0.00000048	-1.45013930	0.17132092
254791_at	AT4G12910	1.52E-03	-0.49006940	0.00000000	-0.35547304	0.15865421
254745_at	AT4G13460	6.96E-04	-0.43767880	0.00000000	-0.53276160	-0.17898369
254723_at	AT4G13510	7.75E-04	1.48150540	-0.00000048	1.18620060	0.05995894
254727_at	AT4G13670	1.11E-03	-0.41679573	0.00000048	-0.62024640	0.02535725
254741_s_at	AT4G13900 /	5.40E-04	2.66355630	0.00000000	2.54558800	0.02889490
245284_at	AT4G14210	2.67E-03	-0.68160343	0.00000000	-0.70682430	-0.10828781
245329_at	AT4G14365	1.21E-03	3.07922890	0.00000000	2.85572620	0.12710667
245611_at	AT4G14390	3.23E-03	0.71241190	0.00000024	0.60503580	-0.17658854
245566_at	AT4G14610	1.24E-05	1.37624050	0.00000000	1.22987410	-0.29774046
245315_at	AT4G14800	2.17E-04	0.85410120	0.00000048	0.95463514	0.15518999
245396_at	AT4G14870	4.10E-03	-0.67790985	0.00000048	-0.83656883	-0.11282969
245298_at	AT4G15010	2.93E-03	0.62679960	0.00000000	0.64831257	0.05198765
245545_at	AT4G15280	3.70E-03	0.37292147	0.00000024	0.44891882	0.02279329
245289_at	AT4G15470	7.39E-04	0.70384980	0.00000000	0.70063780	0.04522419
245317_at	AT4G15610	1.73E-03	1.88682750	0.00000048	1.46122500	0.10424280
245291_at	AT4G16155	3.40E-03	-0.51512290	0.00000048	-0.78270150	-0.02068186
245393_at	AT4G16260	3.21E-03	3.04622840	0.00000024	2.56969450	0.10448337
245338_at	AT4G16442	3.98E-03	-0.58913183	0.00000000	-0.69681597	-0.09907579
245318_at	AT4G16980	2.32E-03	-0.50692034	0.00000048	-0.71863030	-0.11895084

245266_at	AT4G17070	2.45E-03	1.58588080	0.00000000	1.23855260	-0.26434207
245417_at	AT4G17360	8.76E-04	-1.01956870	-0.00000024	-1.17024920	0.13173366
245395_at	AT4G17420	2.39E-03	0.44225073	0.00000000	0.42690277	-0.15174246
245357_at	AT4G17560	8.00E-04	-0.60982084	0.00000000	-0.50330020	0.13049030
245428_at	AT4G17570	8.11E-04	0.57077456	0.00000000	0.57953690	0.20930862
245354_at	AT4G17600	1.78E-03	-0.55116940	-0.00000048	-0.63233566	0.24959326
245401_at	AT4G17670	3.05E-03	1.44960600	0.00000000	1.66402050	0.27288938
245365_at	AT4G17720	2.91E-03	1.34913250	0.00000048	1.38023900	0.17042351
254690_at	AT4G17830	6.22E-05	0.85284233	-0.00000048	0.83473396	-0.02165222
254660_at	AT4G18250	1.19E-05	2.80680660	0.00000024	2.44639870	-0.23214817
254669_at	AT4G18370	2.96E-04	-0.89038086	-0.00000048	-0.93509390	-0.06891441
254670_at	AT4G18390	2.86E-03	-0.83043240	0.00000000	-0.81997110	-0.10542059
254673_at	AT4G18430	2.12E-03	2.60772000	0.00000000	2.39629750	-0.03623390
254646_at	AT4G18530	6.36E-04	-0.42868543	0.00000024	-0.25106573	-0.07905459
254649_at	AT4G18570	5.06E-04	-1.09610220	0.00000000	-1.01968530	-0.27097560
254624_at	AT4G18580	3.21E-05	0.84000635	0.00000024	0.71033790	-0.06928706
254643_at	AT4G18820	2.71E-03	-0.37122846	0.00000024	-0.24298239	0.13361740
254605_at	AT4G18950	1.88E-03	0.54609275	0.00000000	0.47305298	0.00048113
254604_at	AT4G19070	3.39E-03	-0.49669957	0.00000048	-0.28200245	0.01880407
254612_at	AT4G19100	3.14E-03	-0.72708370	0.00000000	-0.84934900	-0.15216350
254571_at	AT4G19370	2.33E-04	2.34412860	-0.00000024	1.71860310	0.14309049
254553_at	AT4G19530	2.28E-03	-0.95017815	0.00000000	-1.22403760	-0.02731276
254545_at	AT4G19830	6.06E-04	-0.79858350	-0.00000048	-1.04646920	0.23222542
254505_at	AT4G19985	1.09E-03	-0.61842610	0.00000000	-0.61327980	0.13910866
254524_at	AT4G20000	4.38E-04	1.47584370	0.00000024	1.47431160	-0.11486816
254500_at	AT4G20110	8.12E-04	2.57518820	0.00000024	2.49240160	0.23509932
254478_at	AT4G20330	2.48E-03	0.28926420	0.00000000	0.25538063	-0.02289867
254466_at	AT4G20430	1.92E-03	-0.77125310	0.00000000	-0.50005627	-0.04221201
254432_at	AT4G20830	2.29E-03	2.18954370	0.00000000	1.85368200	0.37066364
254431_at	AT4G20840	3.56E-04	-0.34739614	-0.00000024	-0.83777523	0.25187254
254453_at	AT4G21120	1.35E-03	1.29697850	0.00000000	1.02594180	-0.00716591
254416_at	AT4G21380	2.03E-03	1.70808740	0.00000000	1.41796060	-0.22615695
254408_at	AT4G21390	2.60E-04	2.14850020	-0.00000024	1.26981590	-0.19634128
254409_at	AT4G21400	1.28E-05	1.78419970	0.00000000	1.22490720	-0.38051224
254372_at	AT4G21620	2.94E-03	-0.49183273	0.00000000	-0.37358665	0.07054186
254385_s_at	AT4G21830 /	1.90E-03	3.62133120	-0.00000024	3.39079900	0.26578212
254387_at	AT4G21850	6.84E-04	2.41660980	0.00000000	2.21263220	-0.31723404
254388_at	AT4G21860	1.20E-03	-0.58226680	0.00000000	-0.67489815	0.11170006
254335_at	AT4G22260	1.88E-05	0.68242500	0.00000024	0.79297686	0.20884752
254318_at	AT4G22530	5.01E-04	1.87373210	0.00000024	1.56936980	0.00826836
254276_at	AT4G22820	3.89E-03	0.57067394	0.00000048	0.35166454	-0.06641150
254289_at	AT4G22980	2.06E-03	1.14295360	0.00000000	0.78097060	-0.22888231
254292_at	AT4G23030	6.28E-04	0.68419840	0.00000024	0.81089450	-0.01718235
254266_at	AT4G23130	2.13E-03	1.51540450	0.00000000	1.19827440	-0.58338760
254265_s_at	AT4G23140 /	1.26E-03	2.95145560	-0.00000024	3.00483230	0.11212039
254256_at	AT4G23180	6.16E-05	0.66917086	0.00000000	0.44170475	-0.39034700
254241_at	AT4G23190	1.18E-04	1.34270260	-0.00000024	1.48260640	-0.03648329
254242_at	AT4G23200	1.00E-03	1.14796640	-0.00000048	0.99588394	-0.44870758
254243_at	AT4G23210	1.51E-03	2.40085740	0.00000024	2.54045060	0.31254816
254255_at	AT4G23220	3.77E-04	2.67527250	0.00000000	2.37982560	-0.33837366
254261_at	AT4G23460	1.14E-05	0.80919313	0.00000000	0.76912355	0.02540302
254262_at	AT4G23470	6.17E-04	1.69435790	-0.00000048	1.50561100	-0.11670589
254211_at	AT4G23570	2.93E-03	1.60527990	0.00000000	1.53090050	0.11175203
254229_at	AT4G23610	3.85E-04	2.30652710	-0.00000024	2.36370470	-0.13973522
254224_at	AT4G23650	2.11E-03	0.79706000	0.00000000	0.72103500	0.27287054
254215_at	AT4G23700	1.81E-03	2.52747770	0.00000024	2.45432100	0.30605220
254231_at	AT4G23810	1.87E-03	3.05246260	0.00000000	2.81734000	-0.09265304
254221_at	AT4G23820	1.45E-03	-0.90393114	0.00000000	-0.95620060	-0.20016909
254192_at	AT4G23850	3.85E-03	0.95559500	0.00000000	0.94888450	-0.11008692
254190_at	AT4G23885	2.76E-03	2.02324870	0.00000000	1.88743230	0.15891695
254166_at	AT4G24190	2.46E-03	1.56482030	-0.00000048	1.71796660	0.25218630
254125_at	AT4G24670	1.21E-03	-0.67920756	-0.00000024	-0.72884180	0.04718399
254118_at	AT4G24790	4.17E-03	-0.59714840	0.00000000	-0.45648813	0.09003854
254103_at	AT4G25030	3.04E-04	0.64427090	-0.00000048	0.71755600	-0.08531046
254091_at	AT4G25070	1.35E-03	0.39380240	-0.00000024	0.53404000	-0.08158875
254105_at	AT4G25080	1.15E-04	-0.79408836	-0.00000048	-0.79688690	0.08748198
254093_at	AT4G25110	1.25E-03	1.56856780	-0.00000024	1.67284870	-0.26529694
254060_at	AT4G25350	3.24E-04	0.33260750	-0.00000024	0.21844530	-0.02335930
254063_at	AT4G25390	3.23E-03	1.06107520	0.00000000	0.70006250	-0.20295572
254077_at	AT4G25640	9.16E-04	0.75872755	0.00000000	0.93189670	0.16076660
254020_at	AT4G25700	2.29E-03	-0.65095377	0.00000000	-0.96504590	0.08684921
254082_at	AT4G25720	2.26E-03	0.78394890	0.00000000	0.55704020	0.02981520
254024_at	AT4G25780	9.77E-05	-2.05992940	0.00000024	-2.25027280	0.28915262
254040_at	AT4G25900	1.70E-03	1.47635840	-0.00000048	1.55915260	0.25647163
254032_at	AT4G25940	3.40E-03	0.22175932	0.00000000	0.00644207	-0.49413824
253992_at	AT4G26060	2.90E-04	1.44031910	0.00000000	1.20049290	-0.11386299
253993_at	AT4G26070	2.20E-03	1.04741000	0.00000048	0.89578676	-0.20422268
254010_at	AT4G26240	1.25E-03	0.56948230	-0.00000048	0.65843487	0.04269600
253987_at	AT4G26270	2.50E-03	1.21177530	0.00000000	1.42035250	0.04946137
253966_at	AT4G26520	7.45E-04	-0.94473934	0.00000000	-1.13071610	0.11477876
253956_at	AT4G26700	2.79E-03	-0.62191060	0.00000000	-0.79378510	0.03576756
253946_at	AT4G26790	3.19E-03	-0.56975390	0.00000000	-0.52444840	0.01982021
253950_at	AT4G26910	1.96E-03	1.39884420	0.00000000	1.40259270	-0.03847885
253923_at	AT4G27060	1.62E-04	-0.13214016	0.00000048	-0.21605492	-0.01008415
253915_at	AT4G27280	2.57E-04	1.94766330	0.00000000	1.89531760	-0.28034496
253911_at	AT4G27300	1.06E-03	0.89890146	0.00000000	0.65426207	-0.31797980
253860_at	AT4G27700	1.00E-03	-0.78685570	-0.00000048	-0.59492590	0.30388880
253825_at	AT4G28025	1.22E-03	-0.64883040	0.00000000	-0.95385265	-0.07939053
253855_at	AT4G28050	2.61E-03	-0.64142130	0.00000000	-0.60702230	-0.10810566

253811_at	AT4G28190	2.31E-03	-0.85242580	-0.00000024	-0.92865396	-0.49690676
253776_at	AT4G28390	1.93E-05	1.82649330	-0.00000024	1.90373040	-0.05500078
253779_at	AT4G28490	1.06E-04	1.92584990	0.00000024	1.61422730	0.07930708
253789_at	AT4G28570	8.04E-05	0.47034264	0.00000000	0.56358430	-0.15315580
253755_at	AT4G29040	3.68E-04	0.74250840	-0.00000048	0.81540010	0.04756451
253747_at	AT4G29050	3.32E-03	0.48066425	0.00000000	0.23344111	-0.42199730
253735_at	AT4G29160	2.45E-03	0.80494880	-0.00000024	0.78379726	0.18973112
253708_at	AT4G29210	1.09E-04	0.67375994	-0.00000024	0.74941780	0.05904031
253712_at	AT4G29330	8.10E-04	1.15393880	0.00000000	0.93385840	0.26548338
253696_at	AT4G29740	3.90E-03	1.21643070	0.00000000	1.63553520	0.27766370
253643_at	AT4G29780	1.73E-03	1.05714540	0.00000000	0.86764720	-0.23950863
253646_at	AT4G29810	3.45E-03	1.37484650	0.00000000	1.39120390	0.05927658
253702_at	AT4G29900	2.20E-03	1.46164370	0.00000000	1.34209590	-0.03349638
253642_at	AT4G29960	1.30E-03	0.63209250	0.00000000	0.59146070	0.12225151
253624_at	AT4G30580	3.73E-04	-0.51261140	-0.00000048	-0.57436560	0.07642222
253579_at	AT4G30610	2.97E-03	-1.06720760	-0.00000024	-1.10996820	0.16750193
253547_at	AT4G30950	1.00E-03	-0.43463040	0.00000000	-0.55564930	-0.02361298
253546_at	AT4G31040	4.14E-03	-0.33605720	0.00000000	-0.31268024	0.02161408
253518_at	AT4G31400	2.36E-03	-0.37214446	0.00000000	-0.36711980	-0.02686596
253534_at	AT4G31500	2.70E-03	1.49770500	0.00000048	1.50542120	0.06438875
253530_at	AT4G31530	1.17E-03	-0.41667128	0.00000048	-0.46935368	0.07378721
253537_at	AT4G31560	1.03E-03	-0.56963370	0.00000000	-0.57384040	-0.01652765
253533_at	AT4G31590	4.67E-04	-0.66165090	-0.00000048	-0.44768380	-0.08137298
253485_at	AT4G31800	4.03E-03	3.05651280	0.00000000	2.82885220	0.31779718
253429_at	AT4G32420	1.99E-03	-0.46830177	-0.00000048	-0.29399610	-0.06135321
253440_at	AT4G32570	1.19E-03	-1.09748320	0.00000000	-0.84951925	0.17843151
253389_at	AT4G32680	1.00E-04	0.43203545	0.00000000	0.38816595	0.00443983
253401_at	AT4G32870	2.48E-03	1.51869680	0.00000000	1.23392720	-0.39853525
253406_at	AT4G32890	3.50E-03	-0.44940400	0.00000000	-0.58996940	0.04512668
253411_at	AT4G32980	3.46E-03	-0.98319890	-0.00000024	-0.49525690	0.50015260
253387_at	AT4G33010	9.58E-04	-0.81374980	0.00000000	-0.68952320	0.12670040
253414_at	AT4G33050	1.43E-03	2.49607320	0.00000000	2.37949600	-0.08074522
253361_at	AT4G33100	1.19E-05	0.50406980	0.00000024	0.28365470	-0.08772874
253334_at	AT4G33360	3.92E-03	1.04482560	-0.00000048	1.01447300	-0.04858589
253337_at	AT4G33470	1.50E-03	-0.67015980	0.00000000	-0.71438694	0.03196383
253343_at	AT4G33540	1.58E-03	1.50531290	0.00000000	1.33840130	0.18035841
253302_at	AT4G33660	2.36E-03	-1.52001000	0.00000000	-1.72918560	-0.32102203
253307_at	AT4G33670	9.24E-04	-0.41298437	-0.00000048	-0.39895964	0.06675673
253303_at	AT4G33780	4.15E-04	0.71861360	0.00000000	0.77207994	0.19493866
253283_at	AT4G34090	2.39E-03	-0.93628050	0.00000048	-1.15609840	-0.12674332
253282_at	AT4G34120	2.96E-03	0.75305176	0.00000000	0.80504704	0.26290607
253284_at	AT4G34150	1.52E-03	1.25245050	0.00000000	1.31633420	-0.06950092
253285_at	AT4G34250	1.43E-03	-0.75885606	0.00000000	-0.88483070	0.04904556
253257_at	AT4G34390	1.09E-03	1.51113270	0.00000000	1.48077250	0.08443356
253173_at	AT4G35110	1.58E-03	2.43097730	0.00000000	2.45077470	0.03197861
253181_at	AT4G35180	6.83E-04	2.71767520	0.00000000	2.66721400	0.09118557
253188_at	AT4G35300	2.59E-03	-0.73047450	-0.00000048	-0.51158380	0.32348108
253195_at	AT4G35420	1.10E-03	-0.49541760	0.00000024	-0.36557030	-0.10810280
253147_at	AT4G35600	1.74E-03	0.87736680	0.00000000	0.83210370	-0.11983705
253118_at	AT4G35950	1.72E-03	-0.55556010	0.00000000	-0.56525900	0.05405808
253075_at	AT4G36150	1.41E-03	0.94693420	-0.00000024	0.67162440	-0.15755415
246162_at	AT4G36400	2.05E-03	0.20999193	0.00000024	0.16348243	-0.05035353
246195_at	AT4G36410	1.05E-03	-0.87168810	0.00000000	-1.30127690	-0.33042622
246219_at	AT4G36760	1.26E-03	0.36060047	0.00000000	0.32572985	-0.12601566
246214_at	AT4G36988 /	1.56E-03	2.35888430	0.00000000	2.11177060	0.12904430
246194_at	AT4G37000	2.24E-03	-0.76121664	0.00000048	-0.75233746	0.16876459
246231_at	AT4G37080	3.12E-04	-1.04078580	-0.00000048	-1.02892610	0.16462612
253044_at	AT4G37290	3.36E-03	0.47903514	0.00000000	0.83462024	-0.23296070
253099_s_at	AT4G37520 /	3.59E-04	2.29499770	0.00000024	1.96266800	0.40176558
253063_at	AT4G37640	5.35E-04	1.79652550	0.00000000	1.60180330	0.08521128
253038_at	AT4G37790	2.22E-04	0.46649313	-0.00000024	0.38948680	0.02882218
252979_at	AT4G38225	4.21E-03	-0.65216160	0.00000000	-0.70707417	0.00334740
252981_at	AT4G38260	3.60E-03	1.22777560	0.00000000	1.19517370	0.19308686
252975_s_at	AT4G38430	1.42E-04	-0.86285020	-0.00000048	-0.72603416	-0.08746958
252977_at	AT4G38560	6.95E-04	1.41061020	0.00000000	1.45455100	-0.14777136
252971_at	AT4G38770	1.28E-03	-0.27136564	-0.00000048	-0.55751705	-0.02006197
252921_at	AT4G39030	6.87E-04	2.59823940	-0.00000024	2.74315880	0.20928931
252945_at	AT4G39140	1.58E-03	0.76778460	-0.00000024	0.67171380	0.31540490
252879_at	AT4G39390	1.42E-03	0.93310310	0.00000000	0.90203047	0.25146960
252906_at	AT4G39640	1.49E-03	0.18847800	0.00000000	0.07649994	-0.10899830
252908_at	AT4G39670	4.27E-04	2.64576150	0.00000000	2.44568870	0.01112890
252861_at	AT4G39820	1.23E-03	0.95721626	0.00000000	0.79033900	0.20227623
252862_at	AT4G39830	1.24E-03	2.01021960	0.00000000	2.10938430	0.01834202
252825_at	AT4G39890	3.02E-03	1.20655560	0.00000024	1.12512610	-0.07104135
252876_at	AT4G39970	2.73E-03	-0.91283990	0.00000000	-1.07883690	-0.10625982
251142_at	AT5G01015	1.02E-03	-1.82012560	-0.00000048	-1.96494630	-0.27070856
251089_at	AT5G01390	1.78E-03	-0.54920960	-0.00000048	-0.43128730	-0.37689400
251054_at	AT5G01540	2.58E-04	1.86956120	0.00000000	1.65321060	-0.22910666
251061_at	AT5G01830	3.34E-03	0.63082194	0.00000024	0.27551556	-0.01608515
251063_at	AT5G01850	1.11E-03	1.72911740	0.00000024	1.68508390	0.38428260
251068_at	AT5G01920	2.91E-04	-1.11918070	0.00000048	-1.17381100	-0.14094973
251033_at	AT5G01960	1.96E-03	0.35904170	0.00000048	0.30630684	-0.06462812
251077_at	AT5G01980	2.84E-03	0.59834910	0.00000000	0.64189625	-0.06113911
251037_at	AT5G02100	5.56E-06	1.14643430	0.00000000	0.80730580	0.00994825
251040_at	AT5G02250	3.21E-04	-0.54387045	-0.00000048	-0.44903898	0.04635191
250990_at	AT5G02290	2.10E-03	0.94710160	0.00000000	0.92808104	-0.12312651
251046_at	AT5G02370	1.98E-03	-0.66590047	0.00000024	-0.49931502	-0.23664379
250994_at	AT5G02490	1.12E-03	2.52060600	0.00000000	2.45360900	0.13877177
251013_at	AT5G02540	2.84E-03	-0.33089685	0.00000024	-0.85929300	-0.17079353

250998_at	AT5G02620	3.84E-04	0.44325447	0.00000000	0.33068037	-0.14995337
250999_at	AT5G02630	1.18E-04	0.49561740	0.00000000	0.41372870	0.02050400
251008_at	AT5G02710	3.18E-03	-0.85278320	0.00000000	-0.62108400	0.33253384
250934_at	AT5G03030	2.68E-04	0.26542377	0.00000000	0.17657518	-0.13752985
250930_at	AT5G03160	4.75E-04	1.52674910	-0.00000024	1.37842370	0.13813806
250899_at	AT5G03340	9.92E-04	0.98903656	-0.00000024	0.76294756	0.17972660
250918_at	AT5G03610	2.61E-03	1.02045010	0.00000000	0.84862230	0.22891665
250916_at	AT5G03630	3.25E-04	1.25091310	0.00000048	0.99639416	0.23910856
250879_at	AT5G04060	3.88E-03	-0.58176947	-0.00000024	-0.49285698	-0.10633612
245693_at	AT5G04260	4.19E-03	0.33243418	0.00000000	0.31486510	0.02832937
250849_at	AT5G04410	4.10E-03	0.27006817	0.00000048	0.22947788	-0.14267730
250829_at	AT5G04720	1.40E-03	1.85896020	0.00000000	1.85512210	0.09121180
250863_at	AT5G04750	3.07E-03	0.55163100	0.00000048	0.58776000	0.00627804
250856_at	AT5G04810	8.66E-04	-0.87205220	-0.00000048	-0.88804054	-0.10775137
250812_at	AT5G04900	1.57E-04	-0.47480774	0.00000000	-0.60536550	0.23063564
250831_at	AT5G04920	2.35E-03	0.53639410	0.00000000	0.49866152	-0.18611431
250818_at	AT5G04930	5.20E-04	1.30120610	0.00000048	1.28986020	-0.30514812
250821_at	AT5G05190	2.51E-03	0.46081114	0.00000024	0.40947342	-0.28367066
250796_at	AT5G05300	3.47E-05	1.92425780	0.00000000	1.60771370	-0.04908681
250775_at	AT5G05460	1.66E-05	1.00819060	-0.00000024	1.00363060	-0.41810608
250738_at	AT5G05730	7.48E-04	1.98452000	0.00000000	1.61634020	0.02546120
250763_at	AT5G06060	1.40E-03	-0.42688322	0.00000048	-0.48735762	0.09592438
250694_at	AT5G06710	2.06E-04	-0.67709970	0.00000024	-0.35250760	-0.02687550
250697_at	AT5G06800	3.50E-03	-0.25913525	0.00000024	-0.22873473	-0.00957584
250655_at	AT5G06960	2.93E-03	0.76801180	-0.00000024	0.68463254	0.10966396
250668_at	AT5G07020	2.52E-03	-0.42524672	-0.00000048	-0.51358270	0.17193270
250640_at	AT5G07150	3.87E-03	0.50600910	0.00000024	0.74697850	-0.16214705
250626_at	AT5G07350	4.27E-03	0.86210300	-0.00000048	0.69627476	-0.01416779
250627_at	AT5G07360	1.51E-03	0.77264833	-0.00000024	0.66991997	-0.19986248
250577_at	AT5G07910	1.94E-03	0.55702260	0.00000000	0.67706300	0.14621449
250569_at	AT5G08130	3.06E-03	-0.47678542	0.00000048	-0.59266305	0.03769016
250575_at	AT5G08240	1.41E-03	1.45303010	-0.00000024	1.52204560	-0.06208706
246053_at	AT5G08340	3.39E-04	-0.56779766	0.00000000	-0.46476007	-0.32164645
246036_at	AT5G08370	5.10E-04	-0.93172290	0.00000000	-0.67758870	0.10189295
250528_at	AT5G08600	2.79E-03	-0.25027156	-0.00000024	-0.20396209	0.12001324
250531_at	AT5G08650	3.14E-04	-0.55708410	0.00000000	-0.91382027	0.06493521
245932_at	AT5G09290	1.22E-03	0.85391260	0.00000000	1.24805570	-0.51147390
245881_at	AT5G09460 /	1.59E-03	-0.51847816	-0.00000024	-0.18096328	0.23176980
250516_at	AT5G09620	1.78E-04	0.44268894	0.00000048	0.33878660	0.00529480
250478_at	AT5G10250	2.91E-03	-0.66151550	0.00000000	-0.69932747	-0.02228975
250435_at	AT5G10380	2.81E-03	2.00994730	0.00000048	2.35454030	-0.00869227
250429_at	AT5G10470	2.26E-03	-0.60174800	-0.00000048	-0.72569130	0.11011410
246018_at	AT5G10695	8.12E-05	1.94063850	0.00000000	1.67715360	-0.34542322
250400_at	AT5G10740	2.44E-04	1.15322780	-0.00000024	0.85410595	-0.29407310
245906_at	AT5G11070	1.40E-03	-0.85684013	0.00000000	-0.73868610	0.26968430
250415_at	AT5G11210	1.95E-03	0.70061730	0.00000024	0.78999330	-0.16070032
250362_at	AT5G11380	2.67E-03	-0.34501170	0.00000024	-0.32422042	0.02987289
250339_at	AT5G11670	3.57E-03	1.15794090	0.00000048	1.03705120	-0.02162838
250334_at	AT5G11770	1.83E-03	0.27359295	0.00000000	0.42898893	0.05063868
250302_at	AT5G11920	1.47E-04	2.46568580	0.00000000	2.30549380	-0.03393436
250301_at	AT5G11970	1.69E-03	1.35794730	0.00000000	1.04795460	-0.13321209
250305_at	AT5G12150	6.90E-04	-0.65082600	0.00000000	-0.75650930	-0.07200527
245183_at	AT5G12440	4.20E-03	-0.56444764	0.00000048	-0.43342042	0.10683012
250264_at	AT5G12890	2.17E-04	0.70797825	0.00000024	0.71374510	-0.11715221
250267_at	AT5G12930	8.36E-04	1.24813320	0.00000000	1.04517150	0.02754235
250277_at	AT5G12940	7.12E-04	0.36225033	0.00000024	0.54938200	-0.04028750
250272_at	AT5G13000	2.04E-03	-0.51525400	-0.00000048	-0.40825224	-0.03392220
250276_at	AT5G13030	3.69E-03	0.60012150	-0.00000048	0.62198925	0.11100960
245976_at	AT5G13080	1.30E-03	1.87871000	-0.00000024	1.31728890	-0.13232470
250289_at	AT5G13190	2.94E-04	1.58472350	0.00000000	1.44510700	-0.14818573
250279_at	AT5G13200	1.50E-03	1.38794900	0.00000000	1.37071080	-0.04655981
250286_at	AT5G13320	8.76E-04	3.19903760	0.00000000	3.26682900	0.44817257
250261_at	AT5G13400	7.26E-04	-1.17285250	-0.00000048	-1.23963790	-0.12992048
250262_at	AT5G13410	2.82E-03	-0.57021904	0.00000000	-0.77806090	-0.27947140
245855_at	AT5G13550	3.98E-03	0.83332160	0.00000000	0.53244925	0.13655877
250255_at	AT5G13730	2.29E-03	-1.21543810	0.00000000	-1.46613000	-0.06250668
250151_at	AT5G14570	3.25E-03	-0.54733560	0.00000000	-0.75561760	-0.09379816
250146_at	AT5G14660	4.08E-03	-0.70839360	0.00000000	-0.63669680	0.35182762
246547_at	AT5G14970	8.01E-04	-0.55260515	0.00000000	-0.80367520	-0.00126457
250160_at	AT5G15210	1.50E-03	-0.32128860	0.00000000	-0.30474950	0.10952568
246565_at	AT5G15530	6.71E-04	-0.65542510	0.00000024	-0.58040690	0.05263948
246532_at	AT5G15870	1.11E-03	1.75245570	0.00000000	1.76964240	0.30223536
246483_at	AT5G16000	5.17E-05	-0.88623860	0.00000048	-0.78116800	-0.08035421
246488_at	AT5G16010	3.31E-04	-0.38154364	0.00000000	-0.22656060	0.00607586
246485_at	AT5G16080	6.58E-04	1.08543780	0.00000000	1.21362400	0.08633304
246498_at	AT5G16230	6.55E-04	2.39981910	0.00000024	2.08612970	0.25737810
250125_at	AT5G16390	1.55E-03	-0.48183870	-0.00000048	-0.48060130	0.13409472
250129_at	AT5G16450	3.51E-03	0.42717028	0.00000000	0.43214464	0.14402819
250102_at	AT5G16590	6.89E-04	-0.70113660	0.00000048	-0.76624180	-0.03911686
250105_at	AT5G16630	4.37E-04	-0.42749882	-0.00000024	-0.38740325	-0.23954177
246422_at	AT5G17060	3.93E-03	1.22103400	-0.00000024	1.15854020	0.27871084
250094_at	AT5G17380	7.25E-04	1.14452220	-0.00000048	0.94602110	0.06086636
246427_at	AT5G17400	1.83E-03	-0.87824535	0.00000000	-0.75330760	-0.20784068
246434_at	AT5G17520	1.90E-03	-0.76191900	0.00000048	-0.73213960	0.04506111
250056_at	AT5G17660	2.07E-03	-0.67338085	0.00000024	-0.38507295	-0.07605934
250075_at	AT5G17670	5.11E-04	-0.70886850	0.00000048	-0.95592546	0.01661778
250062_at	AT5G17760	1.33E-03	2.89295430	0.00000024	3.75268030	0.83878230
250054_at	AT5G17860	2.85E-03	1.81423900	0.00000000	1.10202500	0.02916336
250014_at	AT5G17990	1.48E-03	1.64698980	0.00000048	1.65030480	0.40348625

250016_at	AT5G18100	1.59E-03	-0.47415495	0.00000048	-0.49679660	-0.01242638
249988_at	AT5G18310	4.70E-04	1.03276920	-0.00000048	0.98386860	-0.00309753
250041_at	AT5G18410	3.41E-03	-0.64845324	0.00000000	-0.77361390	-0.23576832
249983_at	AT5G18470	9.00E-04	3.51239440	-0.00000024	3.34050270	0.23110962
249974_at	AT5G18780	1.07E-03	0.81285095	0.00000000	0.86241674	-0.23647094
249978_at	AT5G18850	3.15E-03	-0.69997810	0.00000048	-0.48336887	0.03023624
249957_at	AT5G18900	3.46E-03	0.69940186	0.00000048	0.78410390	0.12708235
249918_at	AT5G19240	1.86E-03	2.55816750	0.00000000	2.20767880	-0.21896935
245911_at	AT5G19690	3.36E-03	0.95113420	0.00000000	1.14650680	0.32756805
246150_at	AT5G19930	4.46E-04	0.87594890	0.00000000	0.84002350	-0.08703852
246143_at	AT5G19980	4.05E-03	1.16945740	0.00000000	1.25101380	0.05706501
246147_s_at	AT5G19990 /	2.34E-03	0.56475020	0.00000000	0.58068514	0.12074709
246110_at	AT5G20140	2.13E-03	-0.45706272	-0.00000024	-0.43506694	0.05096769
246099_at	AT5G20230	1.63E-03	2.41540000	0.00000000	2.22260240	-0.16652775
246122_at	AT5G20380	6.54E-04	-0.61427736	-0.00000048	-0.61045790	-0.03088617
246098_at	AT5G20400	3.05E-04	1.82328600	0.00000000	1.49752160	0.21825123
246135_at	AT5G20885	1.09E-03	-0.43878317	-0.00000048	-0.23836803	0.12895155
245686_at	AT5G22060	2.04E-03	1.53042170	0.00000000	1.52145530	0.17192698
249928_at	AT5G22250	3.81E-03	1.50076440	-0.00000024	1.17520880	-0.11265039
249940_at	AT5G22380	1.90E-03	1.37609530	-0.00000024	1.07552170	-0.13020515
249896_at	AT5G22530	4.48E-04	2.80511100	0.00000000	2.37407970	-0.05068708
249890_at	AT5G22570	2.08E-03	4.04358400	-0.00000024	3.62639710	0.44280982
249899_at	AT5G22620	3.81E-04	-0.67810726	0.00000048	-0.67615320	0.00411034
249910_at	AT5G22630	2.49E-04	1.25338790	0.00000024	1.04825020	0.14234757
249909_s_at	AT5G22770 /	4.54E-04	0.77461240	0.00000000	0.66808130	0.16995096
249866_at	AT5G23010	1.37E-03	-1.45718770	0.00000048	-0.65980150	0.02320814
249876_at	AT5G23060	4.28E-04	-0.98252060	-0.00000048	-1.06506590	0.19885015
249847_at	AT5G23210	2.15E-03	-0.98374080	0.00000000	-1.23604750	-0.27462770
249796_at	AT5G23540	1.14E-03	0.73052600	-0.00000048	0.84466124	0.19318676
249806_at	AT5G23850	4.33E-04	1.36307240	0.00000000	1.36812830	0.14439678
249810_at	AT5G23920	3.32E-03	-0.85405730	-0.00000048	-0.78199196	-0.08903647
249770_at	AT5G24110	4.83E-04	2.81863880	-0.00000024	2.46967890	-0.22015023
249777_at	AT5G24210	4.74E-04	2.83095740	0.00000000	2.81479880	0.02163124
249785_at	AT5G24300	6.69E-05	-0.77817106	-0.00000048	-0.53242400	0.24348688
249732_at	AT5G24420	2.41E-03	-1.71278550	-0.00000048	-1.29769780	0.30347633
249754_at	AT5G24530	4.55E-04	2.51503800	0.00000000	2.57594730	-0.00454712
249743_at	AT5G24540	3.21E-04	1.40799400	0.00000000	1.09109970	-0.10817289
249755_at	AT5G24580	2.12E-03	-0.45029163	0.00000000	-0.26249194	0.01456523
246976_s_at	AT5G24810	7.56E-04	1.19198560	0.00000048	1.10768800	0.14695597
246927_s_at	AT5G25250 /	1.51E-03	3.81783300	-0.00000024	3.52580880	0.48142480
246895_at	AT5G25540	1.30E-03	0.82848790	0.00000048	0.70297720	0.07186365
246858_at	AT5G25930	5.10E-04	2.25264170	-0.00000024	1.74337150	-0.18734646
246870_at	AT5G26030	3.12E-03	1.52231070	0.00000048	1.50376940	0.25896215
246847_at	AT5G26820	3.99E-03	-0.79254150	0.00000000	-1.05189940	-0.04625750
246849_at	AT5G26850	6.75E-04	-0.33785105	0.00000048	-0.14132404	0.15558720
246821_at	AT5G26920	2.48E-03	2.64328770	0.00000000	3.06657310	0.44795465
246792_at	AT5G27290	2.49E-04	-1.06865930	0.00000048	-1.08666610	0.05175448
246777_at	AT5G27420	1.22E-03	2.24474900	0.00000000	2.34462070	-0.06039286
246779_at	AT5G27520	1.91E-03	0.87236166	0.00000000	0.71495580	0.03865504
246736_at	AT5G27560	8.63E-05	-0.79221725	0.00000048	-0.74386406	0.13567162
246789_at	AT5G27600	3.82E-04	1.10237690	-0.00000024	0.95450974	-0.31091976
246749_at	AT5G27830	8.59E-04	1.33529950	0.00000024	1.33968260	0.23551488
246682_at	AT5G33290	1.33E-03	1.04557700	0.00000048	0.90775300	-0.03470135
255859_at	AT5G34930	2.95E-03	0.46070457	0.00000000	0.36903167	-0.15205574
246653_at	AT5G35200	3.42E-03	0.67558575	0.00000000	0.77904654	-0.01960087
246654_s_at	AT5G35210 /	1.12E-03	-0.27771378	0.00000000	-0.20556402	0.38927984
249691_at	AT5G36170	1.50E-04	-0.70092820	0.00000000	-0.87830590	-0.09051943
246620_at	AT5G36220	2.02E-03	2.25728560	0.00000000	1.67497440	-0.13083196
246621_at	AT5G36230	2.09E-03	-0.14943647	0.00000000	-0.02134943	0.04821587
249638_at	AT5G36880	3.74E-03	1.01475860	-0.00000048	0.97401047	0.17222070
249639_at	AT5G36930	3.93E-03	0.79286600	0.00000000	0.94617033	-0.23807550
249652_at	AT5G37070	1.32E-03	1.14914560	0.00000000	1.01340630	0.07717037
249610_at	AT5G37360	9.23E-04	-0.44921780	-0.00000048	-0.66879654	-0.04939365
249625_at	AT5G37480	6.75E-04	0.81114817	0.00000000	0.67409610	-0.16296577
249618_at	AT5G37490	3.33E-04	0.15203261	0.00000024	0.31566024	-0.13634300
249581_at	AT5G37600	1.69E-03	1.80195380	0.00000000	2.02633240	0.56672764
249579_at	AT5G37680	3.30E-03	-0.80825780	0.00000000	-0.71636033	-0.18670702
249582_at	AT5G37780	2.34E-03	0.67071724	0.00000048	0.74977160	0.18488741
249554_at	AT5G38290	2.72E-03	-0.80229354	0.00000000	-0.80248594	0.16180325
249510_at	AT5G38510	2.21E-03	-0.95992090	0.00000000	-1.07780120	-0.07825327
249524_at	AT5G38520	1.02E-03	-0.97117376	0.00000000	-1.04845810	0.15692711
249523_at	AT5G38630	3.82E-03	0.65014980	0.00000048	0.60936450	0.03883553
249525_at	AT5G38650	6.30E-04	0.68483590	0.00000000	0.56375840	-0.03630018
249527_at	AT5G38710	2.21E-04	2.39893840	0.00000024	1.75863030	-0.09690380
249537_at	AT5G38830	3.97E-03	0.53903960	0.00000000	0.53611183	0.24229717
249481_at	AT5G38900	1.77E-03	2.94982670	0.00000000	2.73633770	0.00597048
249480_s_at	AT5G38990 /	1.08E-03	0.70427560	0.00000000	0.44603777	-0.12449169
249485_at	AT5G39020	6.51E-04	0.90113354	0.00000000	0.66926813	-0.25363730
249486_at	AT5G39030	3.09E-03	0.40550566	0.00000000	0.47131157	-0.21359539
249487_at	AT5G39040	5.86E-04	0.74737170	-0.00000024	0.72206783	-0.00276279
249489_at	AT5G39090	3.16E-03	0.80696680	0.00000000	0.72077775	-0.29707742
249453_at	AT5G39510	2.55E-03	0.54714730	0.00000000	0.57217360	0.02505159
249417_at	AT5G39670	7.67E-04	3.06385800	0.00000000	3.17365220	0.27089143
249423_at	AT5G39785	2.25E-03	1.06567360	0.00000000	0.95072390	0.10476971
249385_at	AT5G39950	1.95E-03	1.05641510	0.00000000	1.06784870	0.00741196
249393_at	AT5G40170	4.29E-04	1.06802610	0.00000000	0.87855480	0.16981030
249397_at	AT5G40230	2.13E-03	0.35537648	0.00000024	0.92333674	0.17823195
249410_at	AT5G40380	3.18E-05	-0.82553580	0.00000000	-1.18512730	-0.35618448
249374_at	AT5G40580	2.34E-04	0.68661020	0.00000048	0.63816020	0.07168245

249343_at	AT5G40650	3.39E-03	0.52264404	0.00000000	0.45824242	0.06527472
249372_at	AT5G40760	3.14E-03	1.62569670	-0.00000024	1.57658910	0.39025617
249346_at	AT5G40780	7.02E-04	2.12858770	0.00000000	1.99451920	0.07772255
249288_at	AT5G41050	3.27E-03	-1.15468290	-0.00000048	-1.27450700	0.09784699
249290_at	AT5G41060	1.53E-03	-0.48640227	-0.00000024	-0.30634308	0.11541915
249314_at	AT5G41180	1.24E-03	1.09574440	0.00000024	1.06510260	0.16920924
249255_at	AT5G41610	4.41E-04	0.52421500	0.00000000	0.67755985	0.12694645
249279_at	AT5G41920	3.14E-03	-0.23335266	0.00000000	-0.23350906	0.08686638
249252_at	AT5G42010	3.36E-03	0.59077310	0.00000000	0.74613190	0.06605625
249237_at	AT5G42050	7.01E-04	1.62328480	0.00000000	1.74294570	0.09295893
249230_at	AT5G42070	6.01E-04	-1.28585620	0.00000000	-1.20780320	-0.16590881
249234_at	AT5G42200	1.13E-04	-1.06467890	0.00000000	-1.28767800	-0.19517231
249197_at	AT5G42380	8.25E-04	1.75537750	0.00000012	1.94641450	-0.04053497
249212_at	AT5G42690	1.06E-03	-0.23656869	0.00000000	-0.20494652	-0.10555673
249161_at	AT5G42790	3.57E-03	1.00522140	0.00000048	0.99900720	0.29523468
249188_at	AT5G42830	2.88E-04	1.20908690	0.00000000	1.16124150	-0.38926172
249177_at	AT5G42850	3.51E-03	0.42152310	0.00000048	0.35097600	0.12297440
249130_at	AT5G43100	6.10E-05	0.49140453	0.00000000	0.53665830	-0.16070557
249120_at	AT5G43750	3.90E-04	-0.96342325	0.00000000	-1.04447790	-0.04262638
249096_at	AT5G43910	2.62E-04	2.02160500	-0.00000024	2.05910920	0.03368759
249077_at	AT5G43940	1.73E-03	0.46666288	-0.00000048	0.68250800	0.06616545
249075_at	AT5G44000	1.47E-03	-0.48044920	0.00000000	-0.40325785	-0.11374426
249069_at	AT5G44010	3.15E-03	-0.58828900	-0.00000024	-0.52199270	0.29118800
249078_at	AT5G44070	3.13E-04	1.06197790	0.00000048	1.00097610	-0.12541294
249083_at	AT5G44140	5.53E-04	-0.40034652	0.00000000	-0.18183088	-0.07852650
249089_at	AT5G44240	5.56E-04	0.82764006	-0.00000048	0.49162388	-0.29448557
249052_at	AT5G44420	2.01E-03	3.36235760	-0.00000024	3.04722740	-0.97673010
249002_at	AT5G44520	4.25E-04	-0.58746720	0.00000000	-0.72692490	0.03712559
249004_at	AT5G44570	3.90E-03	1.69240710	0.00000000	1.62374830	0.13295865
254521_at	AT5G44820	2.74E-03	2.14009000	0.00000000	1.61222150	-0.17581868
249021_at	AT5G44820	2.43E-03	1.30580380	0.00000000	1.30390020	-0.03176332
249001_at	AT5G44990	3.55E-03	0.91534950	0.00000000	0.46442270	-0.37729645
248971_at	AT5G45000	1.93E-05	1.72479250	0.00000024	1.51700640	-0.21213889
248975_at	AT5G45040	9.36E-04	-1.21143030	-0.00000048	-1.28333350	-0.03300095
248981_at	AT5G45110	1.78E-03	1.31309220	0.00000000	1.29094840	-0.12600231
248983_at	AT5G45130	3.78E-03	1.02410030	0.00000048	1.01673510	0.21551037
248997_at	AT5G45300	1.02E-03	-0.66915700	0.00000000	-0.47434520	-0.08694601
248967_at	AT5G45350	4.01E-03	-0.16859531	0.00000048	-0.10989904	-0.03378248
248941_s_at	AT5G45460 /	2.74E-03	1.57435890	0.00000024	1.70039960	0.49922514
248942_at	AT5G45480	8.84E-04	0.91797540	-0.00000048	0.92115736	0.07356072
248944_at	AT5G45500	3.21E-03	1.13540700	-0.00000048	1.02677200	-0.19026184
248951_at	AT5G45550	2.91E-03	0.70186330	0.00000048	0.57254460	-0.00511932
248962_at	AT5G45680	5.49E-04	-0.96726704	0.00000048	-1.15060900	-0.12363434
248932_at	AT5G46050	2.34E-03	1.72836540	0.00000000	1.63062620	0.18226194
248934_at	AT5G46080	5.60E-04	1.19155120	0.00000024	1.08940670	-0.20069313
248905_at	AT5G46250	2.71E-04	0.63456820	0.00000048	0.56691120	-0.04221010
248896_at	AT5G46350	3.03E-03	0.66998600	0.00000000	0.79707265	-0.17853689
248899_at	AT5G46390	2.19E-03	-0.53267956	0.00000048	-0.47609830	-0.10182619
248906_at	AT5G46420	6.66E-04	-0.65092470	0.00000000	-0.74183080	0.03682470
248821_at	AT5G47070	3.31E-03	1.24300430	0.00000024	1.32285360	0.08038497
248833_at	AT5G47120	1.27E-03	1.50151200	0.00000000	1.60487600	0.30202007
248792_at	AT5G47200	9.71E-04	0.83225346	-0.00000048	0.92173815	0.26935863
248794_at	AT5G47220	7.00E-04	2.79037100	0.00000024	2.45559400	0.11047530
248787_at	AT5G47420	4.20E-03	0.88619660	-0.00000048	0.82837060	0.10079718
248766_at	AT5G47580	8.04E-04	0.84059880	0.00000000	0.66839430	-0.08989358
248765_at	AT5G47650	5.05E-04	-0.49014830	0.00000000	-0.42741728	-0.15530610
248769_at	AT5G47730	3.66E-04	0.88929915	0.00000000	0.86008763	0.01658464
248775_at	AT5G47850	1.61E-04	0.80305123	0.00000000	1.06741860	-0.05465722
248719_at	AT5G47910	1.46E-03	1.33887100	-0.00000048	1.22659020	0.04294729
248726_at	AT5G47960	1.47E-04	0.75367950	0.00000024	0.79341245	0.22889805
248698_at	AT5G48380	4.57E-04	1.64992140	0.00000048	1.69677260	0.25022173
248656_at	AT5G48460	1.31E-03	-0.78774405	0.00000000	-0.83921385	-0.24771404
248686_at	AT5G48540	5.46E-04	2.55560300	-0.00000048	2.21923110	0.01616526
248624_at	AT5G48790	1.44E-03	-0.86011505	0.00000048	-1.22943450	-0.07117701
248615_at	AT5G49570	2.66E-03	1.41946320	-0.00000024	1.20880370	0.15009070
248562_at	AT5G49680	2.20E-04	0.86555960	0.00000000	0.50047590	-0.15135312
248574_at	AT5G49830	2.14E-03	0.45256567	-0.00000048	0.44689655	0.03715515
248580_at	AT5G49890	1.62E-03	0.23571491	0.00000048	0.23171377	-0.04096651
248531_at	AT5G49950	2.98E-03	0.41068697	0.00000024	0.41622830	0.04704332
248547_at	AT5G50280	3.06E-03	-0.65208983	-0.00000024	-0.63484170	0.21305513
248521_s_at	AT5G50520 /	2.55E-03	0.60360480	0.00000048	0.47956753	-0.25945997
248523_s_at	AT5G50580 /	3.27E-03	-0.41817617	0.00000000	-0.28325891	-0.04751110
248442_at	AT5G51280	1.65E-03	0.49818230	-0.00000048	0.43721914	0.03550911
248409_at	AT5G51540 /	1.12E-04	-0.73214960	-0.00000048	-0.72571470	-0.11160040
248422_at	AT5G51640	2.01E-03	0.80177450	0.00000000	0.68063736	-0.03040171
248426_at	AT5G51740	3.31E-04	0.60438013	0.00000000	0.74798630	-0.06539202
248381_at	AT5G51830	1.65E-03	1.30708890	0.00000024	1.27942610	0.08224344
248402_at	AT5G52100	6.67E-04	-1.00544330	0.00000000	-1.02498580	0.09705210
248346_at	AT5G52210	9.34E-04	0.30822754	0.00000024	0.27930164	-0.24540377
248349_at	AT5G52240	4.28E-03	0.48667860	0.00000000	0.52788734	0.05772638
248336_at	AT5G52420	7.43E-05	-0.69049170	0.00000000	-0.66705940	0.27408028
248338_at	AT5G52440	2.95E-03	-0.76854470	-0.00000048	-0.70584726	-0.08831167
248313_at	AT5G52580	2.59E-03	0.42308950	0.00000000	0.46447563	-0.12586069
248332_at	AT5G52640	1.75E-04	2.58598330	-0.00000024	2.09115740	-0.30512810
248327_at	AT5G52750	6.15E-04	2.52641250	0.00000024	2.33535480	-0.23067951
248322_at	AT5G52760	1.00E-03	3.37972700	0.00000000	3.45464180	0.25401950
248330_at	AT5G52810	1.53E-03	2.85455800	0.00000024	2.89220570	0.33065915
248285_at	AT5G52960	3.29E-03	-0.65313053	-0.00000048	-0.66614676	0.08856773
248287_at	AT5G52970	5.01E-05	-1.04513500	0.00000000	-1.01498170	0.13652611

248298_at	AT5G53110	1.76E-03	1.04356170	0.00000000	1.23761700	0.18713236
248224_at	AT5G53490	4.28E-03	-0.78054047	0.00000000	-0.83753586	0.04026890
248242_at	AT5G53580	1.24E-03	-0.71943570	-0.00000048	-0.64325476	0.19051409
248235_at	AT5G53860	4.34E-05	-0.65127230	0.00000000	-0.38173485	0.28460120
248192_at	AT5G54140	2.42E-03	0.63134980	0.00000024	0.63776230	-0.26347065
248181_at	AT5G54290	1.17E-03	-0.33745670	0.00000048	-0.50698710	-0.06608772
248164_at	AT5G54490	5.03E-05	2.36441200	0.00000024	2.23795700	0.07663822
248169_at	AT5G54610	4.57E-04	2.59593200	0.00000024	2.46999300	-0.11624646
248131_at	AT5G54830	3.21E-03	0.29378033	0.00000000	0.40958643	0.03498626
248132_at	AT5G54840	3.59E-03	1.06597190	0.00000000	0.66891384	-0.28862453
248134_at	AT5G54860	7.59E-04	1.82532980	0.00000048	1.59943870	-0.17363548
248108_at	AT5G55130	1.26E-03	0.55056430	0.00000000	0.53512480	-0.14410925
248092_at	AT5G55170	7.93E-04	1.45379730	0.00000000	1.25479510	-0.38471174
248058_at	AT5G55530	2.40E-03	0.39367342	-0.00000048	0.45463657	0.14053822
248068_at	AT5G55610	3.81E-03	0.98152830	0.00000048	1.09798430	0.42139053
248037_at	AT5G55930	2.32E-03	0.67086697	0.00000000	0.67882110	-0.27631617
248042_at	AT5G55960	5.73E-04	-0.55316734	0.00000000	-0.41453838	0.20520020
248007_at	AT5G56260	1.63E-03	0.85735035	0.00000048	0.85956097	0.21777344
247966_at	AT5G56610	1.18E-03	0.94839644	0.00000024	0.79331780	0.12129664
247936_at	AT5G57030	2.80E-03	-0.34779167	0.00000048	-0.42540407	0.27811003
247949_at	AT5G57220	3.50E-03	3.28715660	0.00000000	2.68311880	-0.16887927
247848_at	AT5G58120	1.48E-03	1.14295410	0.00000024	1.37432980	-0.14026070
247858_at	AT5G58220	2.00E-03	0.59993840	0.00000000	0.49433756	-0.20662403
247816_at	AT5G58260	2.27E-03	-1.04243610	0.00000000	-1.25596860	0.08558893
247740_at	AT5G58940	3.51E-04	1.43252610	0.00000000	1.45309020	0.13762140
247760_at	AT5G59130	3.92E-03	-0.93391680	0.00000000	-0.82224345	-0.09652710
247721_at	AT5G59140	7.35E-04	0.49915123	0.00000000	0.48214626	-0.13142347
247716_at	AT5G59350	1.28E-03	-0.86998770	-0.00000048	-0.57134030	0.17007160
247618_at	AT5G60280	1.19E-05	1.99756500	0.00000000	1.68360520	-0.04690695
247594_at	AT5G60800	5.31E-04	1.46523100	-0.00000024	1.54622410	-0.45229006
247604_at	AT5G60950	1.49E-03	1.98747870	-0.00000024	1.95397420	-0.36725855
247554_at	AT5G61010	1.56E-05	1.51339480	0.00000000	1.50389000	0.03489661
247556_at	AT5G61040	3.33E-03	-0.30093740	0.00000024	-0.28707004	0.10465717
247564_at	AT5G61140	2.76E-03	0.16837835	-0.00000024	0.17153430	-0.03258896
247573_at	AT5G61160	1.43E-03	3.35763120	-0.00000024	2.53427170	-0.13627195
247529_at	AT5G61520	2.61E-03	1.11980340	0.00000000	0.86553144	0.00378037
247493_at	AT5G61900	2.81E-05	1.73136000	-0.00000024	1.59369750	-0.00154638
247461_at	AT5G62100	3.00E-04	-0.39873457	0.00000000	-0.44780684	-0.12762380
247486_at	AT5G62140	4.63E-04	-1.18220160	0.00000000	-1.06458710	0.27414465
247478_at	AT5G62360	1.72E-03	-1.11128140	-0.00000048	-0.90370846	-0.21894836
247427_at	AT5G62580	3.08E-04	-0.44414950	0.00000000	-0.29238940	0.04090309
247439_at	AT5G62670	6.05E-04	-1.02564290	0.00000048	-1.16690160	-0.05327463
247401_at	AT5G62790	3.62E-04	-0.64317894	0.00000048	-0.43561792	0.24347400
247422_at	AT5G62810	1.45E-03	-0.38049412	0.00000000	-0.23133612	-0.02769041
247418_at	AT5G63030	2.37E-03	0.73555994	-0.00000048	0.67719220	-0.00977898
247389_at	AT5G63490	1.79E-03	0.94878197	0.00000000	1.07072210	0.23466635
247338_at	AT5G63680	2.48E-03	1.85437110	0.00000000	1.62341830	0.38692093
247347_at	AT5G63780	3.96E-05	-1.53823280	0.00000000	-1.32207680	-0.03734398
247314_at	AT5G64000	4.78E-04	2.71046160	0.00000000	2.78803160	0.20324564
247287_at	AT5G64230	3.69E-03	-0.66266180	0.00000024	-0.49950410	-0.31467152
247285_at	AT5G64400	1.54E-03	0.92394020	0.00000000	0.76427030	0.04211998
247293_at	AT5G64510	3.08E-04	2.62384220	-0.00000024	2.52183720	0.35806656
247214_at	AT5G64850	2.69E-03	-1.11423970	0.00000000	-1.11319300	-0.03127527
247216_at	AT5G64860	3.17E-03	-1.02123830	0.00000000	-0.86352970	-0.13586044
247208_at	AT5G64870	1.08E-03	0.85410810	0.00000000	0.61707160	-0.16174603
247215_at	AT5G64905	2.25E-03	1.50585460	-0.00000024	1.05160070	-0.15820813
247228_at	AT5G65140	2.79E-04	1.24835060	0.00000000	1.10436300	0.28031445
247201_at	AT5G65220	3.53E-03	-0.48779440	0.00000000	-0.43802547	0.07526350
247145_at	AT5G65600	2.50E-03	0.97133830	-0.00000024	0.71196030	-0.20170999
247080_at	AT5G66140	3.53E-03	0.72628546	0.00000000	0.93067360	0.30782747
247100_at	AT5G66520	1.05E-03	-1.35016010	0.00000000	-1.35169170	0.02734375
247071_at	AT5G66640	2.04E-04	1.61797570	0.00000000	1.62842300	-0.13027048
247069_at	AT5G66920	2.17E-04	-0.57872176	-0.00000048	-0.46630670	0.04412317
247034_at	AT5G67260	3.85E-03	-0.77406645	0.00000000	-0.71698380	0.10928011
246984_at	AT5G67310	7.66E-04	2.84720040	-0.00000024	2.00131250	-0.06844091
246988_at	AT5G67340	4.95E-04	2.21562900	-0.00000024	2.17448040	-0.03453255
246998_at	AT5G67370	5.80E-04	-0.63876677	0.00000048	-0.76495886	0.47351074
246996_at	AT5G67420	1.77E-03	-0.21533775	-0.00000048	-0.44110012	0.15643406
246993_at	AT5G67450	3.41E-04	2.06582070	0.00000000	1.85523410	0.13921952
266212_at		3.88E-03	-0.24562263	0.00000000	-0.19935298	-0.07526517
265526_x_at		1.92E-03	-0.19553232	-0.00000024	-0.34177970	-0.04725266
264824_at		1.83E-04	-0.67472935	-0.00000048	-0.50309370	0.13993359
264270_at		8.28E-05	-0.53365517	-0.00000024	-0.48614025	0.10819769
245491_at		5.01E-04	-0.21962023	0.00000024	-0.01723218	0.08527494
245079_at		2.57E-03	0.27364420	0.00000000	0.08985543	-0.18751812

Notes : Created from Advanced Analysis operation: significance Analysis, Genespring GX.
#Entitylist : Filtered on Expression (20.0 - 100.0)th Percentile in the Raw Data
#Interpretation : genotype - Treatment
#Experiment: arr345689 Hpa
#p-value cut-off:0.05
#Selected Test : 2way ANOVA
#p-value computation: Asymptotic
#Multiple Testing Correction: Benjamini-Hochberg
Technology : Affymetrix.GeneChip.ATH1-121501
